# Genotyping‐by‐sequencing informs conservation of Andean palms sources of non‐timber forest products

**DOI:** 10.1111/eva.13765

**Published:** 2024-07-31

**Authors:** Nicolás Peñafiel Loaiza, Abigail H. Chafe, Mónica Moraes R, Nora H. Oleas, Julissa Roncal

**Affiliations:** ^1^ Department of Biology Memorial University of Newfoundland St. John's Newfoundland and Labrador Canada; ^2^ Herbario Nacional de Bolivia, Instituto de Ecología Universidad Mayor de San Andrés La Paz Bolivia; ^3^ Centro de Investigación de la Biodiversidad y Cambio Climático – BioCamb e Ingeniería en Biodiversidad y Recursos Genéticos, Facultad de Ciencias de Medio Ambiente Universidad Indoamérica Quito Ecuador; ^4^ Present address: Chone y Babahoyo Loja Ecuador

**Keywords:** Arecaceae, genome‐wide SNPs, IUCN endangered, management units, non‐timber forest product, population genomics

## Abstract

Conservation and sustainable management of lineages providing non‐timber forest products are imperative under the current global biodiversity loss. Most non‐timber forest species, however, lack genomic studies that characterize their intraspecific variation and evolutionary history, which inform species' conservation practices. Contrary to many lineages in the Andean biodiversity hotspot that exhibit high diversification, the genus *Parajubaea* (Arecaceae) has only three species despite the genus' origin 22 million years ago. Two of the three palm species, *P. torallyi* and *P. sunkha*, are non‐timber forest species endemic to the Andes of Bolivia and are listed as IUCN endangered. The third species, *P. cocoides*, is a vulnerable species with unknown wild populations. We investigated the evolutionary relationships of *Parajubaea* species and the genetic diversity and structure of wild Bolivian populations. Sequencing of five low‐copy nuclear genes (3753 bp) challenged the hypothesis that *P. cocoides* is a cultigen that originated from the wild Bolivian species. We further obtained up to 15,134 de novo single‐nucleotide polymorphism markers by genotyping‐by‐sequencing of 194 wild *Parajubaea* individuals. Our total DNA sequencing effort rejected the taxonomic separation of the two Bolivian species. As expected for narrow endemic species, we observed low genetic diversity, but no inbreeding signal. We found three genetic clusters shaped by geographic distance, which we use to propose three management units. Different percentages of missing genotypic data did not impact the genetic structure of populations. We use the management units to recommend in situ conservation by creating new protected areas, and ex situ conservation through seed collection.

## 1 INTRODUCTION

Worldwide, non‐timber forest products contribute significantly to the livelihood of millions of people from both rural and urban communities (Hurley & Emery, 2018; Wahlén, 2017). Non‐timber forest products help to fulfill basic subsistence and consumption needs ranging from energy, nutrition and medicine to construction. Furthermore, they provide regular cash representing from 10% to 60% of the total household income in African, Asian and American communities (Godoy et al., 2002; Heubach et al., 2011; Shackelton & Pandey, 2014). Given the current global biodiversity loss, many economically important species are threatened with extinction, therefore, conservation and sustainable use of non‐timber forest species are imperative. The need to protect these resources should go beyond the traditional conservation practices that focus on species‐level approaches and should include methods that investigate intraspecific diversity (Coates et al., 2018).

Many species in the palm family (Arecaceae) are considered a ‘biocultural keystone’ since they provide ecological and cultural services (Shackleton et al., 2018). Palms are often regarded as ecological keystones because they produce highly nutritious fruit year‐round for fauna (Peres, 2000; Terborgh, 1986), and a cultural keystone because they are regarded together with grasses and legumes as one of the three most economically important plant families to humanity (Dransfield et al., 2008). Rural tropical societies depend on palms for a wide diversity of needs (Macía et al., 2011; Moraes et al., 2015; Paniagua‐Zambrana et al., 2007), making many palm species important sources of non‐timber forest products. In South America, palms are mostly harvested from their natural habitat and in many cases in an unsustainable way (38 out of the 97 species surveyed by Bernal et al., 2011).

Earlier evolutionary and population genetic studies of biocultural keystone palms have successfully guided conservation actions through the creation of protected areas, collection of seeds for ex situ conservation and recommendations for translocations or introductions (e.g. Asmussen‐Lange et al., 2011; González‐Pérez et al., 2004; Namoff et al., 2011; Sanín et al., 2017; Shapcott et al., 2007). Many studies, however, have not used next‐generation sequencing to discover single‐nucleotide polymorphisms (SNPs), which have become desirable molecular markers due to their abundance, wide genomic coverage, access to neutral regions and loci under selection, low error rates, and in many cases do not need a reference genome (Boutet et al., 2016; Helyar et al., 2011). Only recently, SNPs have been used in palms to reveal intraspecific diversity (Table 1). For example, the discovery of 929 neutral SNPs in wild populations of *Copernicia prunifera* (carnaúba palm) helped to propose the establishment of large in situ conservation areas for capturing the moderate genetic diversity found in this species (Costa et al., 2022). Furthermore, an excess instead of a deficit in heterozygosity, suggesting no inbreeding, was found compared to an earlier study using inter‐simple sequence repeats as markers (dos Santos et al., 2021; Table 1).

**TABLE 1:** Examples of plant case studies showing the efficiency of next‐generation sequencing‐derived single‐nucleotide polymorphisms (SNPs) for revealing intraspecific diversity, refining species delimitations and/or inferring wild relatives of economically important plants.

Species	Technique	# markers	Main result	Reference
*Copernicia prunifera* (carnaúba palm)	Genotyping‐by‐sequencing	929 neutral SNPs	Moderate genetic diversity (He = 0.201 to 0.265). Low to high genetic differentiation between populations (*F*_ST_ = −0.003 to 0.21). Heterozygote excess (*F*_IS_ = −0.553 to −0.102)	Costa et al. (2022)
*Copernicia prunifera* (carnaúba palm)	Inter‐simple sequence repeats	7 primers 101 loci	Moderate genetic diversity mean Nei's *h* = 0.213, and Shannon's index *I* = 0.312. Low to high genetic distance between populations (theta‐II = 0.005 to 0.657). Deficit in heterozygosity indicated genetic bottlenecks	dos Santos et al. (2021)
Genus *Acrocomia* (Arecaceae)	Genotyping‐by‐sequencing	3227 neutral SNPs	Contributed to species delimitation, recognized four species. Unknown sister of the incipiently domesticated *A. aculeata*. Low genomic diversity (He = 0.005 to 0.106). Moderate to high genetic differentiation between species (*F*_ST_ = 0.083 to 0.946). High levels of inbreeding in most *Acrocomia* species (*F*_IS_ = −0.145 to 0.591).	Díaz et al. (2021)
*Acrocomia aculeata* (macauba palm for oil)	Genotyping‐by‐sequencing	3259 neutral SNPs	Two genetic groups. Three geographic barriers to gene flow. Low genomic diversity (He = 0.005 to 0.081). None to high levels of inbreeding (*F*_IS_ = −0.139 to 0.377)	Díaz et al. (2021)
*Acrocomia aculeata* (macauba palm for oil)	Microsatellites	6	Moderate genetic diversity (Ho = 0.31 to 0.48). Evidence of inbreeding (Ho < He). Moderate to high population differentiation (*F*_ST_ = 0.152 to 0.314)	Lanes et al. (2015)
*Elaeis guineensis* (oil palm	Single locus amplified fragment sequencing	130,414 SNPs	Moderate genetic diversity (He = 0.29 to 0.33). Evidence of inbreeding (Ho < He).	Xia et al. (2019)
*Cocos nucifera* (coconut palm)	Genotyping‐by‐sequencing	10,835 SNPs	Moderate to high genomic diversity (He = 0.019 to 0.872). High population differentiation (*F*_ST_ = 0.206 to 0.208)	Rajesh et al. (2021)
*Dioscorea alata* (yam	Genotyping‐by‐sequencing	6017 bi‐allelic SNPs	Low genotypic richness (*R* = 17.98 to 39.99) and differentiation (*F*_ST_ = 0.001 to 0.055) across continental gene pools. Heterozygote excess (*F*_IS_ = −0.16 to −0.07)	Sharif et al. (2020)
*Dioscorea alata* (yam)	Microsatellites	24	Moderate to high levels of polymorphism (He = 0.2 to 0.86). Slight heterozygote deficit (*F*_IS_ = −0.28 to 0.34)	Arnau et al. (2017)
Genus *Coffea* (Rubiaceae)	Genotyping‐by‐sequencing	28,800 nuclear SNPs	Well‐resolved phylogeny of *Coffea. Coffea brevipes* is the closest wild relative of *Coffea canephora* (coffee).	Hamon et al. (2017)
*Coffea arabica*, *C. canephora, C. excelsa* (coffee)	Whole genome sequencing	15,367,960 SNPs	*C. arabica* has the greatest nucleotide diversity among the three species (π = 3.81 × 10^−3^). Strong among species divergence (*F*_ST_ = 0.23 to 0.34)	Huang et al. (2020)

*Note*: Some examples include a comparison with other molecular markers like microsatellites and inter‐simple sequence repeats.

Single nucleotide polymorphisms have also contributed to the taxonomic controversy around species boundaries in palm genera like *Acrocomia*, *Brahea*, *Washingtonia* and *Geonoma* (Díaz et al., 2021; Klimova et al., 2018; Olivares et al., 2024). In other plant families (e.g. Rubiaceae: *Coffea*), SNPs achieved a higher genetic resolution than with other molecular markers like the internal transcribed spacer and intergenic plastid markers (Hamon et al., 2017; Huang et al., 2020; Table 1). SNPs unmasked a higher genetic diversity in *Coffea arabica* compared to the other two most commonly cultivated coffee species (*C. canephor*a and *C. excelsa*) and uncovered a strong genetic divergence among these three species (Huang et al., 2020).

**FIGURE 1:**
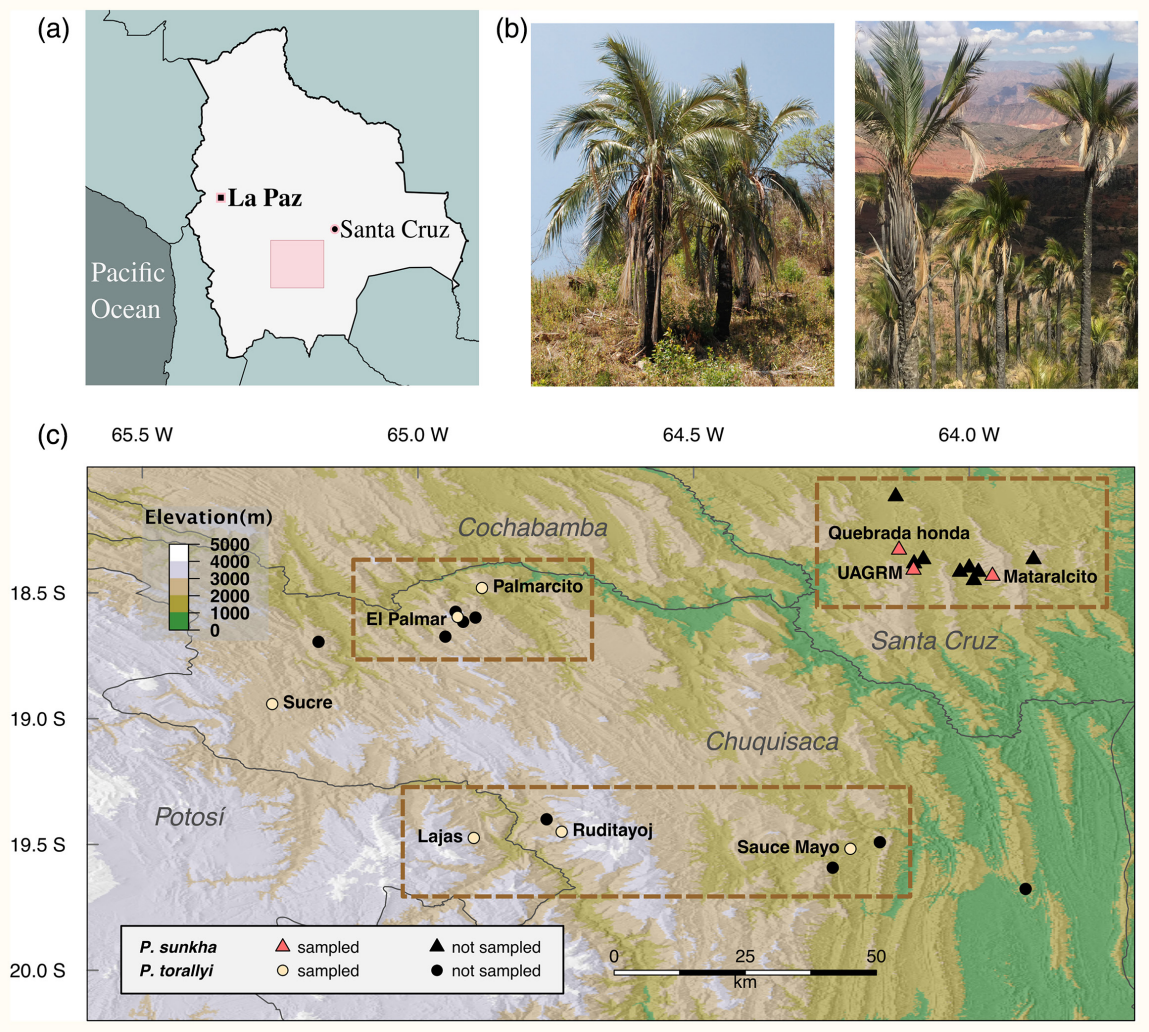
(a) Map of Bolivia within the South American continent indicating main cities and the area of the map in C (pink rectangle). (b) Photos of *P. sunkha* (left) and *P. torallyi* (right) in the wild from Mataralcito and Ruditayoj collection sites, respectively. (c) Map showing the geographical distribution of all known wild and cultivated *Parajubaea* populations in Bolivia, and the collection sites for this study. Departments' names are shown in grey. Dashed‐line bordered rectangles represent the proposed management units; sites that were not sampled are included due to their proximity to the collection sites, but their genetic makeup is unknown. Map created with digital elevation data from the NASA Shuttle Radar Topographic Mission (SRTM) available at https://srtm.csi.cgiar.org/. The coordinate system used was EPSG:4326 and WGS84 datum.

*Parajubaea* Burret is a palm genus (subtribe Attaleinae, tribe Cocoseae, subfamily Arecoideae) endemic to the Andes (Dransfield et al., 2008), growing at elevations from 1700 to 3400 m above sea level (Moraes, 1996). It is evolutionarily unique because it diverged from its sister genus *Allagoptera* Nees by vicariance of a widespread South American ancestor likely due to the formation of the aquatic Pebas system, approximately 22 million years ago (Meerow et al., 2015). This ancestral lineage originated in the Atlantic coastal forest of Brazil and spread west into the Andean region 29–22 million years ago (Meerow et al., 2015), a time when the central eastern Andes had not yet reached half of its current elevation (Boschman, 2021; Gregory‐Wodzicki, 2000). Lineages that arrived in the Andes from the Atlantic coastal forest are extremely rare (Antonelli et al., 2018), likely because of the long‐distance dispersal, or the barrier presented by the Cerrado and Chaco dry ecosystems. Unlike many Andean species‐rich clades, there are only three currently recognized species within *Parajubaea* (Dransfield et al., 2008). Whether this is a result of low speciation rates or due to extinction after diversification is unknown. Two of the three species, *P. torallyi* (Mart.) Burret and *P. sunkha* M. Moraes, are allopatric species endemic to Bolivia, and currently found in very few interandean dry valleys with semideciduous forests or xeric vegetation in the south‐central part of the country (Table S1, Figure 1; Moraes, 1996, 2020; Moraes et al., 2020). The third species, *P. cocoides* Burret, is known to exist only in cultivation, usually in plazas and courtyards of many Andean cities in Colombia, Ecuador and Peru (Moraes, 1996; Moraes & Henderson, 1990; but see Roca, 2010). The most recent dated phylogenetic analysis of the tribe Cocoseae (Meerow et al., 2015) sampled a single individual each from *P. cocoides* and *P. torallyi* but did not include *P. sunkha*. Thus, the phylogenetic relationship among the three species remains unknown. Furthermore, genetic data have shown that cultivated and domesticated plants originate from wild populations, and can be subject to a complex array of processes ranging from hybridization among wild species (Fan & Whitaker, 2024; Pérez‐Escobar et al., 2021), reduction of genetic diversity and introgression (Ding et al., 2022) and even the combination of introgressions, hybridizations and polyploidization (Song et al., 2023). DNA sequencing may therefore help to identify the genetic origin of *P. cocoides* as a cultigen.

*Parajubaea* is the source of many non‐timber forest products. The fruit and seeds are edible and are consumed at a local commercial scale in Bolivia (Moraes, 1996; Vargas, 1994). *Parajubaea*'s petiole fiber is used for the manufacturing of mattresses, saddle pillows and ropes. Leaves are harvested for construction, and the crafting of fans and baskets, while palm hearts and young leaves are used for forage (Enssle et al., 2006; Moraes et al., 2017). However, fiber harvest can hinder the recruitment of new individuals because inflorescences and infructescences are commonly removed with this practice (Enssle et al., 2006). The two Bolivian species (*P. sunkha* and *P. torallyi*) are threatened by their unsustainable harvest, forest fragmentation, intensive land use conversion for local crops, limited regeneration in certain areas, their slow growth and the small geographic range of their populations (Enssle et al., 2006; Moraes et al., 2017; Thompson et al., 2009). Consequently, both species are listed as Endangered by the IUCN (Enssle, 2006; Enssle et al., 2006; Moraes, 2022).

In this study, we (1) infer the phylogenetic relationships among the three *Parajubaea* species to gain insight into their evolutionary origin, (2) estimate the genetic diversity and structure of wild Bolivian *Parajubaea* populations, (3) delineate management units for conservation, which are defined as local populations managed as distinct units because of their demographic independence (Hohenlohe et al., 2021) and (4) recommend strategies for long‐term in situ and ex situ conservation of the two wild non‐timber forest species. *Parajubaea cocoides* is a cultigen hypothesized to have originated from wild Bolivian *Parajubaea* given its morphological resemblance with *P. torallyi* and because no wild populations exist in the northern Andes (Moraes & Henderson, 1990). Thus, we expect *P. cocoides* to be nested within the Bolivian *Parajubaea* species in a phylogenetic analysis. We further hypothesize that wild *Parajubaea* populations in Bolivia will show low genetic diversity probably due to genetic drift in the usually small number of individuals in narrow endemic species, and the genetic structure will be shaped by geographic distance as demonstrated for other Neotropical palms due to distance limitations in gene flow (e.g. Melo et al., 2018; Roncal et al., 2015; Sanín et al., 2022).

## 2 MATERIALS AND METHODS

### 2.1 Sample collection and total DNA extraction

To investigate the phylogenetic relationship among the three *Parajubaea* species we sampled 12 individuals of *P. torallyi*, 5 of *P. sunkha* and 6 of *P. cocoides* (Table S2). One individual each from *Allogoptera leucocalyx* (Drude) Kuntze, *A. caudescens* (Mart.) Kuntze and *A. arenaria* (Gomes) Kuntze were sampled as the outgroup. We obtained DNA sequences for five nuclear genes through Sanger sequencing for a total of 26 individuals. To address the second objective of the study, we sampled a total of 194 individuals of *P. sunkha* and *P. torallyi* for genotyping‐by‐sequencing (GBS; Table S3). Three localities in the department of Santa Cruz, Bolivia, were sampled for *P. sunkha* in October 2016, resulting in a total of 48 individuals. Samples of *P. torallyi* were collected at six localities in the departments of Chuquisaca and Potosí, Bolivia, from 146 individuals between October 2016 and August 2020. All collection sites consisted of wild populations, except for Sucre, where *Parajubaea* is cultivated (Figure 1). Fragments of fresh leaves from every individual were stored and transported in paper envelopes containing silica gel. We stored leaf samples at the Herbario Nacional de Bolivia (LPB) and at Memorial University of Newfoundland in Canada.

We ground leaf fragments using a TissueLyser LT machine (Qiagen). We isolated DNA from 50 mg of dried leaf tissue using the Qiagen DNeasy Plant Mini Kit with the following modifications to the original manufacturer's protocol. We added 600 μL of Buffer AP1 to the ground plant material. After vortexing, we incubated the mixture for 15 min at 65°C. We added 195 μL of Buffer P3 to the lysate. We used 50 μL of Qiagen Buffer AE for the final DNA elution. We performed a second elution step by pouring the volume obtained from the first elution directly on the same Qiagen column and waiting for 5 min at room temperature. We diluted total DNA extractions to 20 ng/μL in Qiagen Buffer EB.

### 2.2 Amplification of low‐copy nuclear genes and phylogenetic reconstruction

To address our first objective, we sequenced five low‐copy nuclear DNA regions from the WRKY gene family: WRKY6, WRKY7, WRKY12, WRKY16 and WRKY21 following Meerow et al. (2015). WRKY transcription factors are mostly plant‐specific proteins that were chosen because they proved useful for inferring the species‐level phylogenetic tree of tribe Cocoseae (Meerow et al., 2015). Polymerase chain reaction (PCR) was performed using a 20 μL reaction volume containing 5 μL of the Qiagen AllTaq© 4× Master Mix (final concentration 1×), 0.25 μM of each forward and reverse primer, and 20 ng of template DNA. PCR amplification was carried out in a Veriti 96‐Well Thermal Cycler (Applied Biosystems) with a cycling protocol of 2 min at 95°C (initial denaturation), followed by 40 cycles of 20 s at 95°C (denaturation), 30 s at 56–58°C (annealing) and 30 s at 72°C (extension), followed by a final 5‐min extension at 72°C. WRKY primers, annealing temperatures and PCR product sizes are detailed in Table S4.

Amplifications were visualized by electrophoresis in 1.5% agarose gel stained with RedSafe™ and under ultraviolet light. PCR products were purified using the Qiagen QIAquick PCR Purification Kit (Qiagen Inc, Valencia, CA, USA) before being sent for Sanger sequencing to the Centre for Applied Genomics at the Hospital for Sick Children (Canada, http://www.tcag.ca/). The resulting chromatograms were observed, assembled and edited in *Geneious Prime*® 2023.2.1 (https://www.geneious.com, Biomatters Ltd.). IUPAC codes were used to treat heterozygotes. Sequences for each molecular marker were aligned individually using the Geneious alignment implementation of *Geneious Prime*®. Alignments were later manually curated to get a total concatenated DNA matrix consisting of 3753 aligned positions and approximately 21% missing data.

We used two model‐based methods to reconstruct the phylogenetic relationships of *Parajubaea*. A maximum likelihood (ML) analysis was conducted in *RAxML‐NG* v1.2.0 (Kozlov et al., 2019), and Bayesian inference (BI) was carried out in *MrBayes* v3.2.7 (Ronquist et al., 2012), both available in the *CIPRES Science Gateway* v3.3 (Miller et al., 2010). We determined the best‐fit nucleotide substitution model for each WRKY region with *ModelTest‐NG* v0.1.7 (Darriba et al., 2020) also available in *CIPRES*. We specified a maximum parsimony topology type, selected the candidate model's rate heterogeneity to both +I + G, and the remaining model parameters were set as default. We chose the Akaike and Bayesian information criteria to select the best‐fit model for each WRKY region (Table S4).

For the ML analysis, we set the among‐site rate heterogeneity model to discrete GAMMA with four categories, mean category rates and ML estimate of alpha. We evaluated branch support by conducting Felsenstein bootstrap with 1000 replicates. All other *RAxML‐NG* parameters were set as default. For the BI, we executed two independent Markov chain Monte Carlo (MCMC) runs; each with four simultaneous chains, using a random starting tree and ran for one million generations sampling every 1000 trees. Partitions were set to be independent in terms of the shape of the scaled gamma distribution of site rates, proportion of invariable sites (pinvar), substitution rates matrix (revmat) and stationary nucleotide frequencies (statefreqs). The overall substitution rate was allowed to vary across the subsets of the alignment (ratepr = variable). We assessed the stabilization of each run by looking at the plot of the generation versus log‐likelihood values in *Tracer* v1.7.1 (Rambaut et al., 2018), and by ensuring that all effective sample sizes exceeded 200. We also verified that the standard deviation of split frequencies was close to 0. and that the potential scale reduction factor was close to 1.0 as runs converged. We discarded the first 25% of generations as burn‐in samples. Post burn‐in samples from the two runs were combined and used to calculate a majority rule consensus tree summarizing information on topology, branch lengths and posterior probabilities (PP). We visualized ML and BI trees in *FigTree* v1.4.4 (http://tree.bio.ed.ac.uk/software/figtree/).

### 2.3 Genotyping‐by‐sequencing (GBS)

The Institut de Biologie Intégrative et de Systèmes of the University of Laval in Canada conducted a two‐enzyme GBS (Poland et al., 2012) on the DNA extracted from 194 individuals of *P. sunkha* and *P. torallyi*. We followed Abed et al. (2019) for all steps of DNA digestion with *Sbf*I and *Msp*I restriction enzymes, design and ligation of unique barcodes 10–12 base pairs (bp) long and common/Y adapters (Mascher et al., 2013), PCR amplification and genomic library preparation. We sequenced samples in two separate batches as they became available from the field. For the first, which comprised 168 samples (Table S3), an Ion Torrent Proton technology (ThermoFisher Scientific) and single‐end sequencing were applied. For the second, which comprised 26 samples, we obtained paired‐end reads with an Illumina NovaSeq 6000 S4 machine (Table S3). Génome Québec (Canada) performed all sequencing.

We demultiplexed the raw reads with *Sabre* (https://github.com/najoshi/sabre) and screened them with *FastQC* v0.11.5 (Andrews, 2010) to check for Phred quality scores and to determine the most appropriate parameters for trimming in *Trimmomatic* v0.38 (Bolger et al., 2014). For every read, we removed the first 15 bases and shortened the length to 90 nucleotides. We discarded reads that were shorter than 90 bp, or that had an average quality score lower than 20.

To combine single‐end and paired‐end reads, and in the absence of a reference genome for *Parajubaea*, we used a de novo approach in *Stacks* v2.53 (Rochette et al., 2019). We first created the reverse complement of the reverse reads of all samples that were paired‐end sequenced (Table S3) with the *FASTX toolkit* v0.0.14 (http://hannonlab.cshl.edu/fastx_toolkit/). Forward reads and reverse‐complement reads of each sample were then concatenated into a single file. Following Rochette and Catchen (2017) and Paris et al. (2017), we selected a subset of 20 samples (two from each sample locality) to test for the most appropriate value of *M* (number of mismatches allowed between stacks of reads within individuals) for our dataset in *Stacks*. We then ran the *denovo_map* pipeline in *Stacks* with the complete set of samples with *m* = 3, *M* = 5, *n* = *M*. Lastly, using the *populations* module of *Stacks*, we kept those SNPs that were present in at least two sample localities and at least half of the individuals of those sample localities (*p* = 2 and *r* = 0.5), and no minimum allele frequency threshold (default).

### 2.4 Genetic structure and diversity of Bolivian *Parajubaea*

To investigate the genetic structure among *Parajubaea* individuals we used two approaches. First, we applied a popular Bayesian, model‐based clustering method, *STRUCTURE* v2.3.4. (Falush et al., 2003, 2007; Pritchard et al., 2000). We ran *STRUCTURE* for the two *Parajubaea* species separately and combined. Since *STRUCTURE* can be sensitive to linked loci, we used a complete and a reduced de novo SNP set. The reduced SNP set included only the first SNP of each locus (option –write‐single‐snp in *Stacks*). We determined the number of genetic clusters (*K*) with default parameter settings and correlated allele frequencies. The linkage model proposed by Falush et al. (2003) was not designed to handle linkage disequilibrium between markers that are very tightly linked (Porras‐Hurtado et al., 2013), which is the case of SNPs, thus we used the admixture model in every analysis. Furthermore, since we wanted to compare the results produced with the complete and reduced SNP set, we chose to use the admixture model with both datasets to avoid introducing more variation. We ran analyses without a priori population information. All analyses consisted of 20 iterations for each value of *K*, and of 200,000 Markov Chain Monte Carlo (MCMC) generations after a 100,000‐generation burn‐in for each iteration. For the combined *Parajubaea* analysis we allowed *K* to vary from one (no population structure) to 10 genetic clusters (nine collection sites plus one). To explore genetic structure further, we ran analyses for each species separately, varying *K* from one to seven for *P. torallyi*, and from one to six for *P. sunkha*. We then used *STRUCTURE HARVESTER* v0.6.93 (Earl & vonHoldt, 2012) to collate the *STRUCTURE* results, explore different likelihood values across all *K*s of all iterations, perform the *𝛥K* test proposed by Evanno et al. (2005), and to prepare files for downstream steps. We aligned genetic clusters across iterations with *CLUMPP* v1.1.2 (Jakobsson & Rosenberg, 2007). Lastly, we produced bar plots representing the coefficient of membership of each individual to genetic clusters with *DISTRUCT* v1.1 (Rosenberg, 2004).

Second, using the complete de novo SNP set, we performed a Discriminant Analysis of Principal Components (DAPC) (Jombart et al., 2010) available in the package *adegenet* (Jombart, 2008) in *R* v4.0.3 (R Core Team, 2020), for the two *Parajubaea* species combined and for *P. torallyi* alone, testing the hypothesis that every collection site comprises a separate genetic group. Thus, we tested a *K* of eight for the two species together, and a *K* of five for *P. torallyi*. To optimize the number of principal components to retain, we carried out a cross‐validation test of 200 replicates with the *xvalDapc* function, which calculates the number of principal components attaining the lowest mean squared error. We included all Sucre individuals in *STRUCTURE* but not in the DAPC because we wanted to obtain an assignment score that would reveal the potential origin of those cultivated *P. torallyi* individuals in Bolivia. This DAPC detected a batch effect in our data (see Section 3 ‘Results’). To mitigate this batch effect we used a pseudo‐reference approach for SNP discovery to diminish reference biases that result from shorter single‐end reads (Lou & Therkildsen, 2022). We describe this method in detail in the supporting file.

To test for the effect of missing data on our results, we conducted eight DAPC analyses with four levels of missing data, with and without imputation. To this end, the *R* filter of the *populations* module of *Stacks* was applied to the pseudo reference SNP set to retain those SNPs that were present in at least 20%, 40%, 60% and 80% of the total number of *Parajubaea* samples irrespective of their collection site. Imputation of missing genotypes was carried out in *GenoDive* v.3.06 (Meirmans, 2020) and was based on the overall allele frequencies.

We performed a Mantel test to detect isolation by distance. We used the *R* package *ade4* (Chessel et al., 2004) to measure the correlation between a matrix of pairwise *F'*_*ST*_ values among the nine *Parajubaea* sample localities and their geographic distances. We estimated pairwise Weir and Cockerham's (1984) *F'*_*ST*_ values with the package *hierfstat* v0.5.10 (Goudet, 2005) in *R*. We calculated pairwise geographic distances in kilometers with the Geographic Distance Matrix Generator (Ersts, 2011). The Mantel test used the Pearson coefficient and a default alpha value of 0.05.

Lastly, we reran the *populations* module in *Stacks* on the complete de novo SNP set to compute summary statistics and genetic diversity indexes for each genetic cluster. We therefore assigned individuals to each of the resulting genetic clusters (not sample sites). We kept the requisite for each locus to be present in at least two genetic clusters (*p* = 2), but reduced the proportion of individuals in a cluster that must have a given locus to 10% (*r* = 0.1) to avoid a large reduction in the number of SNPs. We estimated the observed (*H*_*O*_) and expected heterozygosity (*H*_*E*_), nucleotide polymorphism diversity, number of private alleles, and the inbreeding coefficient *F*_IS_. For pairs of genetic clusters, we estimated pairwise *F'*_ST_ values as above. We tested the significance of our calculated pairwise values by comparing them to a null distribution created via 999 random permutations of our groups. Because of their cultivated nature, we decided to eliminate Sucre individuals from estimates of genetic diversity and pairwise *F*_ST_ comparisons of genetic groups. We used this last genetic cluster‐based de novo SNP set (*p* = 2, *r* = 0.1) to conduct an analysis of molecular variance (AMOVA) implemented in *GenoDive* v3.06 to test the distribution of genetic variation within and among genetic clusters, using the infinite allele model and testing its significance with 999 permutations. Table S5 summarizes all SNP sets used in this study and the analyses conducted with each.

## 3 RESULTS

### 3.1 Phylogenetic relationships among the three *Parajubaea* species

**FIGURE 2:**
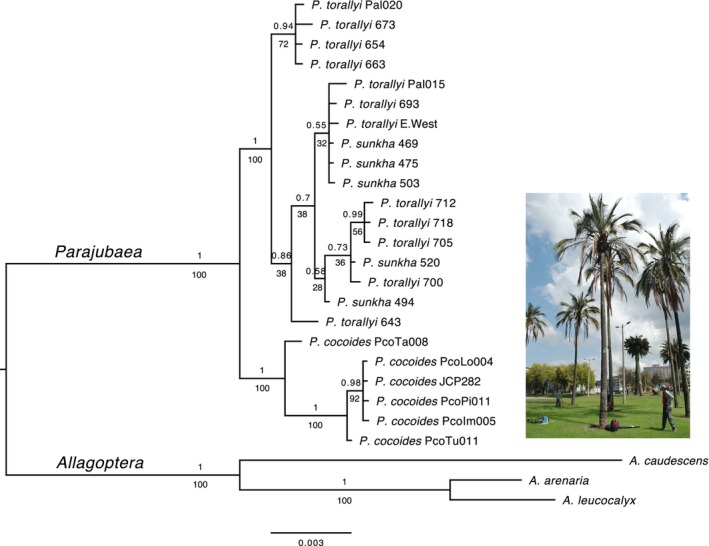
Bayesian 50% majority rule consensus phylogram of 26 *Parajubaea* individuals and outgroup resulting from the analysis of five low‐copy nuclear WRKY loci (3753 bp). Values above the branches are the Bayesian posterior probabilities, and values below are the maximum likelihood bootstrap values. Maximum likelihood topology was identical to the Bayesian tree. Tips show species name and sample locality or number as in Table S2. Scale bar represents estimated substitutions per site. Photo of *P. cocoides* cultivated in Quito, Ecuador.

Both ML and BI concatenated analyses recovered congruent topologies for *Parajubaea* and the outgroup (Figure 2). *Parajubaea* was monophyletic (1.0 PP, 100% bootstrap support (BS)). *Parajubaea cocoides* was monophyletic (1.0 PP, 100% BS) and was sister to a clade formed by all individuals of *P. torallyi* and *P. sunkha*. Contrary to our expectation, *P. cocoides* was not nested within the *P. torallyi/P. sunkha* clade (Figure 2). Interestingly, the individual collected from Tabaconas, Peru (*P. cocoides* PcoTa008), which some argue is a wild population, was sister to all other *P. cocoides*. *Parajubaea torallyi* and *P. sunkha* were each paraphyletic, but together formed a monophyletic group (0.99 PP, 100% BS). Within this *P. torallyi/P. sunkha* clade, there were two sister clades (0.94 PP, 75% BS and 0.86 PP, 38% BS, respectively) whose composition did not correspond to geographic location. In general, individuals from any given sample locality (e.g. Lajas, Palmarcito, Sauce Mayo) did not form a clade. Branch support values within the *P. torallyi/P. sunkha* clade were low to moderate in the ML tree (28%–72% BS) and moderate to high in the BI tree (0.55–0.99 PP).

### 3.2 Genotyping‐by‐sequencing, filtering and SNP discovery

The single‐end GBS rendered close to 353.5 million reads of 25–239 bp. Around 21 million (5.9%) reads of that total did not match any barcode when no mismatches were allowed in *Sabre*. After trimming reads for quality and size, 82.45% of the input reads were retained, corresponding to an average of approximately 1.63 million reads per sample (0.26–4.63 million reads per sample, Figure S1a). The paired‐end GBS yielded over 105.5 million reads (approximately 52.8 million pairs) between 143 and 147 bp in length. Less than 1% of the total reads had no barcode match. When trimming the demultiplexed paired‐end sequences, none were discarded because all reads were longer than 90 bp and their average Phred score was always higher than 20. The paired‐end GBS produced an average of 3.91 million reads (1.95 million pairs of reads) per sample, with a range between 2.34 and 8.37 million reads per sample (Figure S1b). The *Stacks* pipeline allows checking for depth of coverage of each individual at two instances, namely after the *ustacks* and *gstacks* modules. We checked that all samples accomplished a depth of coverage of at least 20× in both instances, therefore all *Parajubaea* samples were kept for genotyping.

The complete de novo SNP set contained 15,134 SNPs, while the reduced SNP set (one SNP per locus) contained 4710 SNPs. A similar proportion of missing data was observed for the complete (90.4%) and the reduced (89.6%) SNP sets. The de novo SNP set obtained after grouping individuals by genetic cluster consisted of 2317 SNP and had 81.3% missing data.

### 3.3 Genetic structure and diversity of Bolivian *Parajubaea*

Comparison of the *STRUCTURE* results between the complete and reduced de novo SNP sets, for both *Parajubaea* species combined and across values of *K*, showed that all *P. sunkha* individuals form a genetic group (red in Figure S2); Ruditayoj, Lajas and Sucre formed another group (green in Figure S2). Differences between these two datasets appeared in the El Palmar site of *P. torallyi*, where a higher level of admixture was observed across values of *K* with the reduced SNP set. Another difference was present in the Sauce Mayo and Palmarcito sites of *P. torallyi*, for which we found a higher level of admixture across values of *K* with the complete SNP set. For *K* of 5, which was the best‐fit model for both species combined (Figure S3a), individuals in Sauce Mayo grouped in the same genetic cluster with El Palmar/Palmarcito in the reduced SNP set, while they appeared highly admixed in the complete SNP set (Figure S2).

**FIGURE 3:**
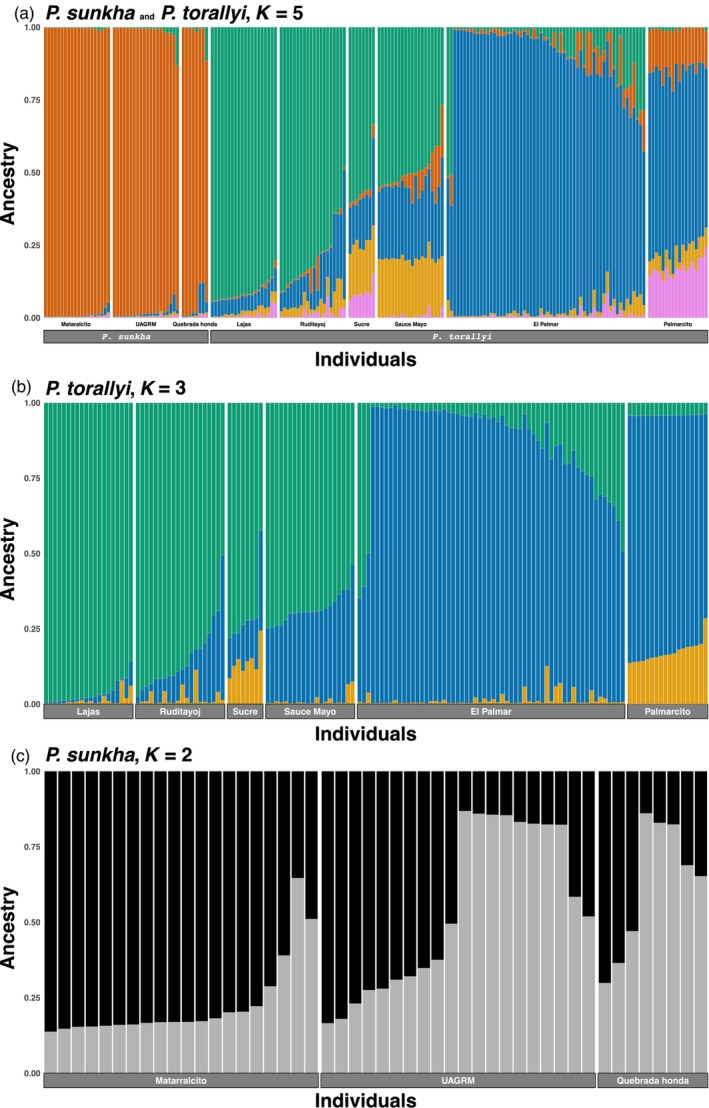
Bar plots showing the genetic structure of both *Parajubaea* species in Bolivia estimated with *STRUCTURE*. Analyses using the complete set of 15,134 de novo SNPs for (a) both species combined and a *K* of 5, (b) for *P. torallyi* and a *K* of 3 and (c) for *P. sunkha* and a *K* of 2. Each vertical bar represents an individual and is colored by the proportion of its assignment to each genetic cluster. Sample localities are indicated at the bottom of the bar plots.

When *P. torallyi* was analyzed alone, Ruditayoj, Lajas and Sucre formed a group (green in Figure S4) across *K* values both with the complete and the reduced SNP sets, as in the combined *STRUCTURE* analysis. Additionally, Sauce Mayo showed up as a distinct group towards the high end of the range of *K* with both sets. El Palmar and Ruditayoj individuals showed higher admixture with the reduced de novo SNP set. For *K* of 3, which was the best‐fit model for *P. torallyi* alone (Figure S3b), individuals in Sauce Mayo grouped in the same genetic cluster with El Palmar/Palmarcito with the reduced SNP set, whereas with the complete SNP set they grouped with Lajas/Ruditayo (Figure S4). When *P. sunkha* was analyzed alone, all individuals were highly admixed across values of *K* with both de novo SNP sets (Figure S5). Since we observed that the significant reduction of SNP data (15,134 vs. 4710 SNPs) prevented a higher differentiation of El Palmar individuals, and because most genetic clusters were the same, we decided to report below only the *STRUCTURE* results on the complete de novo SNP set (Figure 3) and dropped the reduced SNP set from further analyses (DAPC, AMOVA, *F*_ST_; Table S5). In addition, as stated before, the missing data between these two datasets were very similar.

*STRUCTURE* analysis of the two species combined, at a *K* of five (Figure 3a), resulted in all *P. sunkha* individuals being assigned to a single cluster with a score of 80% or higher. We found more admixture in *P. torallyi* with 76 out of 146 individuals (52%) assigned to a single cluster (≥80% score). None of the individuals from Sauce Mayo, Palmarcito or Sucre achieved an assignment score of 80% or above. In each of the rest of *P. torallyi*'s collection sites (El Palmar, Lajas and Ruditayoj) at least 50% of their individuals had an assignment score ≥80%.

When *P. torallyi* was assessed independently (Figure 3b), two major groups were evident, El Palmar and Palmarcito (in blue), which are geographically close to each other, and the remaining *P. torallyi* collection sites (in green, Figure 3b, Figure S4). These two major groups within *P. torallyi* were also revealed in the combined‐species STRUCTURE analysis (Figure 3a). Fifty‐seven per cent of *P. torallyi* individuals were assigned to one genetic cluster with a score ≥80%. Again, all individuals from Sauce Mayo and Sucre were admixed with assignment scores below 80%, but approximately over a third of the individuals of Palmarcito were assigned (score ≥ 80%) to the same group as El Palmar. In the rest of collection sites, most individuals (68% or higher) were assigned to one group. The seed origin of the cultivated individuals in Sucre probably was Sauce Mayo or Ruditayoj or Lajas due to Sucre's genetic assignment similarity with individuals from those collection sites.

In the analysis of *P. sunkha* alone, the log‐likelihood supported *K* of one as the best model, whereas *∆K* achieved its greater value at *K* of two (Figure S3c). However, this last inference must be considered carefully since the Evanno test is not capable of comparing *K* of one to greater values. At *K* of 2, 26 individuals (54%) of the 48 *P. sunkha* samples showed assignment scores to one group. Since the analysis of *P. sunkha* alone showed great admixture among individuals (Figure S5), we treat them as a single genetic group. Altogether, the STRUCTURE analysis revealed three main genetic groups for Bolivian *Parajubaea*: (1) all individuals of *P. sunkha*, (2) *P. torallyi* individuals from El Palmar and Palmarcito and (3) *P. torallyi* individuals from Lajas, Ruditayoj, Sucre and Sauce Mayo.

**FIGURE 4:**
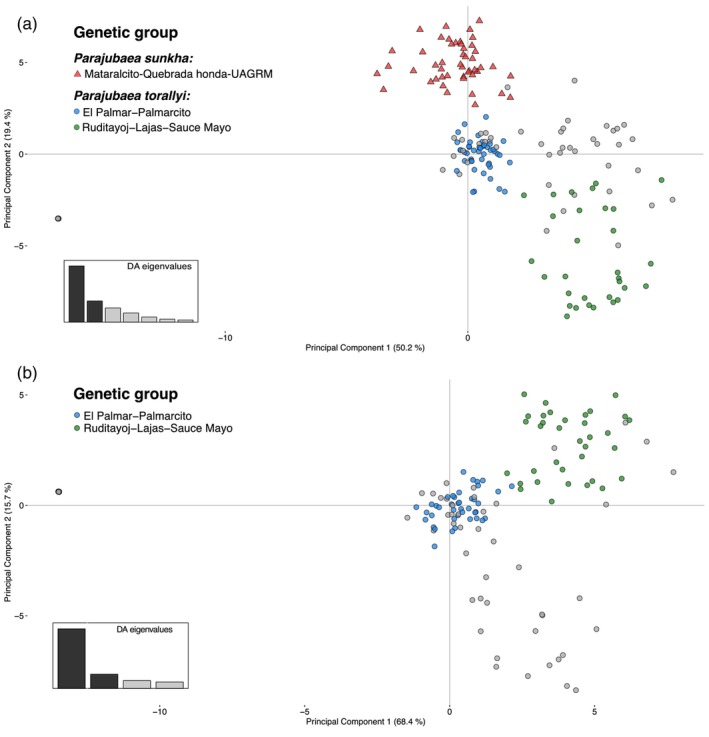
Plots of the discriminant analysis of principal components (DAPC) using the complete set of 15,134 de novo SNPs obtained from genotyping‐by‐sequencing. The percentage of variance explained by each discriminant function appears on the *x* and *y* axes. Individuals are colored according to their genetic group assignment (score ≥ 0.80) from the *STRUCTURE* analysis. Individuals in grey are admixed, attaining an assignment score lower than 0.80 for any of the genetic groups. A) both *Parajubaea* species combined. B) *P. torallyi* alone.

For the DAPC analysis of the two species combined, we retained 61 principal components, as they achieved the lowest mean squared error. The first and second discriminant functions accounted for almost 70% of the total variation. Individuals clustered roughly into three genetic groups as in the STRUCTURE analysis (Figure 4). Admixed individuals in grey (assignment scores < 80%) were mostly from the Sauce Mayo and Palmarcito sites (Figure 4, Figure S6a). The third discriminant function accounted for 13% of the variation and separated Sauce Mayo from the other sample sites (Figure S6b). Sunkha individuals did not segregate by sample site (Figure S6a,b). When evaluating *P. torallyi* alone, the first two discriminant functions explained 84% of the total variation. This ordination distinguished the two *P. torallyi* genetic groups inferred from *STRUCTURE* and also distinguished all *P. torallyi* sample sites except for Ruditayoj and Lajas (Figure S6c,d).

We observed a tight clustering and distance of Palmarcito individuals from the other *P. torallyi* sites revealing a batch effect due to read type (paired‐end vs single‐end). To mitigate this batch effect, we conducted a DAPC using the pseudo reference SNP set, whereby the big separation of Palmarcito individuals was no longer visible and the same three genetic clusters recovered in the de novo DAPC were more clearly separated (Figure 4, Figure S7). Furthermore, the *R* parameter in *Stacks* rendered datasets with different numbers of SNPs and levels of missing data ranging from 2437 SNPs with 15.10% of missing data to 19,087 SNPs with 52.71% missing data (Table S6). Applying imputation of missing genotypes did not alter fundamentally the distribution or grouping of the individuals in the DAPC analysis, being the most notable difference that individuals of Palmarcito were more spread out when imputation of missing data was performed than when it was not. Notably, all eight DAPCs (Figure S7) showed the same results of three genetic clusters towards the edges and the highly admixed individuals of Sauce Mayo and Palmarcito grouped in the center, demonstrating that different levels of missing data do not change our results. Since all the DAPC and *STRUCTURE* results were highly concordant, we are confident about the recognition of the three main genetic clusters.

The Mantel test showed a significant positive correlation between sample locality genetic differentiation and their geographic distances (*R* = 0.349, *p*‐value = 0.025, Table S7), suggesting the presence of isolation by geographic distance. When comparing the genetic groups, Lajas/Ruditayoj/Sauce Mayo showed the most private alleles (768) and the highest nucleotide diversity (0.204), while the Sunkha group showed the least private alleles (143) and lowest nucleotide diversity (0.146, Table 2). *H*_*O*_ and *H*_*E*_ were very close (values overlapped when taking into account the standard error) in the two *P. torallyi* genetic groups, meeting the neutral expectation of allele frequencies consistent with Hardy Weinberg equilibrium (Table 2). In the Sunkha group, however, *H*_*O*_ was higher than *H*_*E*_ which means there was an excess in heterozygosity suggesting an excess in outbreeding or an isolate‐breaking effect (i.e. crossing between genetically differentiated populations). Inbreeding coefficients were positive but close to zero for the two *P. torallyi* genetic groups, meeting the neutral expectation (Table 2). Only Sunkha had a negative *F*_IS_, corroborating the excess in outbreeding in this genetic group. *F*_ST_ values showed low to moderate differentiation between genetic groups suggesting high to moderate gene flow amongst them. The highest *F*_ST_ was between Lajas/Ruditayoj/Sauce Mayo and Sunkha (0.0856), while the lowest *F*_ST_ was between Lajas/Ruditayoj/Sauce Mayo and El Palmar/Palmarcito (0.0761) (Table 3).

**TABLE 2:** Genetic diversity statistics for three *Parajubaea* genetic groups in Bolivia recommended as management units for conservation.

Species/genetic group	№ of individuals	№ of private alleles	Nucleotide diversity π (SE)	Observed heterozygosity *H*_*O*_ (SE)	Expected heterozygosity *H*_*E*_ (SE)	Inbreeding coefficient *F*_IS_ (SE)
*Parajubaea sunkha*						
Sunkha	48	143	0.14577 (0.00485)	0.17513 (0.00749)	0.13707 (0.00456)	−0.03974 (0.17885)
*Parajubaea torallyi*						
El Palmar/Palmarcito	78	438	0.16396 (0.00443)	0.16455 (0.00652)	0.15724 (0.00424)	0.05313 (0.19145)
Ruditayoj/Lajas/Sauce Mayo	60	768	0.20353 (0.00402)	0.19101 (0.00614)	0.19395 (0.00383)	0.09445 (0.18472)

*Note*: Estimates were based on a set of 2317 de novo SNPs obtained through genotyping‐by‐sequencing.

Abbreviation: SE, standard error in parenthesis.

**TABLE 3:** Pairwise *F*_ST_ values among the three genetic groups of *Parajubaea* in Bolivia recommended as management units.

	Sunkha	El Palmar/Palmarcito	Ruditayoj/Lajas/Sauce Mayo
Sunkha	—	0.0812	0.0856
El Palmar/Palmarcito		—	0.0761

*Note*: All *F*_ST_ values are significant, as determined by comparison to a null distribution via 999 random permutations. Estimates were obtained with a set of 2317 de novo SNPs from genotyping‐by‐sequencing.

Analysis of molecular variance using the genetic‐cluster‐based de novo SNP set revealed that the variation within individuals accounted for 86.5%, while the variation among individuals within genetic clusters and among genetic clusters represented 0 and 13.5%, of the total variation, respectively (Table 4). The *F*_ST_ estimate for the differentiation among genetic clusters was 0.164.

**TABLE 4:** Analysis of molecular variance using the genetic‐cluster‐based de novo set of 2317 SNPs with *Stacks* filters of *p* = 2 and *r* = 0.1.

Source of variation	%var	*F*‐stat	*F*‐value	SD	*p*‐value	*F*′‐value
Within individuals	0.865	F_it	0.135	0.022	–	—
Among individuals within genetic clusters	−0.00	F_is	−0.00	0.021	0.554	—
Among genetic clusters	0.135	F_st	0.135	0.010	0.001	0.164

*Note*: Standard deviations of *F*‐statistics were obtained through jackknifing over loci.

## 4 DISCUSSION

Non‐timber forest products are exploited worldwide and are of paramount importance to local economies. Thus, a characterization of the evolutionary history, population structure and genetic diversity of natural populations can assist the conservation and sustainable harvest of such products. Our phylogenetic analysis does not support the hypothesis that *P. cocoides* originated from extant wild Bolivian populations. GBS data support the recognition of a single Bolivian species and the hypothesis of low genetic diversity for narrow endemic species such as *Parajubaea*. We found a genetic structure shaped by distance, but no inbreeding signal which was unexpected. We showed the presence of three genetic clusters which we use to propose three management units, as well as in situ and ex situ conservation strategies.

### 4.1 Taxonomic status and evolutionary relationships

Multiple lines of evidence challenge the current taxonomy of recognizing two morphology‐based Bolivian species; instead, they support a population‐level differentiation within a single species. First, the estimates of *F*_ST_ among genetic clusters are weak and more indicative of intraspecific variation. Second, the number of private alleles for *P. sunkha* is smaller (143) than the number of private alleles for any of the other two genetic clusters in *P. torallyi* (438 and 768). Lastly, the DAPC indicates as much genetic differentiation between the *P. torallyi* sampling sites as observed between the two putative species.

GBS applied to two closely related North American palm genera also rejected the taxonomic recognition of the two morphologically defined *Washingtonia* species, and supported the recognition of two instead of three *Brahea* species (Klimova et al., 2018). GBS was also useful in delimiting species in the Neotropical palm genus *Acrocomia*, reducing the number of species to four (Díaz et al., 2021). Altogether, these results support the hypothesis that many traditional morphological characters used to distinguish palm species (e.g. Table S1) do not have phylogenetic signals or are plastic (Roncal et al., 2012).

The phylogenetic relationships did not support the hypothesis that *P. cocoides* originated from extant wild Bolivian populations, instead, it seems that *P. cocoides* evolved as an independent species that later became cultivated leaving no wild individuals. Also, domesticated crop species like *Dioscorea alata* (yam) have no wild populations, and no clearly identified closest wild relative (Sharif et al., 2020; Table 1). Some genetic studies of domesticated palms have provided insights into their origins and relationships with wild relatives (e.g. Clement et al., 2017; Díaz et al., 2021; Gros‐Balthazard et al., 2017; Gunn et al., 2011; Pérez‐Escobar et al., 2021). For example, the incipiently domesticated *Acrocomia aculeata* used for biodiesel oil has a higher genetic diversity in Brazil, suggesting the species' origin in this area, but has an unknown sister species (Díaz et al., 2021; Table 1). The evolution of *Phoenix dactylifera*'s genome (date palm) was influenced by introgression with two wild relatives (*Phoenix theophrasti* and *P. sylvestris*) approximately 2100 years ago, highlighting the ancient hybrid origin of the date palm (Pérez‐Escobar et al., 2021). The amount of admixture, if any, between *Parajubaea cocoides* and the extant wild Bolivian species remains unknown, and we acknowledge that the use of five nuclear loci may not fully represent the potential evolutionary history of the genus.

### 4.2 Genetic structure and diversity

We found a pattern of genetic structure influenced by distance, which may be explained by the biotic agents involved in the genetic exchange between *Parajubaea* populations. Bees (*Apis mellifera, Trigona* sp.), wasps (*Agelaia multiplicata, Eumenes* sp.) and flies (*Volucella* sp., *Eristalis* sp., *Syrphus* sp.) have been reported to transport pollen of these palms (Guerra et al., 1997; Moraes & Henderson, 1990), but may fail to do so over long distances (Araújo et al., 2004; Beekman & Ratnieks, 2000; Prezoto & Gobbi, 2005). Similarly, rodents have been documented as potential seed dispersers (Vargas, 1994), but it is unlikely for such small mammals to carry the relatively large *Parajubaea* seeds over tens or hundreds of kilometers. The Andean spectacled bear (*Tremarctus ornatus*), a large mammal capable of traversing long distances, has been seen feeding on *P. torallyi* fruits (Anibarro, 1994), but seed dispersal by the Andean bear is anecdotal.

*H*_*O*_ in the three genetic clusters is lower than in other Neotropical palms sources of non‐timber forest products with notably wider distributions like *Euterpe edulis* (0.62–0.79, Gaiotto et al., 2003) and *Oenocarpus bataua* var. *bataua* (0.47–0.88, Escobar et al., 2018). *H*_*O*_ is also lower than in other IUCN vulnerable palms like *Pseudophoenix sargentii* and *P. vinifera* (0.15–0.44 and 0.45–0.55, respectively, Rodríguez‐Peña et al., 2014), and *Ceroxylon quindiuense* (0.436–0.569, Sanín et al., 2017; 0.59–0.62, Chacón‐Vargas et al., 2020). However, *H*_*O*_ for the Bolivian *Parajubaea* palms is close to the lower end of values found in populations of IUCN critically endangered palms with restricted distribution, such as *Pseudophoenix lediniana* (0.25) and *P. ekmanii* (0.20–0.29) (Rodríguez‐Peña et al., 2014), *Coccothrinax jimenezii* (0.294–0.342, Jestrow et al., 2016) and *Tahina spectabilis* (0.01–0.25, Shapcott et al., 2020).

The negative and close‐to‐zero inbreeding coefficients indicated no risk of inbreeding depression. This is uncommon in studies of wild populations of endangered palms (Jestrow et al., 2016; Namoff et al., 2011; Rodríguez‐Peña et al., 2014; Shapcott et al., 2020), and even for palms of no conservation concern (Cibrián‐Jaramillo et al., 2009; Escobar et al., 2018; Gaiotto et al., 2003; Luna et al., 2007), which, with few exceptions, report positive *F*_IS_ values. The avoidance of inbreeding in *Parajubaea* could be explained because these palms are protandrous which prevents self‐fertilization (Moraes & Henderson, 1990), the large suite of pollinators described above could enhance their crossbreeding capacity, and because many wild stand sizes are large consisting of hundreds to thousands of individuals. *Parajubaea*, therefore, is among the endangered plant species (e.g. Dalapicolla et al., 2021; Rodríguez‐Rodríguez et al., 2022), and self‐incompatible dioecious or monoecious plants (e.g. Allendorf & Luikart, 2007; Balloux, 2004) that exhibit heterozygosity excess. We cannot rule out the possibility of local adaptation or drift among populations followed by secondary contact to explain the heterozygosity excess.

### 4.3 Methodological considerations

It is known that GBS can result in a relatively high proportion of missing data (Heffelfinger et al., 2014; Perea et al., 2016; Wallace & Mitchell, 2017), and our study agrees with this tendency. Early simulations and in silico studies cautioned against the potential underestimation of genomic polymorphism due to the presence of missing data caused by allele dropout in reduced representation sequencing (Arnold et al., 2013; Gautier et al., 2012; Luca et al., 2011). Reduced nucleotide diversity (Luca et al., 2011), inflated *H*_*E*_ and *F*_ST_ estimates (Gautier et al., 2012), and other summary statistics were inaccurately calculated from loci with missing data (Arnold et al., 2013). For this reason, many studies discard loci and/or individuals that contain high proportions (>15%) of missing data (e.g. Ackiss et al., 2020; Dupuis et al., 2018; Lah et al., 2016). However, some empirical evidence has demonstrated that allowing for substantial missing data recovers a higher number of loci, and thus more power for population assignment inference than a more conservative approach (Chattopadhyay et al., 2014). Also, different data completion levels showed little impact on most genetic summary statistics (Shafer et al., 2017) and on the identity of genetic groups or subspecies recovered (our study, Featherstone & McGaughran, 2024). Hodel et al. (2017) showed that as missing data increased, so did heterozygosity and *F*_ST_ estimates, albeit in a less pronounced manner than anticipated by simulations. We strived to achieve a compromise between a large number of markers to compensate for the pervasive missing data, and the increased computational demand resulting from the analysis of large datasets. Contrary to Chattopadhyay et al. (2014), who found that large amounts of missing data (90%) obliterated any intermediate ancestry coefficient, our analysis revealed highly admixed individuals despite the high percentage of missing data. In Li and Latch (2021), different percentages of total missing data did not impact genetic structure but non‐random missingness did, whereby samples with high missing data were indistinguishable from truly admixed individuals appearing at the center of a plot. Consistent with expectations, and cross‐validated by the *STRUCTURE* results, we found highly admixed individuals (Sauce Mayo, Palmarcito) at the center of the DAPC plot irrespective of the amount of missing data (from 15% to 53%) (Figure S7).

Another methodological consideration of restriction site‐associated DNA sequencing (RADSeq) protocols like GBS is their limited transferability. Unlike markers that can be transferred across taxa with relative ease such as SSR (e.g. in palms; Billotte et al., 2004) or target‐capture probes (e.g. in palms; de La Harpe et al., 2019), the exact same SNP set obtained through GBS cannot be recovered with another taxon sampling or sequencing batch, particularly when produced de novo. Since RADSeq relies on the conservation of restriction sites among individuals, polymorphism in or the lack of the restriction enzyme cutting site leads to allele dropout (Andrews et al., 2016). Stochastic processes can cause one allele to amplify more than the other allele at a given locus (PCR duplicates, Schweyen et al., 2014). Differential GC content in the restriction fragments causes preferential PCR amplification of loci with high GC content (Benjamini & Speed, 2012). Additionally, the number of loci recovered will depend on the genome size of the taxa involved. All these issues may result in biases in the measurement of genetic variation (Andrews et al., 2016; Davey et al., 2013; Puritz et al., 2014). For these reasons, RADSeq is more appropriate for the intraspecific level or for species of shallow divergence (Harvey et al., 2016). For instance, in a population‐level RADseq study that involved four species of two sister avian genera, only about 0.3%–0.8% of all loci inferred de novo overlapped across all four species (McCormack et al., 2012). Contrastingly, population‐level target‐capture loci attained an average of 92% loci overlap across 40 species of birds spanning 14 families (Harvey et al., 2016). In our GBS study, since *P. sunkha* and *P. torallyi* were found conspecific, we obtained a high percentage of loci overlap (82.4%) between the two when individuals were assigned to their respective genetic clusters.

### 4.4 Conservation units

Among the different types of conservation units in the literature, management units are the most used. Management units are conceived as populations or groups of populations that reflect current demographic independence, regardless of the approach used to delineate them, and they do not necessarily imply phylogenetic divergence (Funk et al., 2012; Palsbøll et al., 2007). Based on our resulting genetic groups, we propose to treat all *P. sunkha* individuals as one management unit and to split *P. torallyi* into two management units: El Palmar/Palmarcito and Lajas/Ruditayoj/Sauce Mayo. These two latter management units represent two metapopulations, one located in the north of the department of Chuquisaca and the other in the center of it and in the eastern part of the Potosí department (Figure 1). These two metapopulations differ in geological conditions but not morphologically. The center has an older geological formation, with extensive Silurian to Devonian rock substrates, and palms are usually found in ravines or in organic matter adjacent to the rocks (Jiménez et al., 2009; Moretti et al., 2002). The metapopulation in the north grows in younger, Triassic, Jurassic and Cretaceous substrates (Carretero et al., 2011).

### 4.5 In situ and ex situ conservation

Actions need to be implemented to ensure the in situ conservation of *Parajubaea* in its natural habitat. For the two *P. torallyi* management units, only the wild stand at El Palmar occurs within the national system of protected areas in Bolivia. Based on our genomic evidence, we have proposed the municipal council of Ckochas to designate a protected area in the Lajas zone. We have also contacted the municipality of El Villar, through the National Herbarium of Bolivia, to create a protected area for Sauce Mayo.

*Parajubaea sunkha* was evaluated in the Red Book of Andean Plants of Bolivia as threatened (EN; Moraes et al., 2012). Harvesting practices are unsustainable for adult individuals and producers are not organized for fibre extraction. There has also been a mix of socioeconomic activities in the same territory and a reduction in the levels of natural regeneration of the Sunkha genetic group (Moraes, 2011). Decades ago, eight confirmed wild stands existed (Vargas, 1994), within an area of occupancy of barely 56 km^2^ (Enssle, 2006). Most of those stands consisted of a few adults, except for Mataralcito, where individuals can be counted by the hundreds, and thus account for about 70% of this particular genetic group (Enssle et al., 2006). There is one in situ protected area for the Sunkha genetic group, the Río Grande Valles Cruceños Integrated Management Natural Area, with 734,000 ha that includes only a fraction of the distribution of this management unit. Therefore, we strongly recommend that the in situ protection of Mataralcito and adjacent stands (Figure 1) must be considered as a priority and urgent. *Parajubaea* is an emblematic palm that appears in the Bolivian coat of arms representing the country's plant diversity; this is an advantage when proposing protected areas to the government.

Article 9 of the Convention on Biological Diversity states the role of ex situ conservation for the purpose of complementing in situ measures (Convention on Biological Diversity (CBD), 1992). Currently, there is fragmented evidence on the real success of ex situ management in preventing species from going extinct, recovering depleted populations or maintaining their viability (Gant et al., 2021). However, in many cases, within the country, ex situ facilities like nurseries contain unique genetic diversity and may represent the best source to provide propagules for species recovery (e.g. Asmussen‐Lange et al., 2011). In 2021, at the National Herbarium of Bolivia, an ex situ germplasm collection of *Parajubaea* was created by installing several germination experiments with hundreds of seeds from El Palmar and Ruditayoj sites, representing two of the three proposed management units. We thus recommend augmenting this ex situ collection with seeds from the Sunkha management unit, following the recommendations of Namoff et al. (2010), who suggested that the optimal sampling for ex situ conservation that captures most allelic variation is 15 individuals per management unit. However, due to the low germination success of palm seeds, we suggest collecting at least double this amount, if possible, in the hundreds as mentioned above. The seedlings will be used for reintroductions or augmentations if wild populations face a rapid decline, thus this ex situ collection will act as an insurance.

## 5 CONCLUSION

The hypothesis that the *P. cocoides* cultigen originated from extant wild Bolivian populations was not supported. Furthermore, our DNA sequencing data challenged the taxonomic recognition of *P. sunkha* and *P. torallyi* as two separate endemic palm species of Bolivia. The low genetic diversity of these emblematic palms is shaped by geographic distance and consists of at least three genetic clusters which we recommend treating as separate management units. Endangered species sources of non‐timber forest products, such as *Parajubaea*, deserve funding priority for extensive genome sequencing that will reveal their intraspecific variation, evolutionary history, and genes under selection, so that we can prevent their extinction in the near future.

## CONFLICT OF INTEREST STATEMENT

The authors declare no conflict of interest to this work.

## Supporting information


Appendix S1.‐S2.

## Data Availability

Data for this study are available from the Dryad Digital Repository: https://doi.org/10.5061/dryad.k0p2ngfgx. Sanger sequences have been submitted to the NCBI website (accession numbers are available in the Appendices S1 and S2 file).

## References

[eva13765-bib-0001] Abed, A. , Légaré, G. , Pomerleau, S. , St‐Cyr, J. , Boyle, B. , & Belzile, F. (2019). Genotyping‐by‐sequencing on the ion torrent platform in barley: Methods and protocols. In W. Hardwood (Ed.), Methods in molecular biology (pp. 233–252). Humana Press. 10.1007/978-1-4939-8944-7_15 30460569

[eva13765-bib-0002] Ackiss, A. S. , Larson, W. A. , & Stott, W. (2020). Genotyping‐by‐sequencing illuminates high levels of divergence among sympatric forms of coregonines in the Laurentian Great Lakes. Evolutionary Applications, 13(5), 1037–1054. 10.1111/eva.12919 32431751 PMC7232772

[eva13765-bib-0003] Allendorf, F. W. , & Luikart, G. (2007). Conservation and the genetics of populations. Blackwell Publishing.

[eva13765-bib-0004] Andrews, K. R. , Good, J. M. , Miller, M. R. , Luikart, G. , & Hohenlohe, P. A. (2016). Harnessing the power of RADseq for ecological and evolutionary genomics. Nature Reviews Genetics, 17, 81–92. 10.1038/nrg.2015.28 PMC4823021 26729255

[eva13765-bib-0005] Andrews, S. (2010). FastQC: a quality control tool for high throughput sequence data . http://www.bioinformatics.babraham.ac.uk/projects/fastqc

[eva13765-bib-0006] Anibarro, G. (1994). Diagnóstico de potencialidades del bosque de palmeras en los cantones Pasopaya y Rodeo del municipio Presto . ACLO‐Chuquisaca, Sucre. Internal report.

[eva13765-bib-0007] Antonelli, A. , Zizka, A. , Antunes Carvalho, F. , Scharn, R. , Bacon, C. D. , Silvestro, D. , & Condamine, F. L. (2018). Amazonia is the primary source of Neotropical biodiversity. Proceedings of the National Academy of Sciences of the United States of America, 115(23), 6034–6039. 10.1073/pnas.1713819115 29760058 PMC6003360

[eva13765-bib-0008] Araújo, E. D. , Costa, M. , Chaud‐Netto, J. , & Fowler, H. G. (2004). Body size and flight distance in stingless bees (Hymenoptera: Meliponini): Inference of flight range and possible ecological implications. Brazilian Journal of Biology, 64(3b), 563–568. 10.1590/S1519-69842004000400003 15619994

[eva13765-bib-0009] Arnau, G. , Bhattacharjee, R. , Sheela, M. N. , Chair, H. , Malapa, R. , Lebot, V. , Abraham, K. , Perrier, X. , Petro, D. , Penet, L. , & Pavis, C. (2017). Understanding the genetic diversity and population structure of yam (*Dioscorea alata* L.) using microsatellite markers. PLoS One, 12(3), e0174150. 10.1371/journal.pone.0174150 28355293 PMC5371318

[eva13765-bib-0010] Arnold, B. , Corbett‐Detig, R. B. , Hartl, D. , & Bomblies, K. (2013). RADseq underestimates diversity and introduces genealogical biases due to nonrandom haplotype sampling. Molecular Ecology, 22(11), 3179–3190. 10.1111/mec.12276 23551379

[eva13765-bib-0011] Asmussen‐Lange, C. B. , Maunder, M. , & Fay, M. F. (2011). Conservation genetics of the critically endangered round Island bottle palm, *Hyophorbe lagenicaulis* (Arecaceae): Can cultivated stocks supplement a residual wild population? Botanical Journal of the Linnean Society, 167, 301–310. 10.1111/j.1095-8339.2011.01175.x

[eva13765-bib-0012] Balloux, F. (2004). Heterozygote excess in small populations and the heterozygote‐excess effective population size. Evolution, 58(9), 1891–1900. 10.1111/j.0014-3820.2004.tb00477.x 15521449

[eva13765-bib-0013] Beekman, M. , & Ratnieks, M. L. W. (2000). Long‐range foraging by the honeybee, *Apis mellifera* L. Functional Ecology, 14(4), 490–496. 10.1046/j.1365-2435.2000.00443.x

[eva13765-bib-0014] Benjamini, Y. , & Speed, T. P. (2012). Summarizing and correcting the GC content bias in high‐throughput sequencing. Nucleic Acids Research, 40(10), e72. 10.1093/nar/gks001 22323520 PMC3378858

[eva13765-bib-0015] Bernal, R. , Torres, C. , García, N. , Isaza, C. , Navarro, J. , Vallejo, M. I. , Galeano, G. , & Balslev, H. (2011). Palm management in South America. The Botanical Review, 77, 607–646. 10.1007/s12229-011-9088-6

[eva13765-bib-0016] Billotte, N. , Marseillac, N. , Brottier, P. , Noyer, J.‐L. , Jacquemoud‐Collet, J.‐P. , Moreau, C. , Couvreur, T. , Chevallier, M.‐H. , Pintaud, J.‐C. , & Risterucci, A.‐M. (2004). Nuclear microsatellite markers for the date palm (*Phoenix dactylifera* L.): Characterization and utility across the genus *Phoenix* and in other palm genera. Molecular Ecology Notes, 4, 256–258. 10.1111/j.1471-8286.2004.00634.x

[eva13765-bib-0017] Bolger, A. M. , Lohse, M. , & Usadel, B. (2014). Trimmomatic: A flexible trimmer for Illumina sequence data. Bioinformatics, 30(15), 2114–2120. 10.1093/bioinformatics/btu170 24695404 PMC4103590

[eva13765-bib-0018] Boschman, L. M. (2021). Andean mountain building since the late cretaceous: A paleoelevation reconstruction. Earth‐Science Reviews, 220, 103640. 10.1016/j.earscirev.2021.103640

[eva13765-bib-0019] Boutet, G. , Carvalho, S. A. , Falque, M. , Peterlongo, P. , Lhuillier, E. , Bouchez, O. , Lavaud, C. , Pilet‐Nayel, M.‐L. , Rivière, N. , & Baranger, A. (2016). SNP discovery and genetic mapping using genotyping by sequencing of whole genome genomic DNA from a pea RIL population. BMC Genomics, 17, 121. 10.1186/s12864-016-2447-2 26892170 PMC4758021

[eva13765-bib-0020] Carretero, A. , Serrano, M. , Borchsenius, F. , & Balslev, H. (2011). Pueblos y plantas de Chuquisaca. Estado del conocimiento de los pueblos, la flora, uso y conservación . Bolivia: Herbario del Sur de Bolivia‐Universidad Mayor, Real y Pontificia de San Francisco Xavier de Chuquisaca.

[eva13765-bib-0021] Chacón‐Vargas, K. , García‐Merchán, V. H. , & Sanín, M. J. (2020). From keystone species to conservation: Conservation genetics of wax palm *Ceroxylon quindiuense* in the largest wild populations of Colombia and selected neighbouring *ex situ* plant collections. Biodiversity and Conservation, 29, 283–302. 10.1007/s10531-019-01882-w

[eva13765-bib-0022] Chattopadhyay, B. , Garg, K. M. , & Ramakrishnan, U. (2014). Effect of diversity and missing data on genetic assignment with RAD‐Seq markers. BMC Research Notes, 7, 841. 10.1186/1756-0500-7-841 25424532 PMC4256836

[eva13765-bib-0023] Chessel, D. , Dufour, A. B. , & Thioulousse, J. (2004). The ade4 package ‐I: One‐table methods. R News, 4, 5–10.

[eva13765-bib-0024] Cibrián‐Jaramillo, A. , Bacon, C. D. , Garwood, N. C. , Bateman, R. M. , Thomas, M. M. , Russell, S. , Bailey, C. D. , Hahn, W. J. , Bridgewater, S. G. M. , & DeSalle, R. (2009). Population genetics of the understory fishtail palm *Chamaedorea ernesti‐augusti* in Belize: High genetic connectivity with local differentiation. BMC Genetics, 10, 65. 10.1186/1471-2156-10-65 19818141 PMC2770526

[eva13765-bib-0025] Clement, C. R. , Cristo‐Araújo, M. , Coppens d'Eeckenbrugge, G. , Reis, V. M. , Lehnebach, R. , & Picanço‐Rodrigues, D. (2017). Origin and dispersal of domesticated peach palm. Frontiers in Ecology and Evolution, 5, 148. 10.3389/fevo.2017.00148

[eva13765-bib-0026] Coates, D. J. , Byrne, M. , & Moritz, C. (2018). Genetic diversity and conservation units: Dealing with the species‐population continuum in the age of genomics. Frontiers in Ecology and Evolution, 6, 165. 10.3389/fevo.2018.00165

[eva13765-bib-0027] Convention on Biological Diversity (CBD) . (1992). Article 9: Ex‐situ conservation. Convention on biological diversity, Rio de Janeiro, Brazil . https://cbd.int/convention/text/default.shtml

[eva13765-bib-0028] Costa, M. F. , Morales‐Marroquín, J. A. , de Araújo Batista, C. E. , Alves‐Pereira, A. , de Almeida Vieira, F. , & Imaculada Zucchi, M. (2022). Population genomics of the Neotropical palm *Copernicia prunifera* (Miller) H. E. Moore: Implications for conservation. PLoS One, 17(11), e0276408. 10.1371/journal.pone.0276408 36327224 PMC9632875

[eva13765-bib-0029] Dalapicolla, J. , Alves, R. , Jaffé, R. , Vasconcelos, S. , Soares Pires, E. , Lopes Nunes, G. , de Souza Pereira, J. B. , Guimarães, J. T. F. , Dias, M. C. , Nogueira Fernandes, T. , Schere, D. , Gomes dos Santos, F. M. , Castilho, A. , Nunes da Fonseca, R. , Esteves, F. A. , Frois Caldeira, C. , & Oliveira, G. (2021). Conservation implications of genetic structure in the narrowest endemic quillwort from the eastern Amazon. Ecology and Evolution, 11(15), 10119–10132. 10.1002/ece3.7812 34367563 PMC8328431

[eva13765-bib-0030] Darriba, D. , Posada, D. , Kozlov, A. M. , Stamatakis, A. , Morel, B. , & Flouri, T. (2020). ModelTest‐NG: A new and scalable tool for the selection of DNA and protein evolutionary models. Molecular Biology and Evolution, 37(1), 291–294. 10.1093/molbev/msz189 31432070 PMC6984357

[eva13765-bib-0031] Davey, J. W. , Cezard, T. , Fuentes‐Utrilla, P. , Eland, C. , Ghrabi, K. , & Blaxter, M. L. (2013). Special features of RAD sequencing data: Implications for genotyping. Molecular Ecology, 22(11), 3151–3164. 10.1111/mec.12084 23110438 PMC3712469

[eva13765-bib-0032] de La Harpe, M. , Hess, J. , Loiseau, O. , Salamin, N. , Lexer, C. , & Paris, M. (2019). A dedicated target capture approach reveals variable genetic markers across micro‐and macro‐evolutionary time scales in palms. Molecular Ecology Resources, 19, 221–234. 10.1111/1755-0998.12945 30240120

[eva13765-bib-0033] Díaz, B. G. , Zucchi, M. I. , Alves‐Pereira, A. , de Almeida, C. P. , Moraes, A. C. L. , Vianna, S. A. , Azevedo‐Filho, J. , & Colombo, C. A. (2021). Genome‐wide SNP analysis to assess the genetic population structure and diversity of *Acrocomia* species. PLoS One, 16(7), e0241025. 10.1371/journal.pone.0241025 34283830 PMC8291712

[eva13765-bib-0034] Ding, Y. M. , Cao, Y. , Zhang, W. P. , Chen, J. , Liu, J. , Li, P. , Renner, S. S. , Zhang, D. , & Bai, W. N. (2022). Population‐genomic analyses reveal bottlenecks and asymmetric introgression from Persian into iron walnut during domestication. Genome Biology, 23(1), 145. 10.1186/s13059-022-02720-z 35787713 PMC9254524

[eva13765-bib-0035] dos Santos, J. R. M. , de Almeida Vieira, F. , Fajardo, C. G. , Brandao, M. M. , Rodrigues Silva, R. A. , & Jump, A. S. (2021). Overexploitation and anthropogenic disturbances threaten the genetic diversity of an economically important Neotropical palm. Biodiversity and Conservation, 30, 2395–2413. 10.1007/s10531-021-02200-z

[eva13765-bib-0036] Dransfield, J. , Uhl, N. W. , Asmussen, C. B. , Baker, W. J. , Harley, M. , & Lewis, C. (2008). Genera Palmarum ‐ The evolution and classification of palms. Royal Botanic Gardens.

[eva13765-bib-0037] Dupuis, J. R. , Oliver, J. C. , Brunet, B. M. T. , Longcore, T. , Johnson, J. J. , & Sperling, F. A. H. (2018). Genomic data indicate ubiquitous evolutionary distinctiveness among populations of California metalmark butterflies. Conservation Genetics, 19, 1097–1108. 10.1007/s10592-018-1081-8

[eva13765-bib-0038] Earl, D. A. , & vonHoldt, B. M. (2012). STRUCTURE HARVESTER: A website and program for visualizing STRUCTURE output and implementing the Evanno method. Conservation Genetics Resources, 4(2), 359–361. 10.1007/s12686-011-9548-7

[eva13765-bib-0039] Enssle, J. (2006). Parajubaea sunkha. The IUCN Red List of Threatened Species 2006, e.T61421A12479273 . 10.2305/IUCN.UK.2006.RLTS.T61421A12479273.en

[eva13765-bib-0040] Enssle, J. , Ferrufino, H. , & Ibisch, P. L. (2006). Conservation status and economic potential of *Parajubaea sunkha*, an endemic palm of Bolivia. Palms, 50(3), 143–151.

[eva13765-bib-0041] Ersts, P. J. (2011). Geographic distance matrix generator v1.2.3 . American Museum of Natural History, Center for Biodiversity and Conservation. http://biodiversityinformatics.amnh.org/open_source/gdmg

[eva13765-bib-0042] Escobar, S. , Pintaud, J.‐C. , Balslev, H. , Bernal, R. , Moraes, M. , Millán, B. , & Montúfar, R. (2018). Genetic structuring in a Neotropical palm analyzed through an Andean orogenesis‐scenario. Ecology and Evolution, 8(16), 8030–8042. 10.1002/ece3.4216 30250682 PMC6144996

[eva13765-bib-0043] Evanno, G. , Regnaut, S. , & Goudet, J. (2005). Detecting the number of clusters of individuals using the software STRUCTURE: A simulation study. Molecular Ecology, 14(8), 2611–2620. 10.1111/j.1365-294X.2005.02553.x 15969739

[eva13765-bib-0044] Falush, D. , Stephens, M. , & Pritchard, J. K. (2003). Inference of population structure using multilocus genotype data: Linked loci and correlated allele frequencies. Genetics, 164(4), 1567–1587. 10.1093/genetics/164.4.1567 12930761 PMC1462648

[eva13765-bib-0045] Falush, D. , Stephens, M. , & Pritchard, J. K. (2007). Inference of population structure using multilocus genotype data: Dominant markers and null alleles. Molecular Ecology Notes, 7(4), 574–578. 10.1111/j.1471-8286.2007.01758.x 18784791 PMC1974779

[eva13765-bib-0046] Fan, Z. , & Whitaker, V. M. (2024). Genomic signatures of strawberry domestication and diversification. The Plant Cell, 36(5), 1622–1636. 10.1093/plcell/koad314 38113879 PMC11062436

[eva13765-bib-0047] Featherstone, L. A. , & McGaughran, A. (2024). The effect of missing data on evolutionary analysis of sequence capture bycatch, with application to an agricultural pest. Molecular Genetics and Genomics, 299, 11. 10.1007/s00438-024-02097-7 38381254 PMC10881687

[eva13765-bib-0048] Funk, W. C. , McKay, J. K. , Hohenlohe, P. A. , & Allendorf, F. W. (2012). Harnessing genomics for delineating conservation units. Trends in Ecology & Evolution, 27(9), 489–496. 10.1016/j.tree.2012.05.012 22727017 PMC4185076

[eva13765-bib-0049] Gaiotto, F. A. , Grattapaglia, D. , & Vencovsky, R. (2003). Genetic structure, mating system, and long‐distance gene flow in heart of palm (*Euterpe edulis* Mart.). Journal of Heredity, 94(5), 399–406. 10.1093/jhered/esg087 14557393

[eva13765-bib-0050] Gant, J. R. , Mair, L. , & McGowan, P. J. K. (2021). Fragmented evidence for the contribution of *ex situ* management to species conservation indicates the need for better reporting. Oryx, 55(4), 573–580. 10.1017/S0030605319000784

[eva13765-bib-0051] Gautier, M. , Gharbi, K. , Cezard, T. , Foucaud, J. , Kerdekhué, C. , Puddle, P. , Cornuet, J.‐M. , & Estoup, A. (2012). The effect of RAD allele dropout on the estimation of genetic variation within and between populations. Molecular Ecology, 22(11), 3165–3178. 10.1111/mec.12089 23110526

[eva13765-bib-0052] Godoy, R. , Overman, H. , Demmer, J. , Apaza, L. , Byron, E. , Huanca, T. , Leonard, W. , Pérez, E. , Reyes‐García, V. , Vadez, V. , Wilkie, D. , Cubas, A. , McSweeney, K. , & Brokaw, N. (2002). Local financial benefits of rain forests: Comparative evidence from amerindian societies in Bolivia and Honduras. Ecological Economics, 40(30), 397–409. 10.1016/S0921-8009(02)00006-X

[eva13765-bib-0053] González‐Pérez, M. A. , Caujapé‐Castells, J. , & Sosa, P. A. (2004). Allozyme variation and structure of the Canarian endemic palm tree *Phoenix canariensis* (Arecaceae): Implications for conservation. Heredity, 93, 307–315. 10.1038/sj.hdy.6800507 15241448

[eva13765-bib-0054] Goudet, J. (2005). HIERFSTAT, a package for R to compute and test hierarchical F‐statistics. Molecular Ecology Notes, 5(1), 184–186. 10.1111/j.1471-8286.2004.00828.x

[eva13765-bib-0055] Gregory‐Wodzicki, K. M. (2000). Uplift history of the central and northern Andes: A review. GSA Bulletin, 112(7), 1091–1105. 10.1130/0016-7606(2000)112<1091:UHOTCA>2.0.CO;2

[eva13765-bib-0056] Gros‐Balthazard, M. , Galimberti, M. , Kousathanas, A. , Newton, C. , Ivorra, S. , Paradis, L. , Vigouroux, Y. , Carter, R. , Tengberg, M. , Battesti, V. , Santoni, S. , Falquet, L. , Pintaud, J.‐C. , Terral, J.‐F. , & Wegmann, D. (2017). The discovery of wild date palms in Oman reveals a complex domestication history involving centres in the Middle East and Africa. Current Biology, 27(14), 2211–2218. e8. 10.1016/j.cub.2017.06.045 28712568

[eva13765-bib-0057] Guerra, J. F. , Ananibar, H. , Ayzama, S. , Cazón, Y. , Cortéz, R. , Fezzio, G. , Martínez, O. , Ovando, A. , Pérez, E. , & Torrico, G. (1997). Biodiversidad de los bosques de El Palmar . Provincia Zudáñez (Chuquisaca). Programa de Bosques Nativos Andinos, La Paz.

[eva13765-bib-0058] Gunn, B. F. , Baudouin, L. , & Olsen, K. M. (2011). Independent origins of cultivated coconut (*Cocos nucifera* L.) in the old world tropics. PLoS One, 6(6), e21143. 10.1371/journal.pone.0021143 21731660 PMC3120816

[eva13765-bib-0059] Hamon, P. , Grover, C. E. , Davis, A. P. , Rakotomalala, J.‐J. , Raharimalala, N. E. , Albert, V. A. , Sreenath, H. L. , Stoffelen, P. , Mitchell, S. E. , Couturon, E. , Hamon, S. , de Kochko, A. , Crouzillat, D. , Rigoreau, M. , Sumirat, U. , Akaffou, S. , & Guyot, R. (2017). Genotyping‐by‐sequencing provides the first well‐resolved phylogeny for coffee (*Coffea*) and insights into the evolution of caffeine content in its species. Molecular Phylogenetics and Evolution, 109, 351–361. 10.1016/j.ympev.2017.02.009 28212875

[eva13765-bib-0060] Harvey, M. G. , Smith, B. T. , Glenn, T. C. , Faircloth, B. C. , & Brumfield, R. T. (2016). Sequence capture versus restriction site associated DNA sequencing for shallow systematics. Systematic Biology, 65(5), 910–924. 10.1093/sysbio/syw036 27288477

[eva13765-bib-0061] Heffelfinger, C. , Fragoso, C. A. , Moreno, M. A. , Overton, J. D. , Mottinger, J. P. , Zhao, H. , Tohme, J. , & Dellaporta, S. L. (2014). Flexible and scalable genotyping‐by‐sequencing strategies for population studies. BMC Genomics, 15, 979. 10.1186/1471-2164-15-979 25406744 PMC4253001

[eva13765-bib-0062] Helyar, S. J. , Hemmer‐Hansen, J. , Bekkevold, D. , Taylor, M. , Ogden, R. , Limborg, M. , Cariani, A. , Maes, G. E. , Diopere, E. , Carvalho, G. R. , & Nielsen, E. E. (2011). Application of SNPs for population genetics of nonmodel organisms: New opportunities and challenges. Molecular Ecology Resources, 11(s1), 123–136. 10.1111/j.1755-0998.2010.02943.x 21429169

[eva13765-bib-0063] Heubach, K. , Wittig, R. , Nuppenau, E.‐A. , & Hahn, K. (2011). The economic importance of non‐timber forest products (NTFPs) for livelihood maintenance of rural west African communities: A case study from northern Benin. Ecological Economics, 70(11), 1991–2001. 10.1016/j.ecolecon.2011.05.015

[eva13765-bib-0064] Hodel, R. G. , Chen, S. , Payton, A. C. , McDaniel, S. F. , Soltis, P. , & Soltis, D. E. (2017). Adding loci improves phytogeographic resolution in red mangroves despite increased missing data: Comparing micro satellites and RAD‐Seq and investigating loci filtering. Nature Scientific Reports, 7, 17598. 10.1038/s41598-017-16810-7 PMC5730610 29242627

[eva13765-bib-0065] Hohenlohe, P. A. , Funk, W. C. , & Rajora, O. P. (2021). Population genomics for wildlife conservation and management. Molecular Ecology, 30(1), 62–82. 10.1111/mec.15720 33145846 PMC7894518

[eva13765-bib-0066] Huang, L. , Wang, X. , Dong, Y. , Long, Y. , Hao, C. , Yan, L. , & Shi, T. (2020). Resequencing 93 accessions of coffee unveils independent and parallel selection during *Coffea* species divergence. Plant Molecular Biology, 103, 51–61. 10.1007/s11103-020-00974-4 32072392

[eva13765-bib-0067] Hurley, P. , & Emery, M. R. (2018). Locating provisioning ecosystem services in urban forests: Forageable woody species in new York City, USA. Landscape and Urban Planning, 170, 266–275. 10.1016/j.landurbplan.2017.09.025

[eva13765-bib-0068] Jakobsson, M. , & Rosenberg, N. A. (2007). CLUMPP: A cluster matching and permutation program for dealing with label switching and multimodality in analysis of population structure. Bioinformatics, 23(14), 1801–1806. 10.1093/bioinformatics/btm233 17485429

[eva13765-bib-0069] Jestrow, B. , Peguero, B. , Jiménez, F. , Cinea, W. , Hass, M. , Reeve, A. , Meerow, A. W. , Griffith, M. P. , Maunder, M. , & Francisco‐Ortega, J. (2016). Genetic diversity and differentiation of the critically endangered hispaniolan palm *Coccothrinax jimenezii* M.M. Mejía & R.G. García based on novel SSR markers. Biochemical Systematics and Ecology, 66, 216–223. 10.1016/j.bse.2016.04.013

[eva13765-bib-0070] Jiménez, N. , López‐Velásquez, S. , & Santiváñez, R. (2009). Evolución tectonomagmática de los Andes bolivianos. Revista de la Asociación Geológica Argentina, 65(1), 36–67.

[eva13765-bib-0071] Jombart, T. (2008). Adegenet: A R package for the multivariate analysis of genetic markers. Bioinformatics, 24(11), 1403–1405. 10.1093/bioinformatics/btn129 18397895

[eva13765-bib-0072] Jombart, T. , Devillard, S. , & Balloux, F. (2010). Discriminant analysis of principal components: A new method for the analysis of genetically structured populations. BMC Genetics, 11, 94. 10.1186/1471-2156-11-94 20950446 PMC2973851

[eva13765-bib-0073] Klimova, A. , Ortega‐Rubio, A. , Vendrami, D. L. J. , & Hoffman, J. I. (2018). Genotyping by sequencing reveals contrasting patterns of population structure, ecologically mediated divergence, and long‐distance dispersal in north American palms. Ecology and Evolution, 8, 5873–5890. 10.1002/ece3.4125 29938100 PMC6010798

[eva13765-bib-0074] Kozlov, A. M. , Darriba, D. , Flouri, T. , Morel, B. , & Stamatakis, A. (2019). RAxML‐NG: A fast, scalable and user‐friendly tool for maximum likelihood phylogenetic inference. Bioinformatics, 35(21), 4453–4455. 10.1093/bioinformatics/btz305 31070718 PMC6821337

[eva13765-bib-0075] Lah, L. , Trense, D. , Benke, H. , Berggren, P. , Gunnlaugsson, Þ. , Lockyer, C. , Öztürk, A. , Öztürk, B. , Pawliczka, I. , Roos, A. , Siebert, U. , Skóra, K. , Víkingsson, G. , & Tiedemann, R. (2016). Spatially explicit analysis of genome‐wide SNPs detects subtle population structure in a mobile marine mammal, the harbor porpoise. PLoS One, 11(10), e0162792. 10.1371/journal.pone.0162792 27783621 PMC5082642

[eva13765-bib-0076] Lanes, E. C. M. , Motoike, S. Y. , Kuki, K. N. , Nick, C. , & Freitas, R. D. (2015). Molecular characterization and population structure of the macaw palm, *Acrocomia aculeata* (Arecaceae), *ex situ* germplasm collection using microsatellites markers. Journal of Heredity, 106(1), 102–112. 10.1093/jhered/esu073 25425677

[eva13765-bib-0077] Li, X. , & Latch, E. K. (2021). Nonrandom missing data can bias principal component analysis inference of population genetic structure. Molecular Ecology Resources, 00, 1–10. 10.1111/1755-0998.13498 34463035

[eva13765-bib-0078] Lou, R. N. , & Therkildsen, N. O. (2022). Batch effects in population genomic studies with low‐coverage whole genome sequencing data: Causes, detection and mitigation. Molecular Ecology Resources, 22, 1678–1692. 10.1111/1755-0998.13559 34825778

[eva13765-bib-0079] Luca, F. , Hudson, R. R. , Witonsky, D. B. , & Di Renzo, A. (2011). A reduced representation approach to population genetic analyses and application to human evolution. Genome Research, 21(7), 1087–1098. 10.1101/gr.119792.110 21628451 PMC3129251

[eva13765-bib-0080] Luna, R. , Epperson, B. K. , & Oyama, K. (2007). High levels of genetic variability and inbreeding in two Neotropical dioecious palms with contrasting life histories. Heredity, 99, 466–476. 10.1038/sj.hdy.6801027 17637694

[eva13765-bib-0081] Macía, M. J. , Armesilla, P. J. , Cámara‐Leret, R. , Paniagua‐Zambrana, N. , Villalba, S. , Balslev, H. , & Pardo‐de‐Santayana, M. (2011). Palm uses in northwestern South America: A quantitative review. The Botanical Review, 77, 462–570. 10.1007/s12229-011-9086-8

[eva13765-bib-0082] Mascher, M. , Wu, S. , Amand, P. , Stein, N. , & Poland, J. (2013). Application of genotyping‐by‐sequencing on semiconductor sequencing platforms: A comparison of genetic and reference‐based marker ordering in barley. PLoS One, 8(10), e76925. 10.1371/journal.pone.0076925 24098570 PMC3789676

[eva13765-bib-0083] McCormack, J. E. , Maley, J. M. , Hird, S. M. , Derryberry, E. P. , Graves, G. R. , & Brumfield, R. T. (2012). Next‐generation sequencing reveals phylogeographic structure and a species tree for recent bird divergences. Molecular Phylogenetics and Evolution, 62, 397–406. 10.1016/j.ympev.2011.10.012 22063264

[eva13765-bib-0084] Meerow, A. W. , Noblick, L. , Salas‐Leiva, D. E. , Sanchez, V. , Francisco‐Ortega, J. , Jestrow, B. , & Nakamura, K. (2015). Phylogeny and historical biogeography of the cocosoid palms (Arecaceae, Arecoideae, Cocoseae) inferred from sequences of six WRKY gene family loci. Cladistics, 31(5), 509–534. 10.1111/cla.12100 34772273

[eva13765-bib-0085] Meirmans, P. G. (2020). Genodive version 3.0: Easy‐to‐use software for the analysis of genetic data of diploids and polyploids. Molecular Ecology Resources, 20, 1126–1131. 10.1111/1755-0998.13145 32061017 PMC7496249

[eva13765-bib-0086] Melo, W. A. , Freitas, C. G. , Bacon, C. D. , & Collevati, R. G. (2018). The road to evolutionary success: Insights from the demographic history of an Amazonian palm. Heredity, 121, 183–195. 10.1038/s41437-018-0074-1 29588509 PMC6039527

[eva13765-bib-0087] Miller, M. A. , Pfeiffer, W. , & Schwartz, T. (2010). Creating the CIPRES science gateway for inference of large phylogenetic trees . Proceedings of the gateway computing environments workshop, 1–8, New Orleans, LA.

[eva13765-bib-0088] Moraes, R. M. (1996). Novelties of the genera *Parajubaea* and *Syagrus* (Palmae) from inter Andean valleys of Bolivia. Novon, 6(1), 85–92. 10.2307/3392218

[eva13765-bib-0089] Moraes, R. M. (2011). Diagnóstico y plan de manejo de la palmera sunkha (Parajubaea sunkha Moraes) de Vallegrande, Santa Cruz,Bolivia. Fundación Natura Bolivia.

[eva13765-bib-0090] Moraes, R. M. (2020). Flora de palmeras de Bolivia (2nd ed.). Herbario Nacional de Bolivia.

[eva13765-bib-0091] Moraes, R. M. (2022). *Parajubaea torallyi. The IUCN red list of threatened species 2022*: e.T38626A212047377. 10.2305/IUCN.UK.2022-2.RLTS.T38626A212047377.en

[eva13765-bib-0092] Moraes, R. M. , de la Barra, N. , & Cuba, I. (2012). Parajubaea sunkha M. Moraes. In Libro Rojo de la Flora amenazada de Bolivia. Vol. I. Zona Andina (pp. 521–523). Ministerio de Medio Ambiente y Agua.

[eva13765-bib-0093] Moraes, R. M. , & Henderson, A. (1990). The genus *Parajubaea* (Palmae). Brittonia, 42(2), 92–99. 10.2307/2807619

[eva13765-bib-0094] Moraes, R. M. , Hurtado, R. , Vargas, C. I. , Vargas, E. V. , & Toledo, G. (2020). Palmeras útiles y endémicas de valles interandinos de Bolivia: especies de *Parajubaea* . In R. M. Moraes (Ed.), Palmeras y Usos: Especies de Bolivia y la Región (pp. 99–110). Herbario Nacional de Bolivia.

[eva13765-bib-0095] Moraes, R. M. , Paniagua‐Zambrana, N. , Cámara‐Leret, R. , Balslev, H. , & Macía, M. J. (2015). Palmas útiles de Bolivia, Colombia, Ecuador y Perú. In H. Balslev , M. J. Macía , & H. Navarrete (Eds.), Cosecha de palmas en el noroeste de Suramérica: bases científicas para su manejo y conservación (pp. 87–102). Pontificia Universidad Católica del Ecuador.

[eva13765-bib-0096] Moraes, R. M. , Roncal, J. , & Hurtado, R. (2017). Uses of *Parajubaea torallyi*, a vulnerable palm of Bolivia. Palms, 61(2), 91–101.

[eva13765-bib-0097] Moretti, I. , Labaume, P. , Sheppard, S. M. F. , & Boulègue, J. (2002). Compartmentalisation of fluid migration pathways in the sub‐Andean zone, Bolivia. Tectonophysics, 348(1–3), 5–24. 10.1016/S0040-1951(01)00246-3

[eva13765-bib-0098] Namoff, S. , Husby, C. E. , Francisco‐Ortega, J. , Noblick, L. R. , Lewis, C. E. , & Griffith, M. P. (2010). How well does a botanical garden collection of a rare palm capture the genetic variation in a wild population? Biological Conservation, 143(5), 1110–1117. 10.1016/j.biocon.2010.02.004

[eva13765-bib-0099] Namoff, S. , Veloz, A. , Jiménez, F. , Rodríguez‐Peña, R. A. , Peguero, B. , Lewis, C. , Moynihan, J. , Abdo, M. , Maunder, M. , von Wettberg, E. , Meerow, A. W. , Griffith, P. , & Francisco‐Ortega, J. (2011). Sweet drinks are made of this: Conservation genetics of an endemic palm species from The Dominican Republic. Journal of Heredity, 102(1), 1–10. 10.1093/jhered/esq118 21172825

[eva13765-bib-0100] Olivares, I. , Tusso, S. , Sanín, M. J. , de La Harpe, M. L. , Loiseau, O. , Rolland, J. , Salamin, N. , Kessler, M. , Shimizu, K. K. , & Paris, M. (2024). Hyper‐cryptic radiation of a tropical montane plant lineage. Molecular Phylogenetics and Evolution, 190, 107954. 10.1016/j.ympev.2023.107954 37898295

[eva13765-bib-0101] Palsbøll, P. J. , Bérubé, M. , & Allendorf, F. W. (2007). Identification of management units using population genetic data. Trends in Ecology & Evolution, 22(1), 11–16. 10.1016/j.tree.2006.09.003 16982114

[eva13765-bib-0102] Paniagua‐Zambrana, N. Y. , Byg, A. , Svenning, J.‐C. , Moraes, R. M. , Grandez, C. , & Balslev, H. (2007). Diversity of palm uses in the western Amazon. Biodiversity and Conservation, 16, 2771–2787. 10.1007/s10531-007-9218-y

[eva13765-bib-0103] Paris, J. R. , Stevens, J. R. , & Catchen, J. M. (2017). Lost in parameter space: A road map for STACKS. Methods in Ecology and Evolution, 8(10), 1360–1373. 10.1111/2041-210X.12775

[eva13765-bib-0104] Perea, C. , De la Hoz, J. F. , Cruz, D. F. , Lobaton, J. D. , Izquierdo, P. , Quintero, J. C. , Raatz, B. , & Duitama, J. (2016). Bioinformatic analysis of genotype by sequencing (GBS) data with NGSEP. BMC Genomics, 17(Supp 5), 498. 10.1186/s12864-016-2827-7 27585926 PMC5009557

[eva13765-bib-0105] Peres, C. A. (2000). Identifying keystone plant resources in tropical forests: The case of gums from *Parkia* pods. Journal of Tropical Ecology, 16, 287–317. 10.1017/S0266467400001413

[eva13765-bib-0106] Pérez‐Escobar, O. A. , Bellot, S. , Przelomska, N. A. S. , Flowers, J. M. , Nesbitt, M. , Ryan, P. , Gutaker, R. M. , Gros‐Balthazard, M. , Wells, T. , Kuhnhäuser, B. G. , Schley, R. , Bogarín, D. , Dodsworth, S. , Diaz, R. , Lehmann, M. , Petoe, P. , Eiserhardt, W. L. , Preick, M. , Hofreiter, M. , … Baker, W. J. (2021). Molecular clocks and archeogenomics of a late period Egyptian date palm leaf reveal introgression from wild relatives and add timestamps on the domestication. Molecular Biology and Evolution, 38(10), 4475–4492. 10.1093/molbev/msab188 34191029 PMC8476131

[eva13765-bib-0107] Poland, J. A. , Brown, P. J. , Sorrells, M. E. , & Jannink, J.‐L. (2012). Development of high‐density genetic maps for barley and wheat using a novel two‐enzyme genotyping‐by‐sequencing approach. PLoS One, 7(2), e32253. 10.1371/journal.pone.0032253 22389690 PMC3289635

[eva13765-bib-0108] Porras‐Hurtado, L. , Ruiz, Y. , Santos, C. , Phillips, C. , Carracedo, Á. , & Lareu, M. V. (2013). An overview of STRUCTURE: Applications, parameter settings, and supporting software. Frontiers in Genetics, 4, 98. 10.3389/fgene.2013.00098 23755071 PMC3665925

[eva13765-bib-0109] Prezoto, F. , & Gobbi, N. (2005). Flight range extension in *Polistes simillimus* Zikán, 1951 (Hymenoptera, Vespidae). Brazilian Archives of Biology and Technology, 48(6), 947–950. 10.1590/S1516-89132005000800011

[eva13765-bib-0110] Pritchard, J. K. , Stephens, M. , & Donnelly, P. (2000). Inference of population structure using multilocus genotype data: Dominant markers and null alleles. Genetics, 155(2), 945–959. 10.1093/genetics/155.2.945 10835412 PMC1461096

[eva13765-bib-0111] Puritz, J. B. , Matz, M. V. , Toonen, R. J. , Weber, J. N. , Bolnick, D. I. , & Bird, C. E. (2014). Demystifying the RAD fad. Molecular Ecology, 23(24), 5937–5942. 10.1111/mec.12965 25319241

[eva13765-bib-0112] R Core Team . (2020). R: A language and environment for statistical computing. R Foundation for Statistical Computing. https://www.R‐project.org/

[eva13765-bib-0113] Rajesh, M. K. , Gangurde, S. S. , Pandey, M. K. , Niral, V. , Sudha, R. , Jerard, B. A. , Kadke, G. N. , Sabana, A. A. , Muralikrishna, K. S. , Samsudeen, K. , Karun, A. , & Prasad, T. S. K. (2021). Insights on genetic diversity, population structure, and linkage disequilibrium in globally diverse coconut accessions using genotyping‐by‐sequencing. Omics: A Journal of Integrative Biology, 25(12), 796–809. 10.1089/omi.2021.0159 34757849

[eva13765-bib-0114] Rambaut, A. , Drummond, A. J. , Xie, D. , Baele, G. , & Suchard, M. A. (2018). Posterior summarisation in Bayesian phylogenetics using tracer 1.7. Systematic Biology, 67(5), 901–904. 10.1093/sysbio/syy032 29718447 PMC6101584

[eva13765-bib-0115] Roca, F. (2010). *Parajubaea cocoides*, a new record for Peru. Palms, 54(3), 133–136.

[eva13765-bib-0116] Rochette, N. C. , & Catchen, J. M. (2017). Deriving genotypes from RAD‐seq short‐read data using STACKS. Nature Protocols, 12, 2640–2659. 10.1038/nprot.2017.123 29189774

[eva13765-bib-0117] Rochette, N. C. , Rivera‐Colón, A. G. , & Catchen, J. M. (2019). Stacks 2: Analytical methods for paired‐end sequencing improve RADseq‐based population genomics. Molecular Ecology, 28(21), 4737–4754. 10.1111/mec.15253 31550391

[eva13765-bib-0118] Rodríguez‐Peña, R. A. , Jestrow, B. , Meerow, A. W. , Clase, T. , Jiménez‐Rodríguez, F. , Griffith, M. P. , Santiago‐Valentín, E. , Sustache‐Sustache, J. A. , & Francisco‐Ortega, J. (2014). Genetic diversity and differentiation of *Pseudophoenix* (Arecaceae) in Hispaniola. Botanical Journal of the Linnean Society, 176(4), 469–485. 10.1111/boj.12223

[eva13765-bib-0119] Rodríguez‐Rodríguez, P. , Fernández De Castro, A. G. , Pérez de Paz, P. L. , Curbelo, L. , Palomares, A. , Mesa, R. , Acevedo, A. , & Sosa, P. A. (2022). Evolution and conservation genetics of an insular hemiparasitic plant lineage at the limit of survival: The case of *Thesium* sect. *Kunkeliella* in the Canary Islands. American Journal of Botany, 109(3), 419–436. 10.1002/ajb2.1830 35289923 PMC9415105

[eva13765-bib-0120] Roncal, J. , Couderc, M. , Baby, P. , Kahn, F. , Millán, B. , Meerow, A. W. , & Pintaud, J.‐C. (2015). Palm diversification in two geologically contrasting regions of western Amazonia. Journal of Biogeography, 42(8), 1503–1513. 10.1111/jbi.12518

[eva13765-bib-0121] Roncal, J. , Henderson, A. , Borchsenius, F. , Sodre Cardoso, S. R. , & Balslev, H. (2012). Can phylogenetic signal, character displacement, or random phenotypic drift explain the morphological variation in the genus *Geonoma* (Arecaceae)? Biological Journal of the Linnean Society, 106(3), 528–539. 10.1111/j.1095-8312.2012.01879.x

[eva13765-bib-0122] Ronquist, F. , Teslenko, M. , van der Mark, P. , Ayres, D. L. , Darling, A. , Höhna, S. , Larget, B. , Liu, L. , Suchard, M. A. , & Huelsenbeck, J. P. (2012). MRBAYES 3.2: Efficient Bayesian phylogenetic inference and model selection across a large model space. Systematic Biology, 61(3), 539–542. 10.1093/sysbio/sys029 22357727 PMC3329765

[eva13765-bib-0123] Rosenberg, N. A. (2004). DISTRUCT: A program for the graphical display of population structure. Molecular Ecology Notes, 4(1), 137–138. 10.1046/j.1471-8286.2003.00566.x

[eva13765-bib-0124] Sanín, M. J. , Cardona, A. , Valencia‐Montoya, W. A. , Torres Jiménez, M. F. , Carvalho‐Madrigal, S. , Gómez, A. C. , Bacon, C. D. , Roquemen Tangarife, T. , Jaramillo, J. S. , Zapata, S. , Valencia, V. , Arboleda Valencia, J. W. , Vargas, V. , & Paris, M. (2022). Volcanic events coincide with plant dispersal across the northern Andes. Global and Planetary Change, 210, 103757. 10.1016/j.gloplacha.2022.103757

[eva13765-bib-0125] Sanín, M. J. , Zapata, P. , Pintaud, J.‐C. , Galeano, G. , Bohórquez, A. , Tohme, J. , & Møller Hansen, M. (2017). Up and down the blind alley: Population divergence with scant gene flow in an endangered tropical lineage of Andean palms (*Ceroxylon quindiuense* clade: Ceroxyloideae). Journal of Heredity, 108(3), 288–298. 10.1093/jhered/esx006 28186241

[eva13765-bib-0126] Schweyen, H. , Rozenberg, A. , & Leese, F. (2014). Detection and removal of PCR duplicates in population genomic ddRAD studies by addition of a degenerate base region (dbr) in sequencing adapters. The Biological Bulletin, 227(2), 146–160. 10.1086/BBLv227n2p146 25411373

[eva13765-bib-0127] Shackelton, C. M. , & Pandey, A. K. (2014). Positioning non‐timber forest products on the development agenda. Forest Policy and Economics, 38, 1–7. 10.1016/j.forpol.2013.07.004

[eva13765-bib-0128] Shackleton, C. M. , Ticktin, T. , & Cunningham, A. B. (2018). Nontimber forest products as ecological and biocultural keystone species. Ecology and Society, 23(4), 22. 10.5751/ES-10469-230422

[eva13765-bib-0129] Shafer, A. B. A. , Peart, C. R. , Tusso, S. , Maayan, I. , Brelsford, A. , Wheat, C. W. , & Wolf, J. B. W. (2017). Bioinformatic processing of RAD‐seq data dramatically impacts downstream population genetic inference. Methods in Ecology and Evolution, 8, 907–917. 10.1111/2041-210X.12700

[eva13765-bib-0130] Shapcott, A. , James, H. , Simmons, L. , Shimizu, Y. , Gardiner, L. , Rabehevitra, D. , Letsara, R. , Cable, S. , Dransfield, J. , Baker, W. J. , & Rakotoarinivo, M. (2020). Population modelling and genetics of a critically endangered Madagascan palm *Tahina spectabilis* . Ecology and Evolution, 10(6), 3120–3137. 10.1002/ece3.6137 32211182 PMC7083664

[eva13765-bib-0131] Shapcott, A. , Rakotoarinivo, M. , Smith, R. J. , Lysaková, G. , Fay, M. F. , & Dransfield, J. (2007). Can we bring Madagascar's critically endangered palms back from the brink? Genetics, ecology and conservation of the critically endangered palm *Beccariophoenix madagascariensis* . Botanical Journal of the Linnean Society, 154, 589–608. 10.1111/j.1095-8339.2007.00676.x

[eva13765-bib-0132] Sharif, B. M. , Burgarella, C. , Cormier, F. , Mournet, P. , Causse, S. , Van, K. N. , Kaoh, J. , Rajaonah, M. T. , Lakshan, S. R. , Waki, J. , Bhattacharjee, R. , Badara, G. , Pachakkil, B. , Arnau, G. , & Chaïr, H. (2020). Genome‐wide genotyping elucidates the geographical diversification and dispersal of the polyploid and clonally propagated yam (*Dioscorea alata*). Annals of Botany, 126(6), 1029–1038. 10.1093/aob/mcaa122 32592585 PMC7596366

[eva13765-bib-0133] Song, A. , Su, J. , Wang, H. , Zhang, Z. , Zhang, X. , Van de Peer, Y. , Chen, F. , Fang, W. , Guan, Z. , Zhang, F. , Wang, Z. , Wang, L. , Ding, B. , Zhao, S. , Ding, L. , Liu, Y. , Zhou, L. , He, J. , Jia, D. , … Chen, F. (2023). Analyses of a chromosome‐scale genome assembly reveal the origin and evolution of cultivated chrysanthemum. Nature Communications, 14(1), 2021. 10.1038/s41467-023-37730-3 PMC10085997 37037808

[eva13765-bib-0134] Terborgh, J. (1986). Keystone plant resources in the tropical forest. In I. Soulé & E. Michael (Eds.), Conservation biology (pp. 330–344). Sinauer.

[eva13765-bib-0135] Thompson, L. N. , Moraes, R. M. , & Baudoin, M. (2009). Estructura poblacional de la palmera endémica *Parajubaea torallyi* (Mart.) Burret en zonas aprovechadas del área natural de manejo integrado El Palmar (Chuquisaca, Bolivia). Ecología en Bolivia, 44, 17–35.

[eva13765-bib-0136] Vargas, C. I. (1994). The ecology and uses of *Parajubaea torallyi* in Bolivia. Principes, 38(3), 146–152.

[eva13765-bib-0137] Wahlén, C. B. (2017). Opportunities for making visible the invisible: Towards an improved understanding of the economic contributions of NTFPs. Forest Policy and Economics, 84, 11–19. 10.1016/j.forpol.2017.04.006

[eva13765-bib-0138] Wallace, J. G. , & Mitchell, S. E. (2017). Genotyping‐by‐sequencing. Current Protocols in Plant Biology, 2, 64–77. 10.1002/cppb.20042 31725977

[eva13765-bib-0139] Weir, B. S. , & Cockerham, C. C. (1984). Estimating *F*‐statistics for the analysis of population structure. Evolution, 38(6), 1358–1370. 10.1111/j.1558-5646.1984.tb05657.x 28563791

[eva13765-bib-0140] Xia, W. , Luo, T. , Zhang, W. , Mason, A. S. , Huang, D. , Huang, X. , Tang, W. , Dou, Y. , Zhang, C. , & Xiao, Y. (2019). Development of high‐density SNP markers and their application in evaluating genetic diversity and population structure in *Elaeis guineensis* . Frontiers in Plant Science, 10, 130. 10.3389/fpls.2019.00130 30809240 PMC6380268

