# Supplementary material for: Genotyping‐by‐sequencing informs conservation of Andean palms sources of non‐timber forest products
Source: Evol Appl. 2024 Jul 31;17(8):e13765. doi: 10.1111/eva.13765 (PMC11291087; doi:10.1111/eva.13765)
Supplement: Supplementary file 1 — Appendix S1.‐S2. [file EVA-17-e13765-s001.docx]

**Supplementary File**

**Genotyping-by-sequencing informs conservation of Andean palms sources of non-timber forest products**

**Appendix S1 – Supplementary Methods include:**

- Methods to obtain a pseudo reference SNP set.
- **Table S1.** Distinguishing morphological and habitat characteristics of species in the genus *Parajubaea*
- **Table S2.** Taxonomic sampling of the genus *Parajubaea* and outgroup *Allagoptera* species used in phylogenetic reconstruction
- **Table S3**. Sample information of *Parajubaea* palms in Bolivia used for genotyping-by-sequencing
- **Table S4.** List of five WRKY genes: primers, annealing temperature, and best-fit nucleotide substitution model for each
- **Table S5.** Overview of SNP datasets and analyses conducted with each

**Appendix S2 – Supplementary Results include:**

- **Table S6.** Interaction between changes in the *R* parameter of *Stacks* and the number of loci, SNPs and % of missing data using the pseudo reference SNP set.
- **Table S7.** Matrices used in the Mantel test of isolation by distance.
- **Figure S1**. Total number of reads per individual demultiplexed with *Sabre* for A) single-end genotyping-by-sequencing, and B) pair-end genotyping-by-sequencing.
- **Figure S2**. Comparison of bar plots showing the genetic structure of both *Parajubaea* species in Bolivia using all *de novo* SNPs and one SNP per locus for *K*=2 to *K*=7
- **Figure S3.** Likelihood and Evanno test of the clustering models evaluated in *STRUCTURE* A) for both species combined, B) for *P. torallyi* only, and C) for *P. sunkha* only**.**
- **Figure S4.** Comparison of bar plots showing the genetic structure of *Parajubaea torallyi* in Bolivia using the complete (left) and reduced (right) *de novo* SNP set for *K*=2 to *K*=7
- **Figure S5.** Comparison of bar plots showing the genetic structure of *Parajubaea sunkha* in Bolivia using the complete (left) and reduced (right) *de novo* SNP set for *K*=2 to *K*=6
- **Figure S6.** Visualisation of the discriminant analysis of principal components (DAPC) based on the complete *de novo* SNP set. Individuals are coloured by collection site (panels A, B and C) or according to their genetic group assignment (score ≥ 0.80) from the *STRUCTURE* analysis (panel D). A) Analysis with both *Parajubaea* species combined showing the first and second discriminant functions. B) Same analysis as in (A) but showing the second and third discriminant functions. C) Analysis for *P. torallyi* alone showing the first and second discriminant functions. D) Same analysis as in (C) but showing the genetic group each individual was assigned to.
- **Figure S7.** Visualisation of the discriminant analysis of principal components (DAPC) based on the pseudo reference SNP set with four different values of the *R* parameter in *Stacks* (20, 40, 60, and 80) with and without imputation
- **References**

**Appendix S1 – Supplementary Methods**

**Pseudo reference SNP set**

The application of SNP filters to eliminate loci associated with a batch effect have been proposed for whole genome sequencing studies which can also be applied to genotyping by sequencing (e.g. Lou & Therkildsen, 2022). To mitigate the batch effect due to different read types (paired-end versus single-end), as in our case study, Lou and Therkildsen (2022) suggest the elimination of private alleles linked to the batch effect grouping, or to use a pseudo reference approach for SNP discovery to diminish reference biases that result from shorter single-end reads. We used a custom script to eliminate the private SNPs associated with samples with pair-end reads (Palmarcito and Sucre). With this dataset, we conducted a DAPC which while diminishing the separation of Palmarcito with respect to the remaining collection sites, it still showed a batch effect. We therefore explored the pseudo reference approach.

The pseudo reference approach used 35 individuals that were paired-end (PE) sequenced to build a ‘pseudo-genome’ that would serve as a reference onto which the reads of all samples (SE and PE) would be mapped. The group of 35 individuals comprised nine samples of *P. cocoides* (sequenced for another study), all *P. torallyi* individuals from Palmarcito (18) and Sucre (8) populations. We used the *denovo_map* pipeline in *Stacks* with default parameters (*m* = 3; *M* = 2; *n* = 1), to produce a catalog of consensus loci among the 35 samples. We mapped the reads of the 194 *Parajubaea* individuals to this catalog of loci with the aid of *Bowtie2* v2.4.1 (Langmead & Salzberg, 2012). We then loaded the resulting *.bam* files into the *ref_map* pipeline of *Stacks* to output the pseudo reference SNP set. We filtered this SNP set with the *populations* module of *Stacks* to retain those markers that were present in at least 20%, 40%, 60% and 80% of the total number of *Parajubaea* samples irrespective of their collection site (R=20, 40, 60, 80).

**Table S1.** Distinguishing morphological and habitat characteristics of species in the genus *Parajubaea* and their distribution in the Neotropics. Table adapted from Borchsenius et al., 1998; Moraes & Henderson, 1990; and Moraes, 2020.

|  | *P. cocoides* | *P. torallyi* | *P. sunkha* |
| --- | --- | --- | --- |
| Distribution | Cultivated in Andean cities of Ecuador, Colombia and Peru. | Endemic, wild in the departments of Chuquisaca and Potosi, Bolivia. | Endemic, wild in the department of Santa Cruz, Bolivia. |
| Habitat | Cities and towns in Andean mountains and valleys, and along roads. | Narrow interandean valleys and open areas, rocky soils and medium semi-deciduous forests | Wide interandean valleys, rich soils and low semi-deciduous forests; mixed with local crops. |
| Elevation | 2500-3000m. | 2000-3400m. | 1700-2200m. |
| Leaves | 20-30 leaves spreading, the lower ones pendulous; pinnae regularly arranged. | 15-40 leaves that are erect but distally curved; pinnae regularly arranged. | 18-26 leaves that are 2-3m long, erect and arching; pinnae irregularly arranged. |
| Stem | 16m tall, 20-30cm diameter, wider at the base; smooth and brown. | 10-26m tall, 25-55cm diameter; rough and dark brown. | 4-10m tall, 25-35cm diameter, covered with old sheaths below the leaf crown. |
| Inflorescence | Staminate flowers are sessile and orange coloured. Pistillate flowers are sessile and purple/violet. | Staminate pedicellate flowers are inserted along the rachillae; 1-2 pistillate flowers per rachilla. | Staminate pedicellate flowers along the rachillae; 7-9 pistillate flowers per rachilla. |
| Fruit and seeds | Fruits 4-5.5cm long x 3-4cm wide; edible seeds are single and 1.4cm in diameter. | Fruits* 2.5-5 x 2.5 cm (microcarpa) and 6-8.5cm long x 4-5.2cm; seeds come in 1(-3), edible, 2 x 1.5cm. | Fruits 3-5 cm long x 2.5-3cm wide; seeds can be 1(-2), edible, 2 x 1.5cm. |

* Smaller fruits in the south of Chuquisaca (Ruditayoj, Lajas) and larger in El Palmar in the north

**Table S2.** Taxonomic sampling of the genus *Parajubaea* and outgroup *Allagoptera* species used in phylogenetic reconstruction. Sample locality, voucher specimens (herbarium acronym) and GenBank sequence accession numbers are given for each palm sampled. Individuals collected in the same locality share GPS coordinates and herbarium voucher. An * preceding a GenBank number are sequences we downloaded from the web.

| **Species and sample number** | **Sample locality** | **Coordinates and elevation** | **Voucher (herbarium)** | **Locus** | | | | |
| --- | --- | --- | --- | --- | --- | --- | --- | --- |
|  |  |  |  | WRKY6 | WRKY7 | WRKY12 | WRKY16 | WRKY21 |
| **Outgroup** |  |  |  |  |  |  |  |  |
| *Allagoptera arenaria* (Gomes) Kuntze | Unknown (from Meerow et al. 2015) |  | Noblick 5168 (IPA) | FJ9577069* | FJ957143* | FJ957216* | FJ957284* | FJ956997* |
| *A. leucocalyx* (Drude) Kuntze | Unknown (from Meerow et al. 2015) |  | Zardini 54930 (FTG) | FJ957071* | FJ957145* | FJ957218* | FJ957286* | FJ956998* |
| *A. caudescens* (Mart.) Kuntze | Unknown (from Meerow et al. 2015) |  | Noblick 5135 (FTG) | FJ957118* | FJ957191* | FJ957260* | FJ957330* | FJ957044* |
| **Ingroup** |  |  |  |  |  |  |  |  |
| *Parajubaea cocoides* Burret | Unknown (from Meerow et al. 2015) |  | J.C.Pintaud 282 (MPU) | FJ957116* | FJ957189* | FJ957257* | FJ957327* | FJ957042* |
| *P. cocoides* PcoTu011 | Ambato, Tungurahua, Ecuador | 1°13'39.0"S 78°37'20.9"W;2,530 m | - | PP755153 | PP764841 | PP779086 | PP792986 | PP795751 |
| *P. cocoides* PcoTa008 | Tabaconas, San Ignacio, Cajamarca, Peru | 5°18'59.8"S 79°17'06.9"W; 1,884 m | Peñafiel 2019-02 (HUTI) | PP755154 | PP764840 | PP779089 | PP792985 | PP795750 |
| *P. cocoides* PcoPi011 | San José de Puembo, Pichincha, Ecuador | 0°11'23.9"S 78°21'11.3"W; 2,516 m. | - | — | — | PP779088 | PP792984 | PP795753 |
| *P. cocoides* PcoIm005 | Hacienda Pinsaquí, Imbabura, Ecuador | 0°16'47.6"N 78°14'22.6"W 2,533 m | - | — | — | PP779087 | PP792983 | PP795752 |
| *P. cocoides* PcoLo004 | Theodoro Wolf, Loja, Ecuador | 4°01'33.3"S 79°12'14.6"W 2,112 m | Peñafiel 2019-01 (HUTI) | PP755155 | PP764839 | — | PP792982 | PP795749 |
| *Parajubaea sunkha* M.Moraes 469 | Mataralcito, Vallegrande, Santa Cruz, Bolivia | 18°53'16" S 64°36'11" W; 1,556 m | M.Moraes Ramirez 2545 (LPB) | PP755142 | PP764842 | PP779091 | PP792987 | PP795734 |
| *P. sunkha* 475 | Mataralcito, Vallegrande, Santa Cruz, Bolivia |  |  | PP755145 | PP764843 | PP779092 | PP792988 | PP795735 |
| *P. sunkha* 494 | UAGRM, Vallegrande, Santa Cruz, Bolivia | 18°30'31.5"S 64°06'03.8"W; 2,044 m | Vargas 101  (USZ) | PP755143 | PP764844 | PP779093 | PP792989 | PP795736 |
| *P. sunkha* 503 | UAGRM, Vallegrande, Santa Cruz, Bolivia |  |  | — | — | PP779094 | PP792990 | PP795737 |
| *P. sunkha* 520 | Quebrada honda, Vallegrande, Santa Cruz, Bolivia | 18°25'44.5"S 64°07'38.6"W | - | PP755144 | PP764845 | PP779090 | PP792991 | PP795738 |
| *Parajubaea torallyi* (Mart.) Burret | Unknown (from Meerow et al. 2015) |  | E. West s.n. | FJ957117* | FJ957190* | FJ957258* | FJ957328* | FJ957043* |
| *P. torallyi* Pal015 | Palmarcito, Zudáñez, Chuquisaca, Bolivia | 18°34'34.9''S 64°52'33.3'' W; 2,700 m | - | PP755151 | PP764836 | PP779103 | PP792997 | — |
| *P. torallyi* Pal020 | Palmarcito, Zudáñez, Chuquisaca, Bolivia |  |  | PP755152 | PP764837 | PP779104 | PP792993 | PP795748 |
| *P. torallyi* 643 | Ruditayoj, Tomina, Chuquisaca, Bolivia | 19°32'59.8"S 64°44'21.9"W; 3,104 m | M.Moraes Ramirez 2567 (LPB) | — | — | PP779096 | PP792996 | PP795739 |
| *P. torallyi* 654 | Ruditayoj, Tomina, Chuquisaca, Bolivia |  |  | PP755147 | — | PP779101 | — | PP795742 |
| *P. torallyi* 693 | Sauce Mayo, Zudáñez, Chuquisaca, Bolivia | 19°36'58.0"S 64°12'56.6"W; 2,842 m | M.Moraes Ramirez 2566 (LPB) | PP755148 | — | PP779098 | — | PP795744 |
| *P. torallyi* 700 | Sauce Mayo, Zudáñez, Chuquisaca, Bolivia |  |  | — | — | — | PP792994 | PP795741 |
| *P. torallyi* 705 | Sauce Mayo, Zudáñez, Chuquisaca, Bolivia |  |  | — | — | PP779099 | PP792992 | PP795745 |
| *P. torallyi* 712 | Sauce Mayo, Zudáñez, Chuquisaca, Bolivia |  |  | PP755149 | PP764838 | — | — | PP795746 |
| *P. torallyi* 718 | Sauce Mayo, Zudáñez, Chuquisaca, Bolivia |  |  | — | — | PP779102 | PP792995 | PP795747 |
| *P. torallyi* 663 | Lajas, Linares, Potosí, Bolivia | 19°34'21.6"S 64°53'53.4"W; 2,925 m | M.Moraes Ramirez 2560 & 2561 (LPB) | PP755146 | — | PP779095 | — | PP795743 |
| *P. torallyi* 673 | Lajas, Linares, Potosí, Bolivia |  |  | PP755150 | — | PP779097 | — | PP795740 |

**Table S3**. Sample collection information of *Parajubaea* palms *in Bolivia* used for genotyping by sequencing. All populations are wild except for the one in Sucre city which is cultivated*.* Voucher specimens deposited at Herbario Nacional de Bolivia in La Paz (LPB), Herbario del Oriente Boliviano in Santa Cruz (USZ), and Herbario del Sur de Bolivia in Sucre (HSB)

| Sample locality | Species/Locality  Acronym | Department, Province | GPS coordinates and elevation | Number of samples | Sequencing type^1^ | Herbarium voucher |
| --- | --- | --- | --- | --- | --- | --- |
| ***Parajubaea sunkha*** | **Ps** |  |  |  |  |  |
| Mataralcito | Mat | Santa Cruz, Vallegrande | 18°53'16" S 64°36'11" W; 1,556 m | 20 | SE | MMR 2545 (LPB) |
| La granja de los valles cruceños. Universidad Autónoma Gabriel René Moreno | UAGRM | Santa Cruz, Vallegrande | 18°30'31.5"S 64°06'03.8"W; 2,044 m | 20 | SE | Vargas 101  (USZ) |
| Quebrada honda | Qh | Santa Cruz, Vallegrande | 18°25'44.5"S 64°07'38.6"W | 8 | SE | - |
| Total *P. sunkha* |  |  |  | 48 |  |  |
| ***Parajubaea torallyi*** | **Pt** |  |  |  |  |  |
| ANMI El Palmar | EP | Chuquisaca, Zudáñez | 18°41'44.1"S 64°55'41.0"W; 2,936 m | 60 | SE | MMR 2546 & 2547 (LPB) |
| Ruditayoj | Ru | Chuquisaca, Tomina | 19°32'59.8"S 64°44'21.9"W; 3,104 m | 20 | SE | MMR 2567 (LPB) |
| Lajas | La | Potosí, Linares | 19°34'21.6"S 64°53'53.4"W; 2,925 m | 20 | SE | MMR 2560 & 2561 (LPB) |
| Sauce Mayo | SM | Chuquisaca, Zudáñez | 19°36'58.0"S 64°12'56.6"W; 2,842 m | 20 | SE | MMR 2566 (LPB) |
| Palmarcito | Pal | Chuquisaca, Zudáñez | 18°34'34.9''S 64°52'33.3'' W; 2,700 m | 18 | PE | - |
| Sucre | Su | Chuquisaca, Oropeza | 19°03'09.0"S 65°16'05.1"W; 2,820 m | 8 | PE | Carretero 98 (HSB) |
| Total *P. torallyi* |  |  |  | 146 |  |  |
| Total # samples |  |  |  | 194 |  |  |

^1^ Refers to the type of sequencing used for a group of samples: SE = single-end; PE = paired-end. Refer to the Methods section for details.

**Table S4**. Low-copy nuclear DNA regions in the WRKY gene family used to infer the phylogenetic relationships among *Parajubaea* species. For the Bayesian inference we used the GTR+I+G4 model for the WRKY16 and WRKY21 regions because their best-fit nucleotide substitution models are not supported in MrBayes. Primer sequences and names as in Meerow et al. (2015). Primers were used for both amplification and sequencing.

| Low-copy nuclear gene | Primers 5’-3’ | Annealing temperature | Sequence length and position in concatenation | Nucleotide substitution model |
| --- | --- | --- | --- | --- |
| WRKY6 | F1 CCAAACCCAAGGTAGGTTTCAGC  R1 CCTAACAGGGCACCCAGCATT | 58 ̊ C | 329-745;  1-747 | HKY+I+G4 |
| WRKY7 | F2 ACCCAAAGCCTCCACACA  R2 TCACCGCCCTTGGATCAT | 56 ̊ C | 732-746;  748-1497 | K80+I+G4 |
| WRKY12 | F2 GGGTGCTCACAACCACTCCA  R3 TGCCCTCTCCACATGCTTTC | 58 ̊ C | 119-831;  1498-2330 | HKY+I+G4 |
| WRKY16 | F1 AGCCGTCAAAAACAGCCCATT  R1 CAAAGCAGCCACCGAGTTACA | 56 ̊ C | 109-539;  2331-2882 | TPM3uf+I+G4  GTR+I+G4 (MrBayes) |
| WRKY21 | F1 AGAGTAACCCATGCCCACGA  R1 GCACACCTCTGCACCTGAAA | 58 ̊ C | 308-833  2883-3753 | TPM1uf+I+G4  GTR+I+G4 (MrBayes) |

**Table S5.** Overview of SNP datasets and analyses conducted with each. Parameter values in *Stacks* are specified for *p*, *r* and *R*

| Collection-site-based  De novo complete SNP set p=2, r=50  15,134 SNPs  90.4% missing data | Collection-site-based  De novo reduced SNP set p=2, r=50  4,710 SNPs  89.6% missing data | Genetic-cluster-based  De novo complete SNP set  p=2, r=10  2,317 SNPs  81.3% missing data | Collection-site-based  Pseudo reference SNP set  R=20; 40; 60; 80  SNPs and missing data in Table S6 |
| --- | --- | --- | --- |
| STRUCTURE | STRUCTURE | - | - |
| DAPC | - | - | DAPC |
| - | - | Diversity indexes and pairwise Fst | - |
| - | - | AMOVA | - |

**Appendix S2 – Supplementary Results**

**Table S6.** Interaction between changes in the *R* parameter of *Stacks* and the number of loci, SNPs and % of missing data using the pseudo reference SNP set.

| Value of *R* | % of missing data | Variants | |
| --- | --- | --- | --- |
|  |  | Loci | SNPs |
| 20 | 52.71 | 12087 | 19087 |
| 40 | 34.93 | 6250 | 9668 |
| 60 | 23.60 | 4261 | 5612 |
| 80 | 15.10 | 2803 | 2437 |

**Table S7**. Matrices used in the Mantel test of genetic isolation by distance. Above the diagonal are pairwise *F_ST_* values among nine sampled *Parajubaea* localities in Bolivia. Below the diagonal are distances in kilometres among the *Parajubaea* localities obtained from Geographic Distance Matrix Generator (Ersts, 2011).

|  |  | ***Parajubaea sunkha*** | | | ***Parajubaea torallyi*** | | | | | |
| --- | --- | --- | --- | --- | --- | --- | --- | --- | --- | --- |
|  |  | **Mataralcito** | **UAGRM** | **Quebrada honda** | **El Palmar** | **Ruditayoj** | **Lajas** | **Sauce Mayo** | **Palmarcito** | **Sucre** |
|  | **Mataralcito** | - | 0.0204 | 0.0165 | 0.1126 | 0.1403 | 0.1589 | 0.1753 | 0.0027 | 0.0036 |
| ***P. sunkha*** | **UAGRM** | 67.69 | - | 0.0016 | 0.0775 | 0.1393 | 0.1735 | 0.1718 | 0.0021 | 0.0039 |
|  | **Quebrada honda** | 71.59 | 9.30 | - | 0.0846 | 0.1036 | 0.1145 | 0.0933 | 0.0029 | 0.0044 |
|  | **El Palmar** | 40.38 | 89.70 | 89.55 | - | 0.0376 | 0.0735 | 0.0472 | 0.0005 | 0.0007 |
|  | **Ruditayoj** | 75.09 | 133.97 | 140.43 | 97.15 | - | 0.0308 | 0.0851 | 0.0022 | 0.0009 |
| ***P. torallyi*** | **Lajas** | 82.31 | 145.13 | 150.96 | 97.69 | 16.84 | - | 0.1452 | 0.0024 | 0.0017 |
|  | **Sauce Mayo** | 90.72 | 123.86 | 132.47 | 126.93 | 55.42 | 71.73 | - | 0.0027 | 0.0030 |
|  | **Palmarcito** | 45.05 | 82.12 | 80.70 | 14.37 | 109.33 | 110.93 | 134.98 | - | 0.1731 |
|  | **Sucre** | 72.37 | 137.08 | 138.84 | 53.49 | 78.43 | 69.74 | 127.10 | 67.21 | - |

**Figure S1.** Total number of reads per individual demultiplexed with *Sabre* for A) single-end genotyping-by-sequencing, and B) pair-end genotyping-by-sequencing.

A)


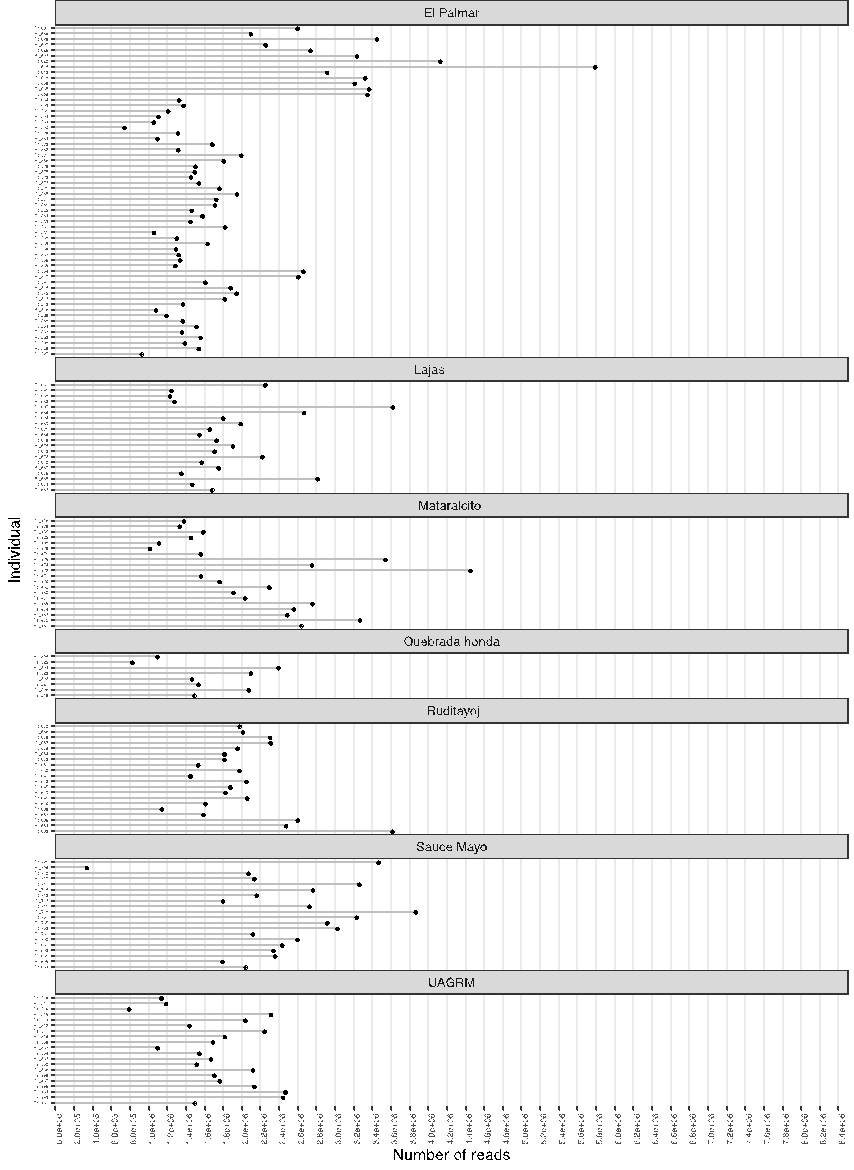


B)

**
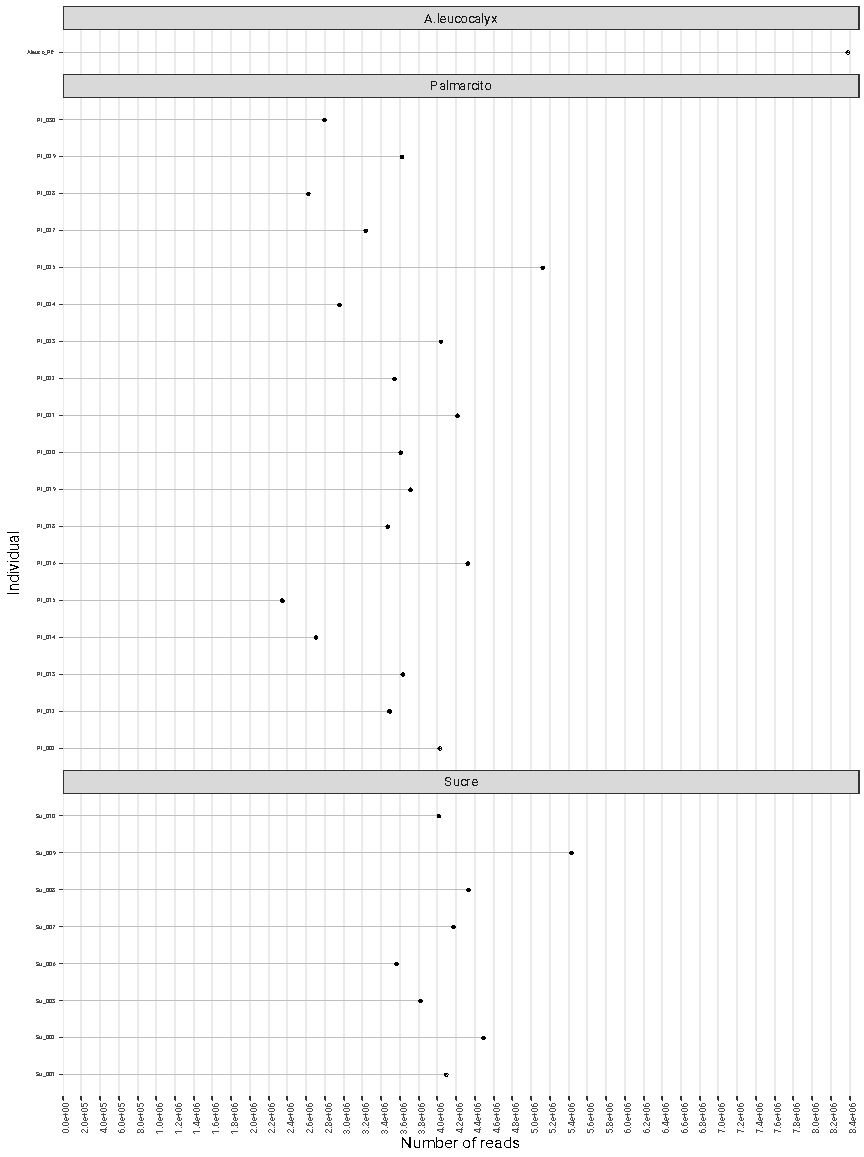
**

**Figure S2**. Comparison of bar plots showing the genetic structure of both *Parajubaea* species combined using the complete (left) and reduced (right) *de novo* SNP set for *K*=2 to *K*=7

| **Complete *de novo* SNP set (all SNPs = 15,134)** | **Reduced *de novo* SNP set (one SNP/locus = 4,710)** |
| --- | --- |
| 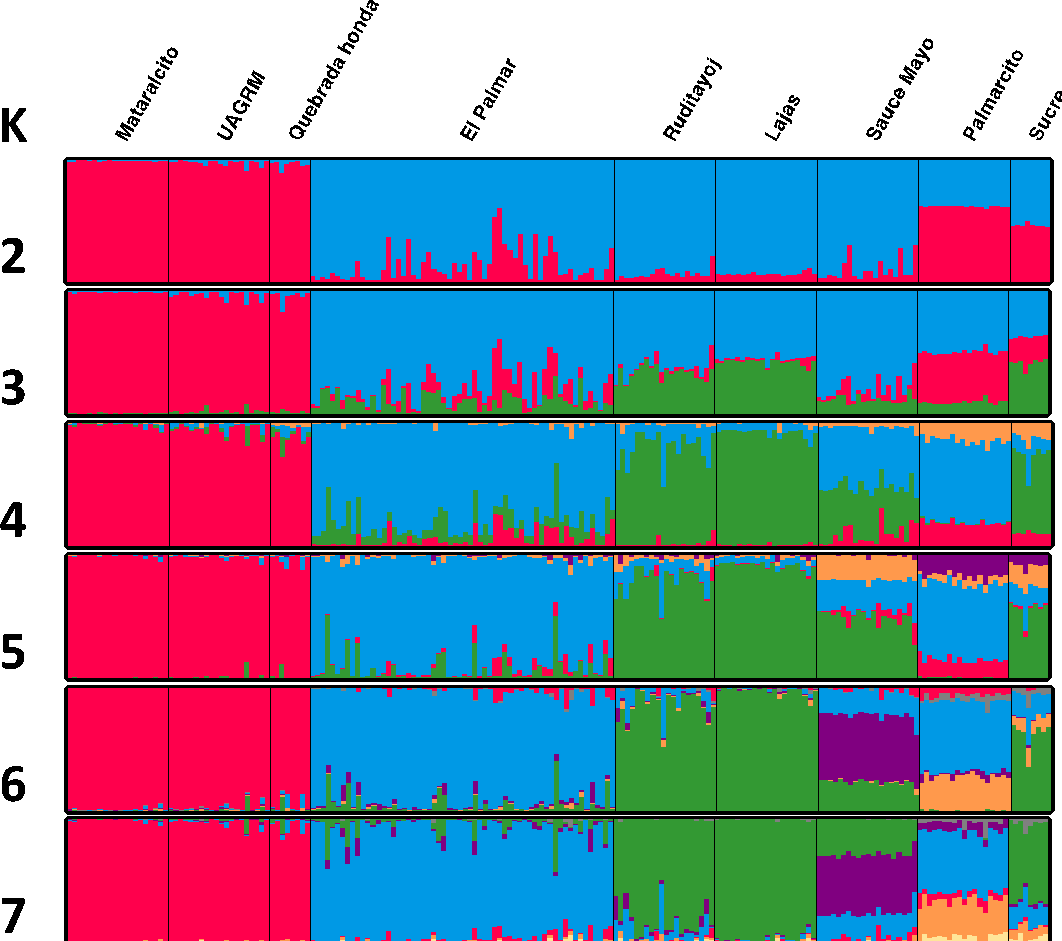 | 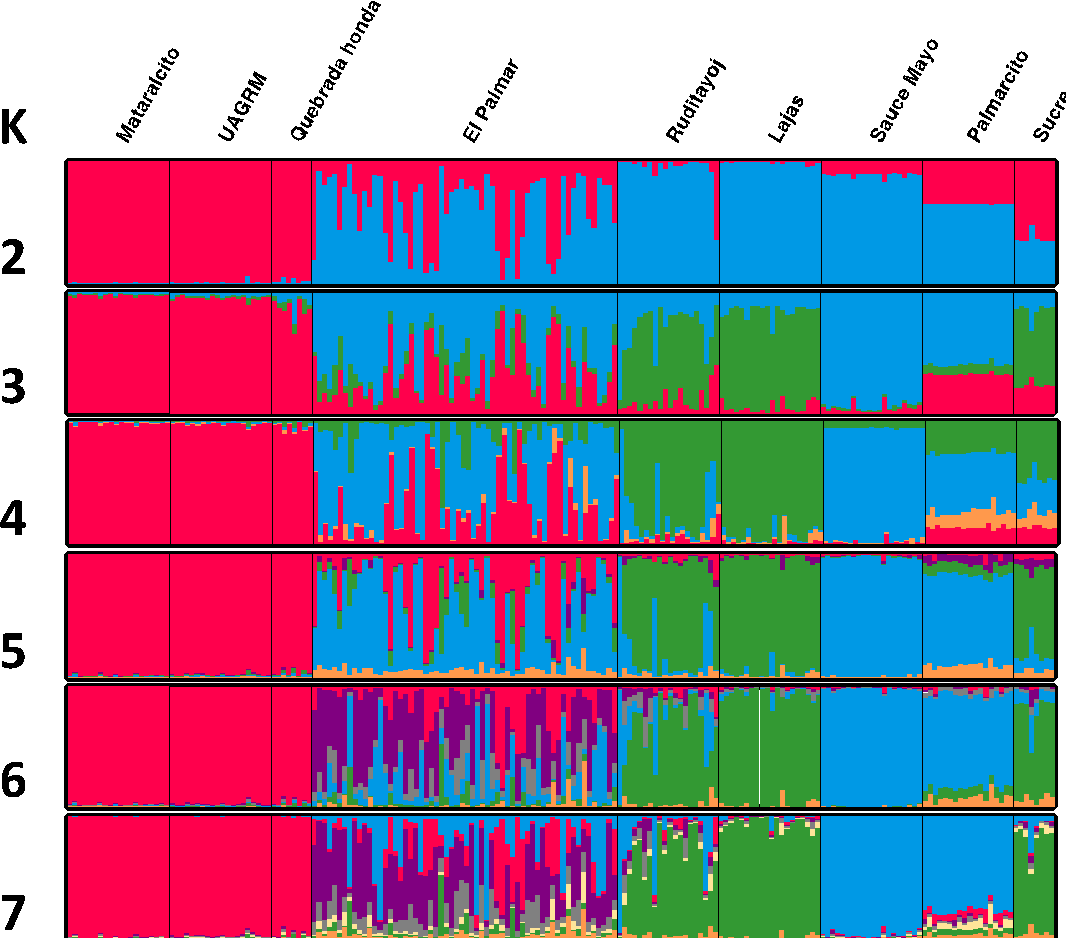 |

**Figure S3.** Likelihood and Evanno tests of the models evaluated in *STRUCTURE* using the complete *de novo* SNP set A) analysis of *P. sunkha* and *P. torallyi* combined, B) analysis of *P. torallyi* samples only, C) analysis of *P. sunkha* samples only.

A)


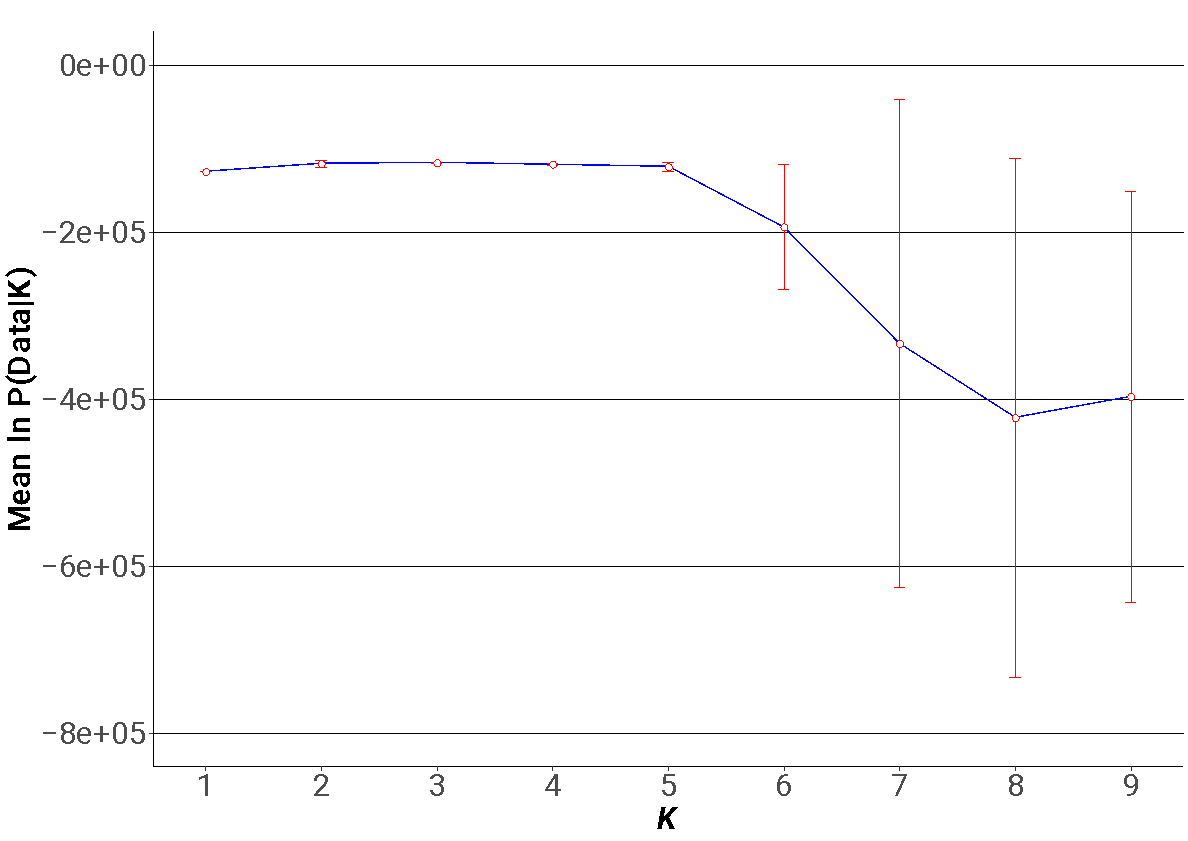

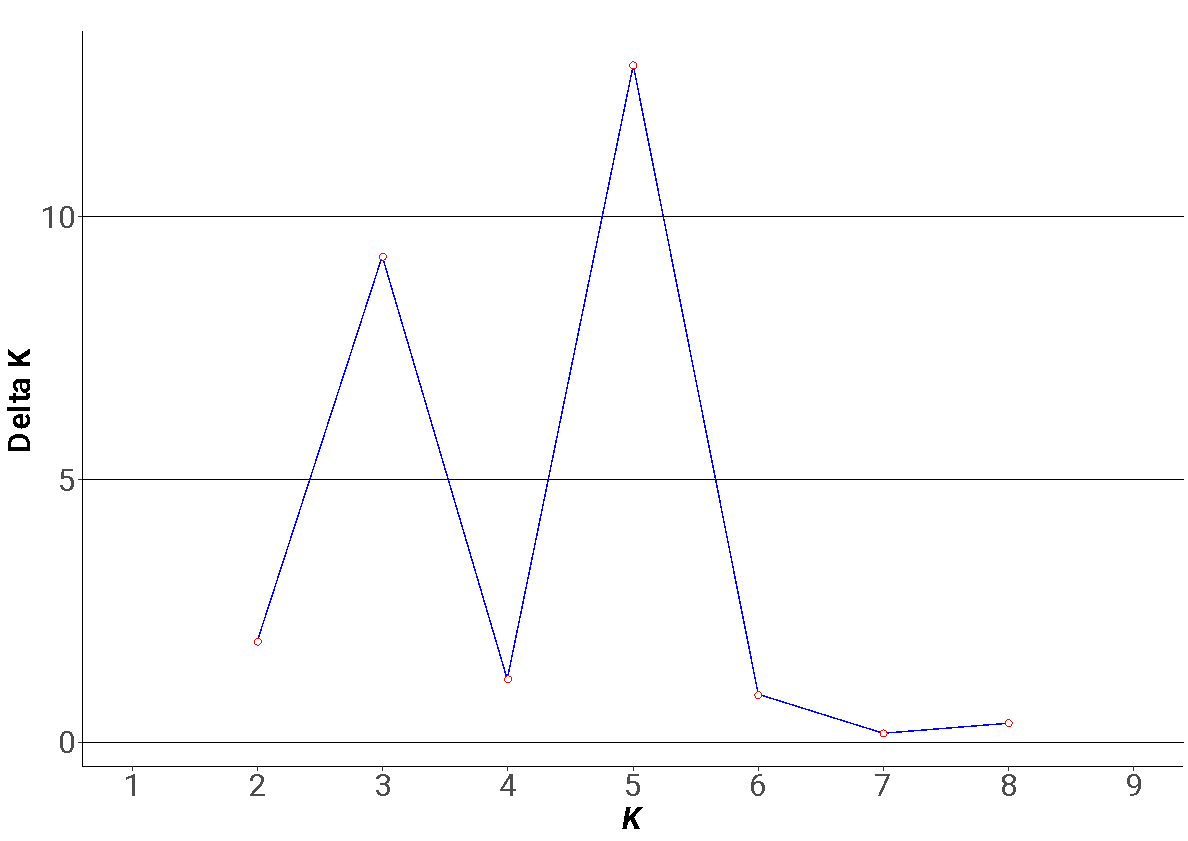


B)


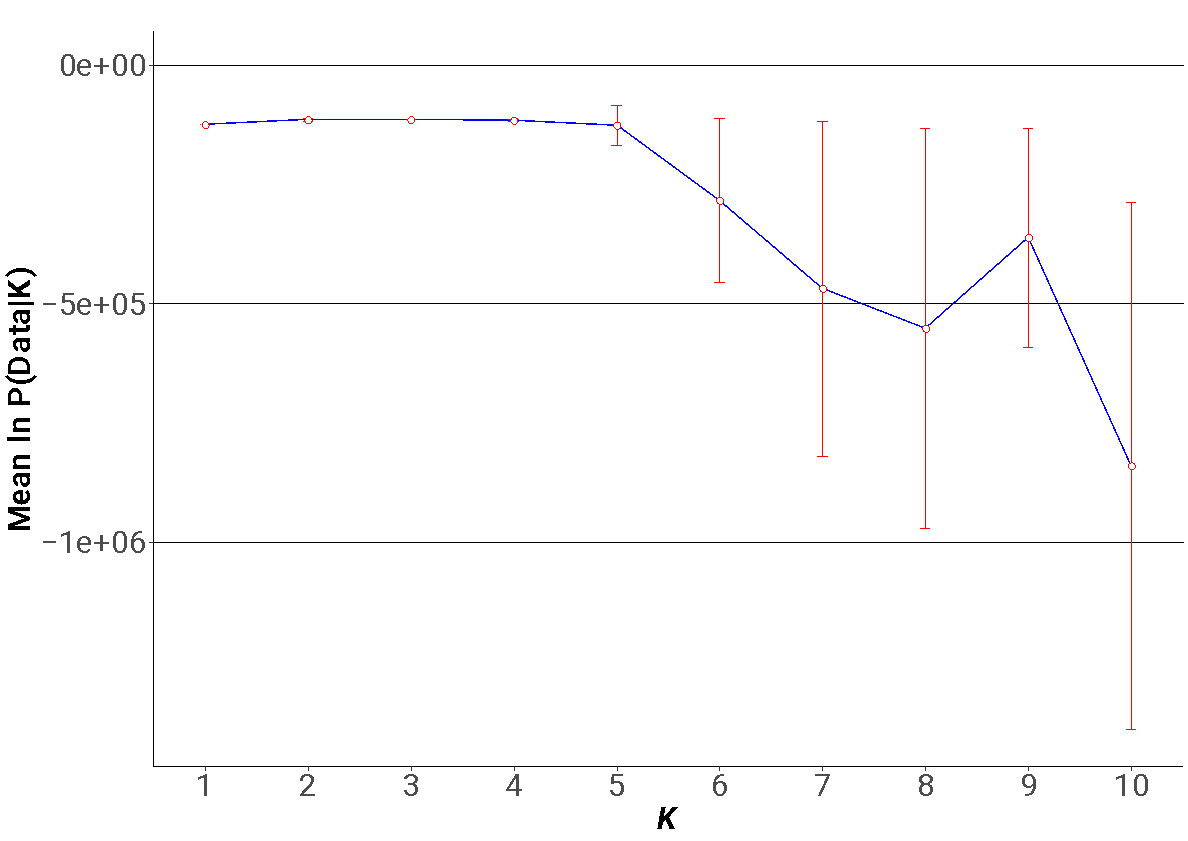

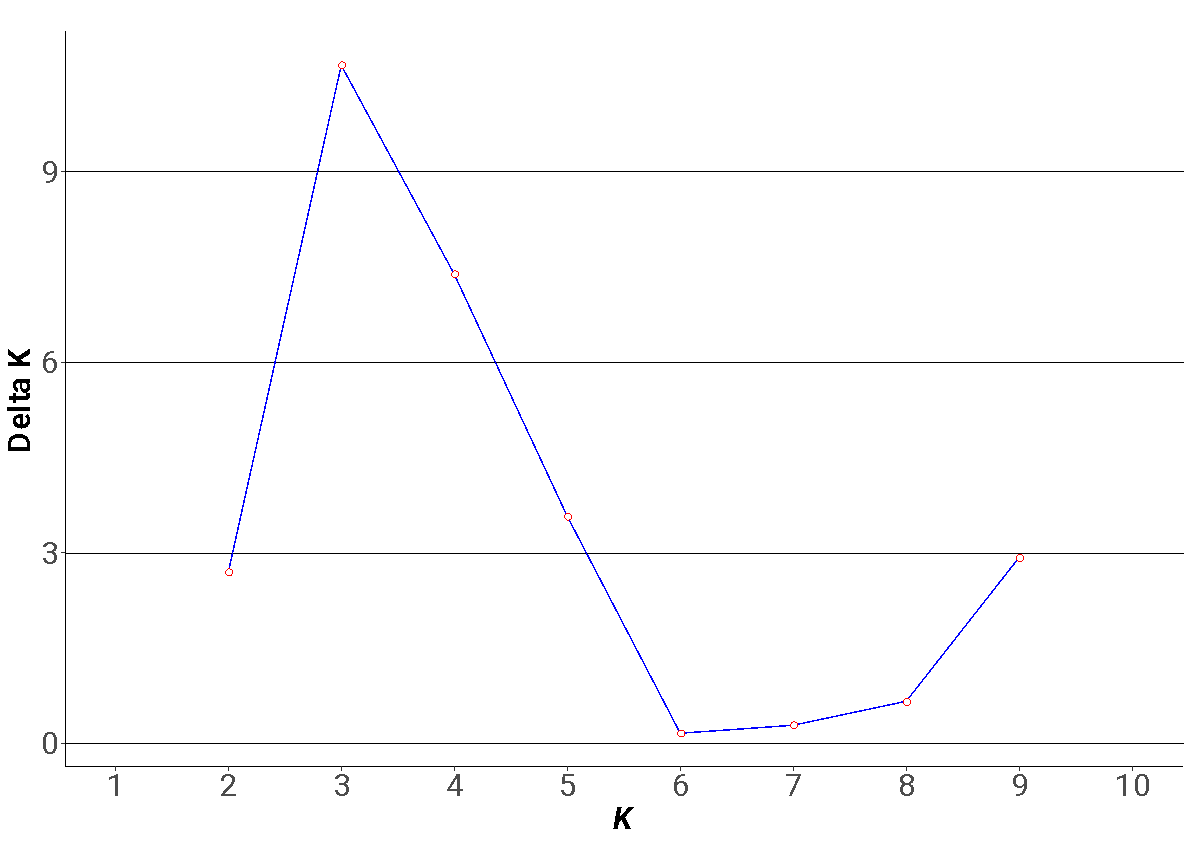


C)


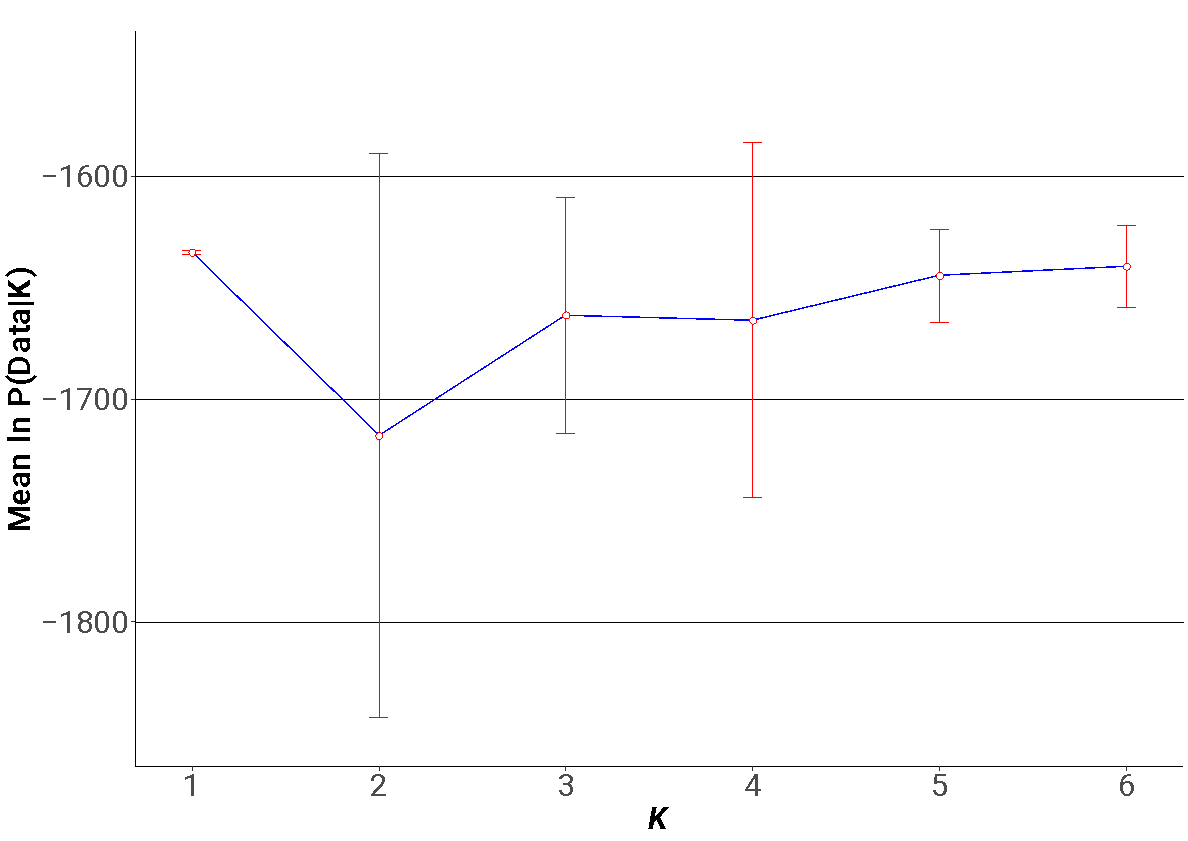

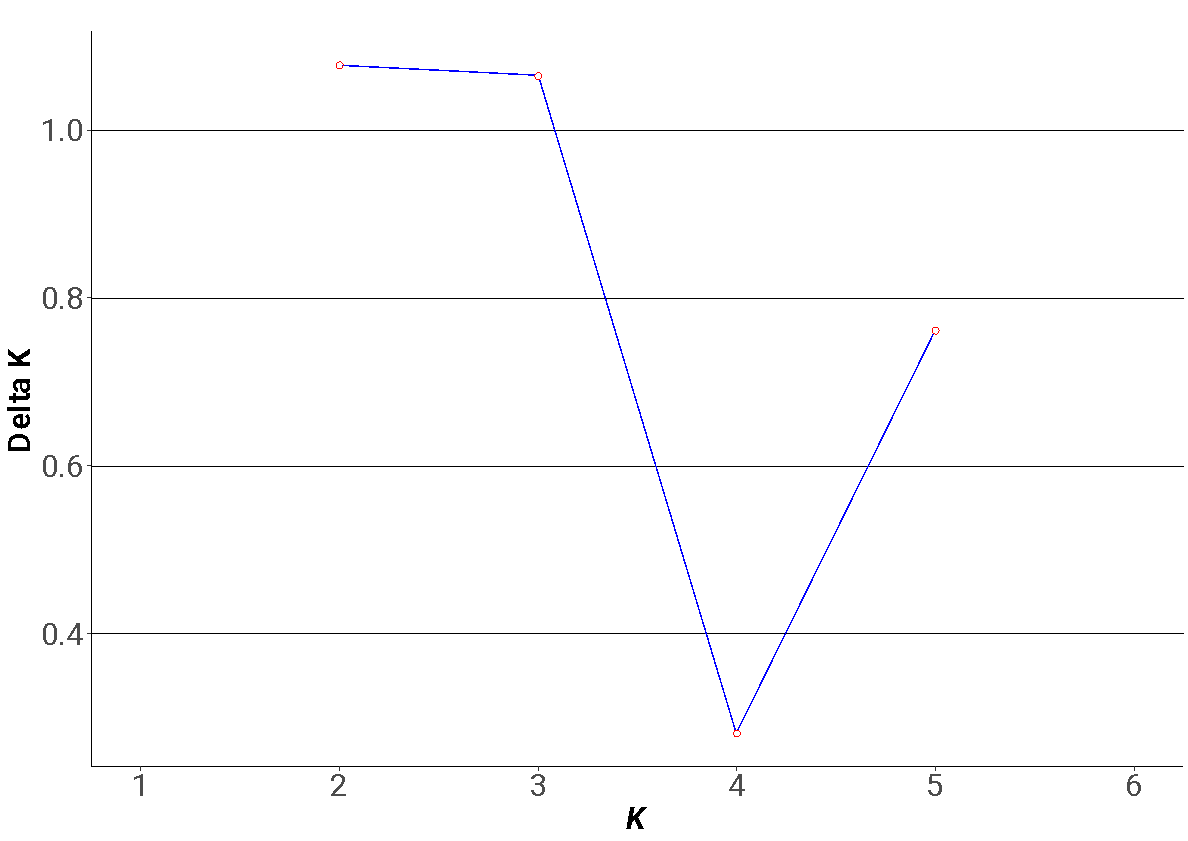


**Figure S4.** Comparison of bar plots showing the genetic structure of *Parajubaea torallyi* in Bolivia using the complete (left) and reduced (right) *de novo* SNP sets for *K*=2 to *K*=7. Sample locality names appear at the top of the bar plots.

| 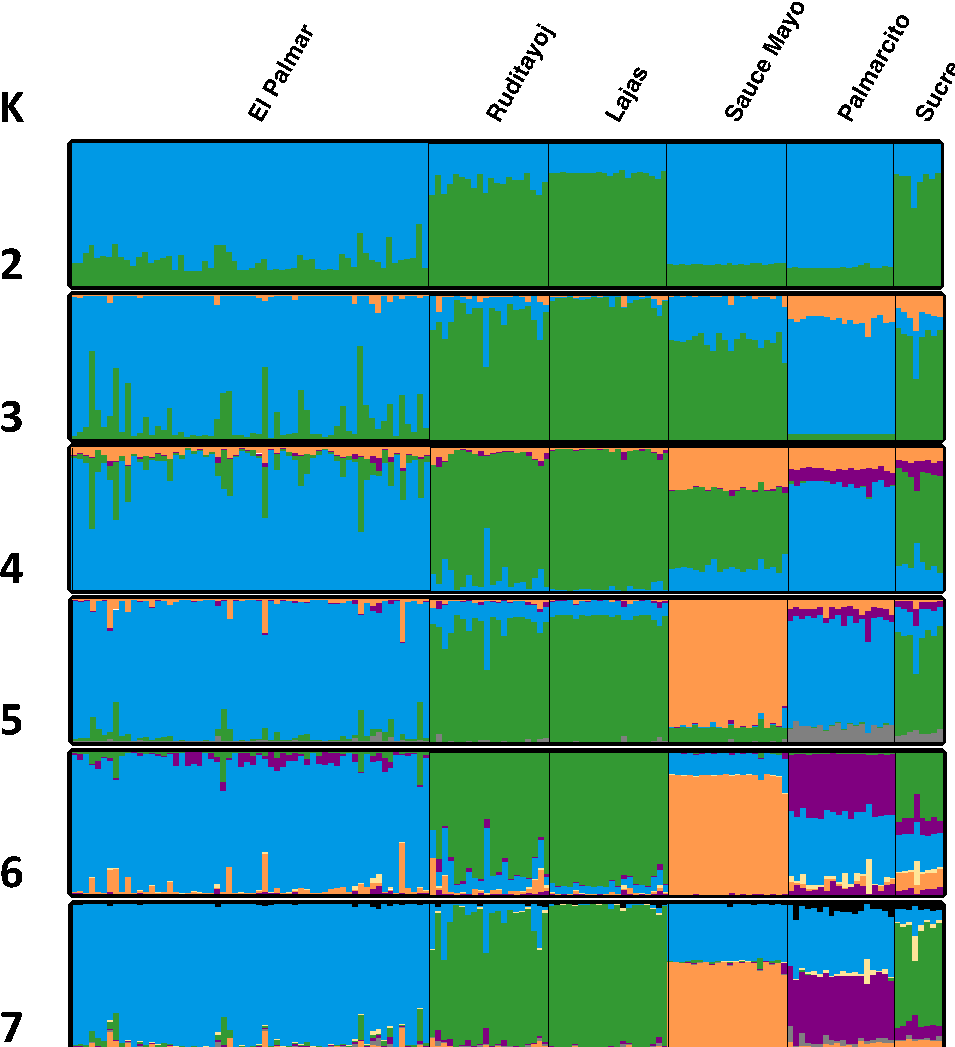 | 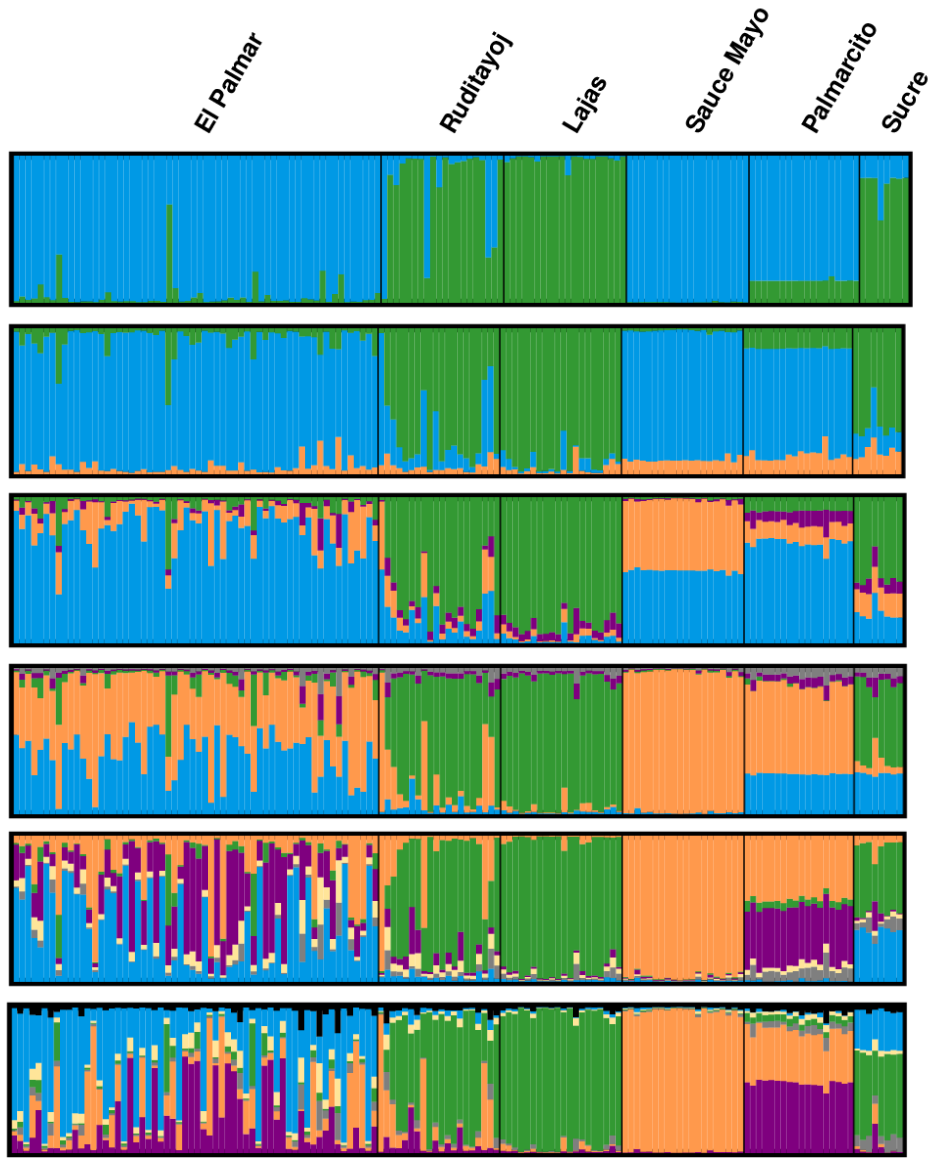 |
| --- | --- |

**Figure S5.** Comparison of bar plots showing the genetic structure of *Parajubaea sunkha* in Bolivia using the complete (left) and reduced (right) *de novo* SNP for *K*=2 to *K*=6. Sample locality names appear at the top of the bar plots.

| **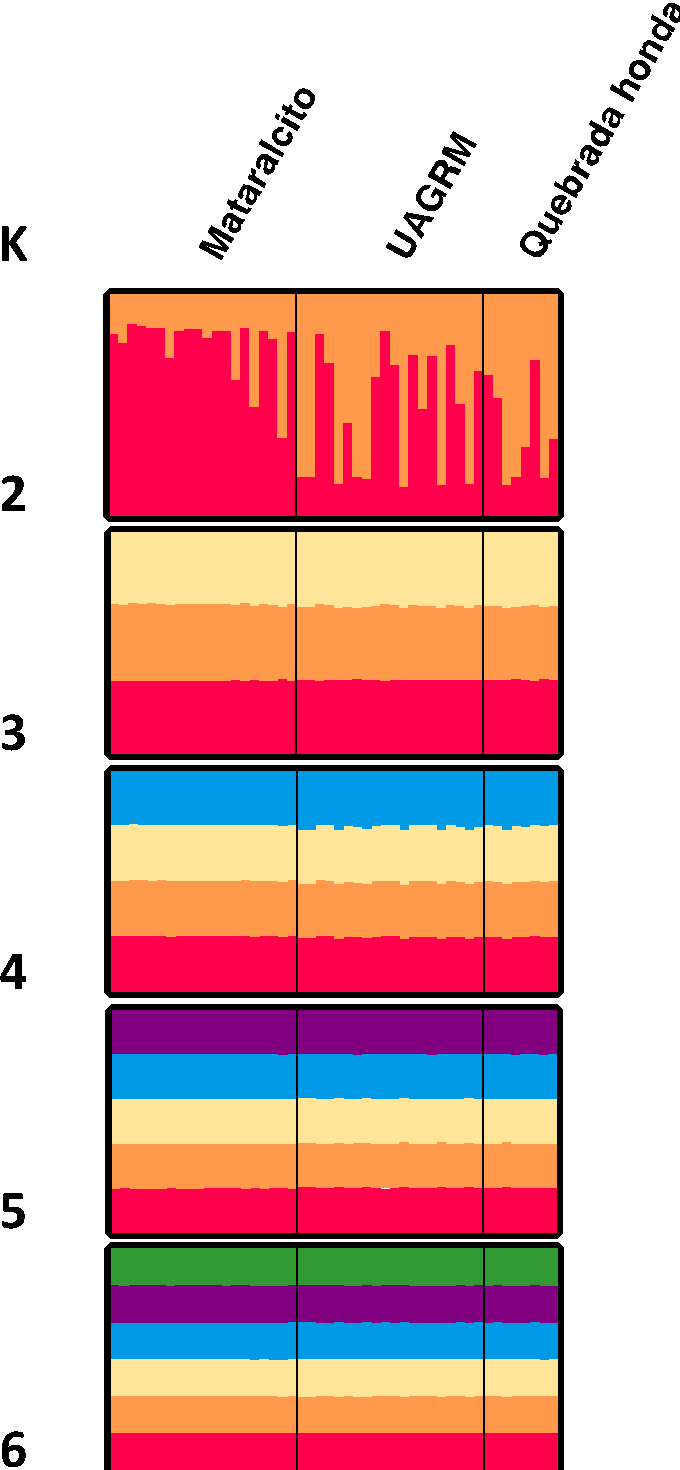** | 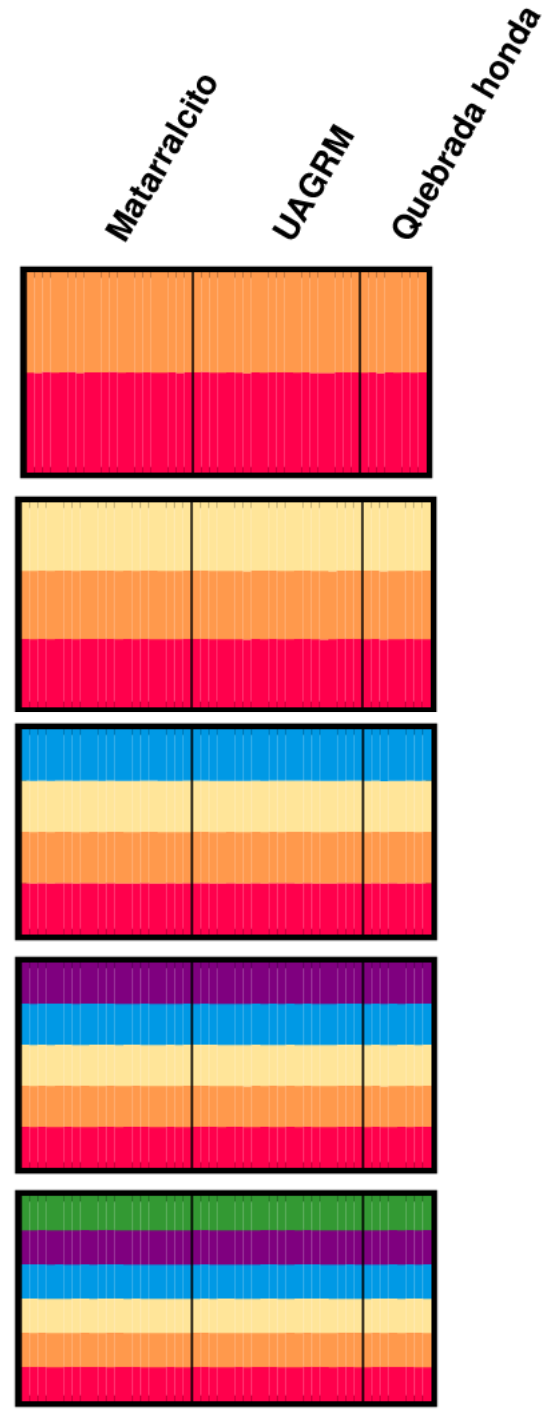 |
| --- | --- |

**Figure S6.** Visualisation of the discriminant analysis of principal components (DAPC) based on the complete *de novo* SNP set. Individuals are coloured by collection site (panels A, B and C) or according to their genetic group assignment (score ≥ 0.80) from the *STRUCTURE* analysis (panel D). Triangles are for *P. sunkha* and circles for *P. torallyi*. A) Analysis with both *Parajubaea* species combined showing the first and second discriminant functions. B) Same analysis as in (A) but showing the second and third discriminant functions. C) Analysis for *P. torallyi* alone showing the first and second discriminant functions. D) Same analysis as in (C) but showing the genetic group each individual was assigned to. Individuals in grey are admixed, attaining an assignment score lower than 0.80 for any of the genetic groups.

| 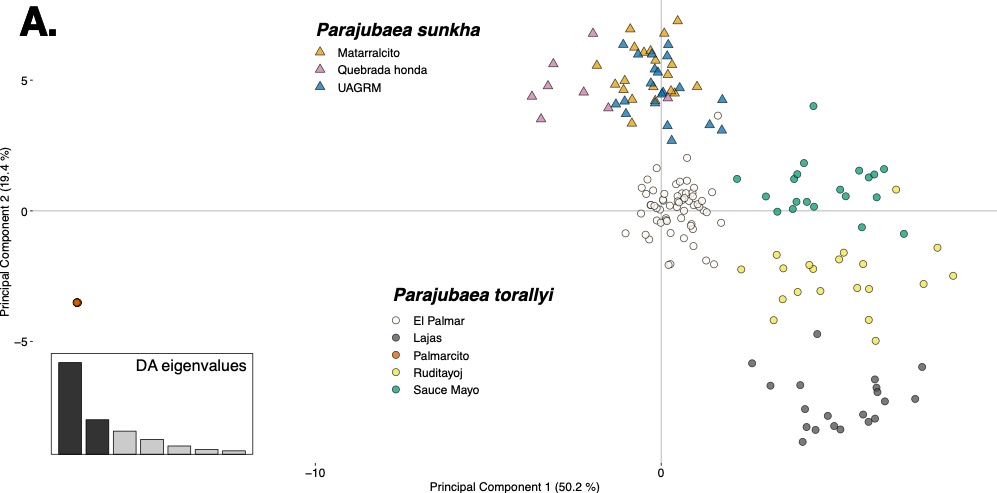 | 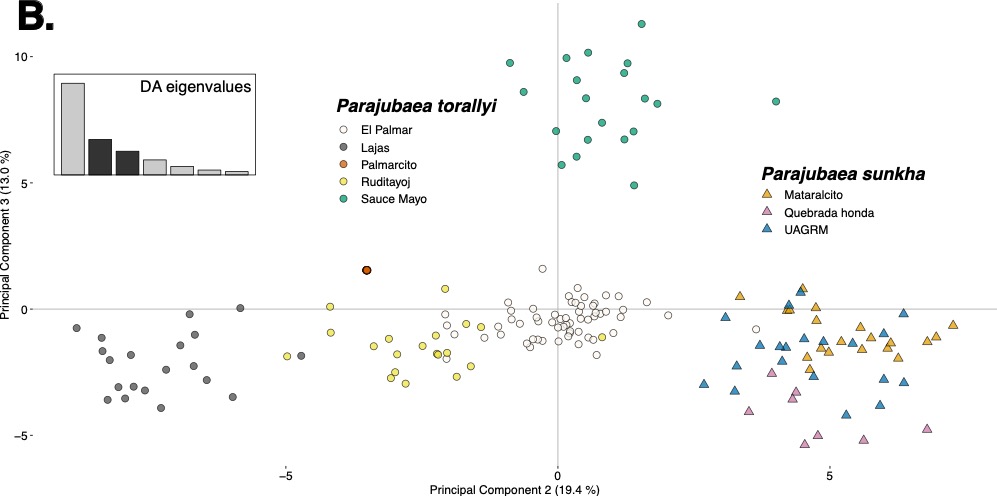 |
| --- | --- |
| 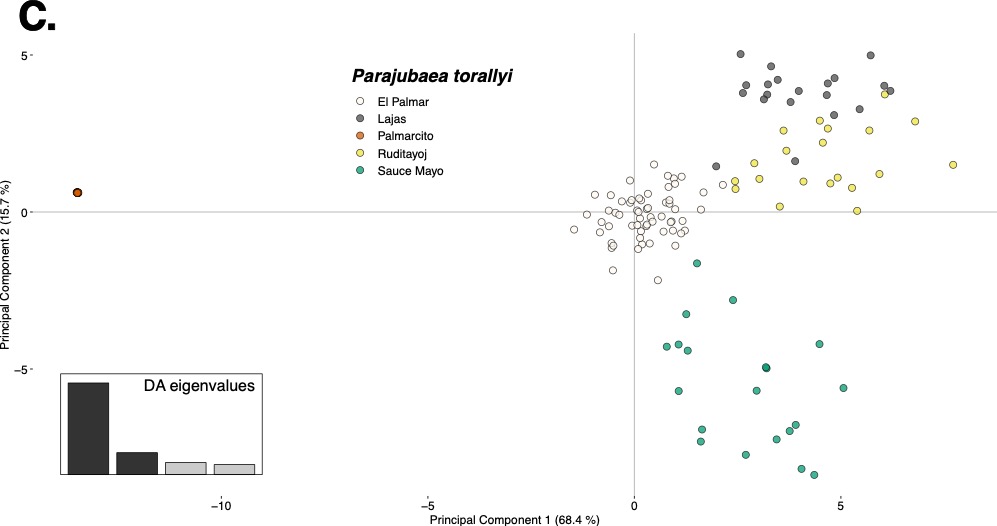 | 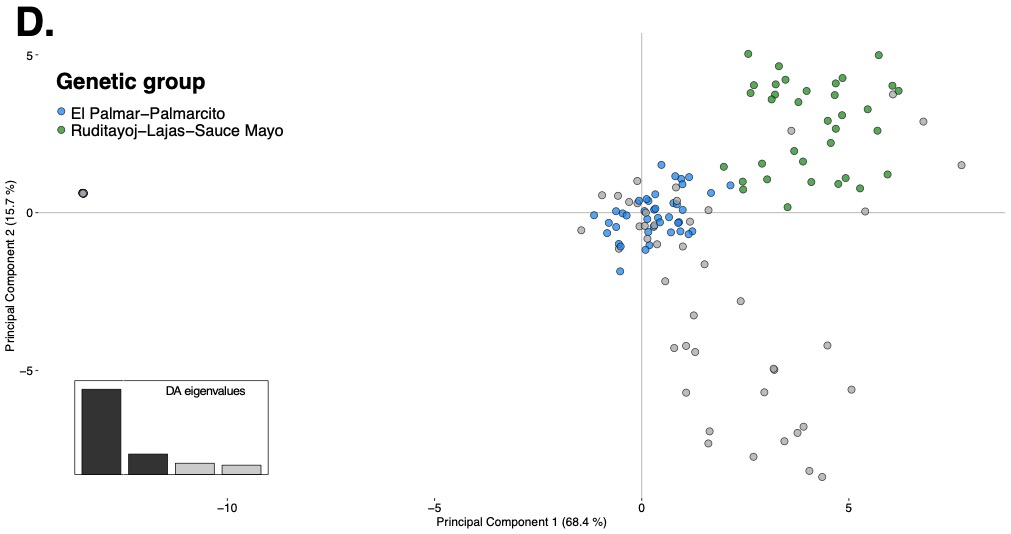 |

**Figure S7.** Visualisation of the discriminant analysis of principal components (DAPC) based on the pseudo reference SNP set with four different values of the *R* parameter in *Stacks* (20, 40, 60, and 80) with and without imputation. Individuals are coloured by collection site. Triangles are for *P. sunkha* and circles for *P. torallyi*.

| R | No Imputation | Imputation |
| --- | --- | --- |
| 20 | 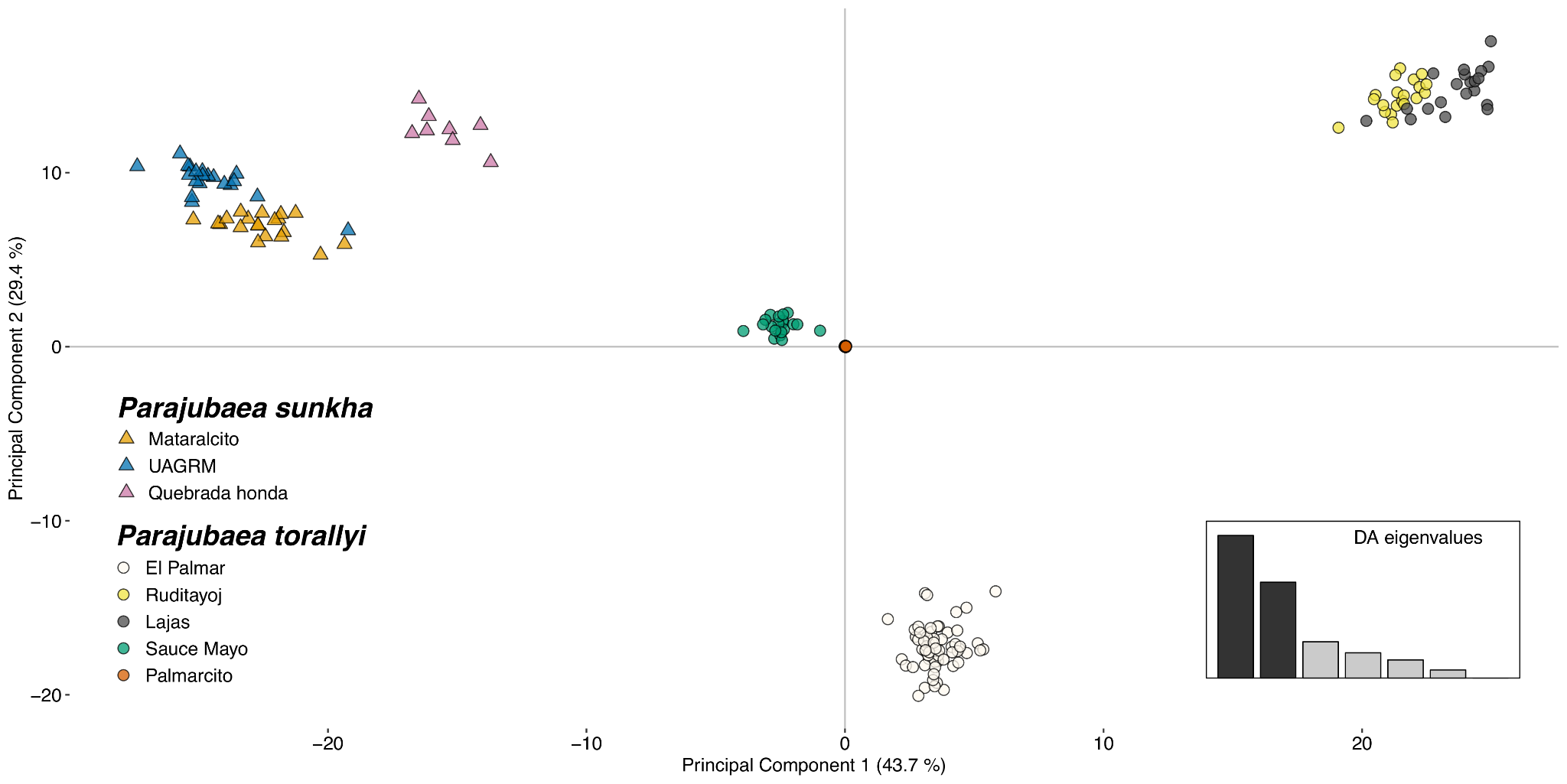 | 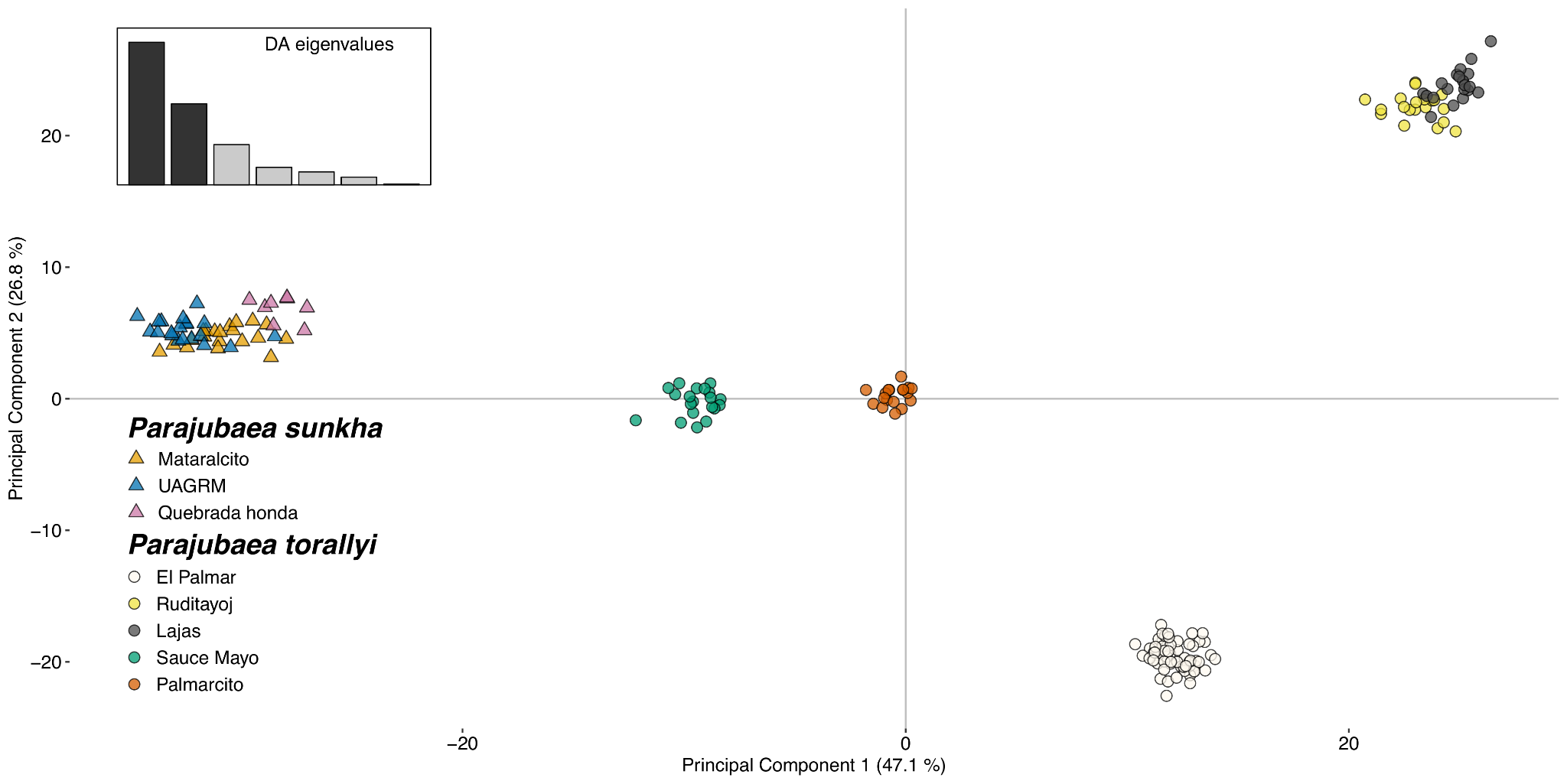 |
| 40 | 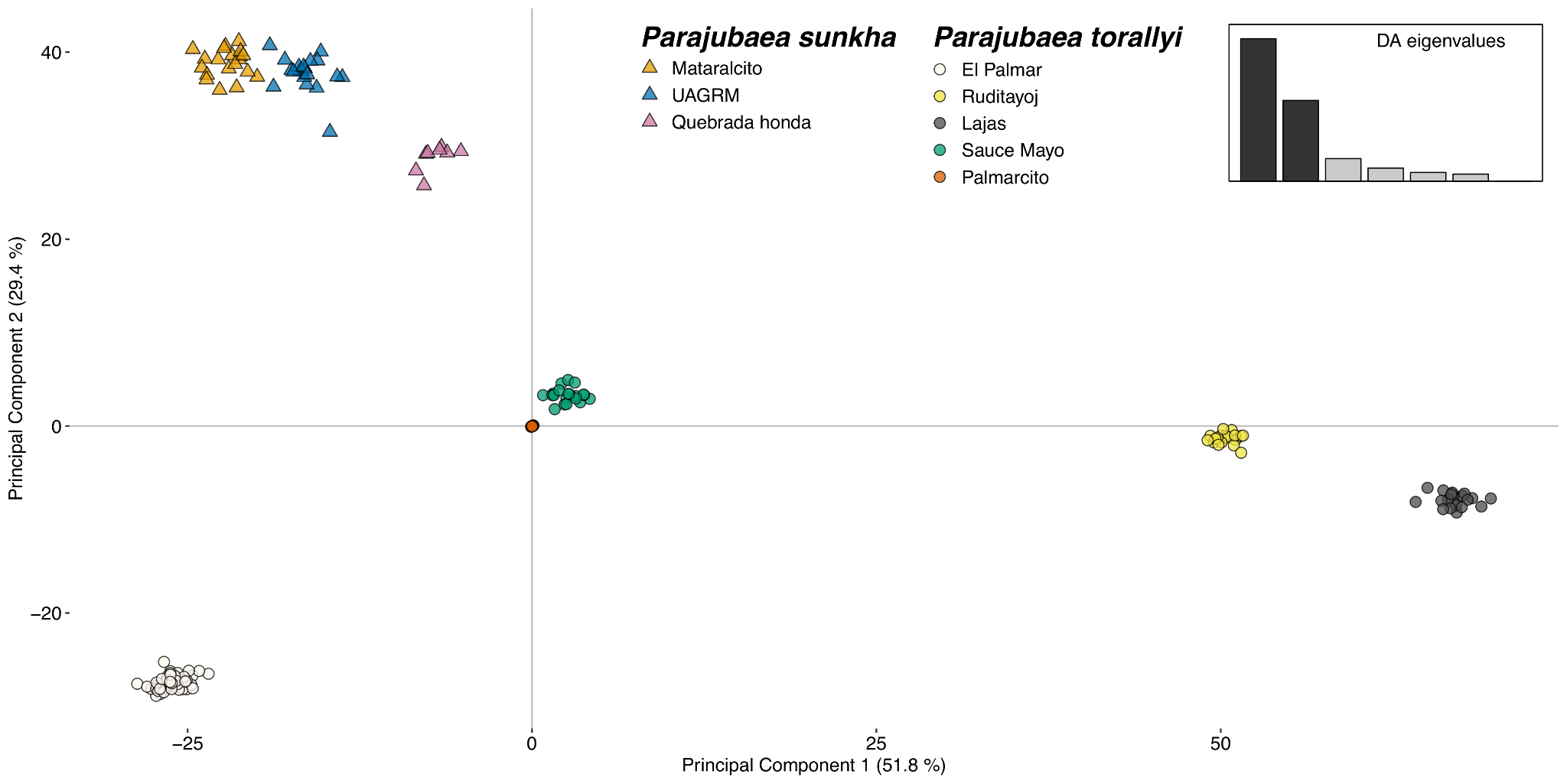 | 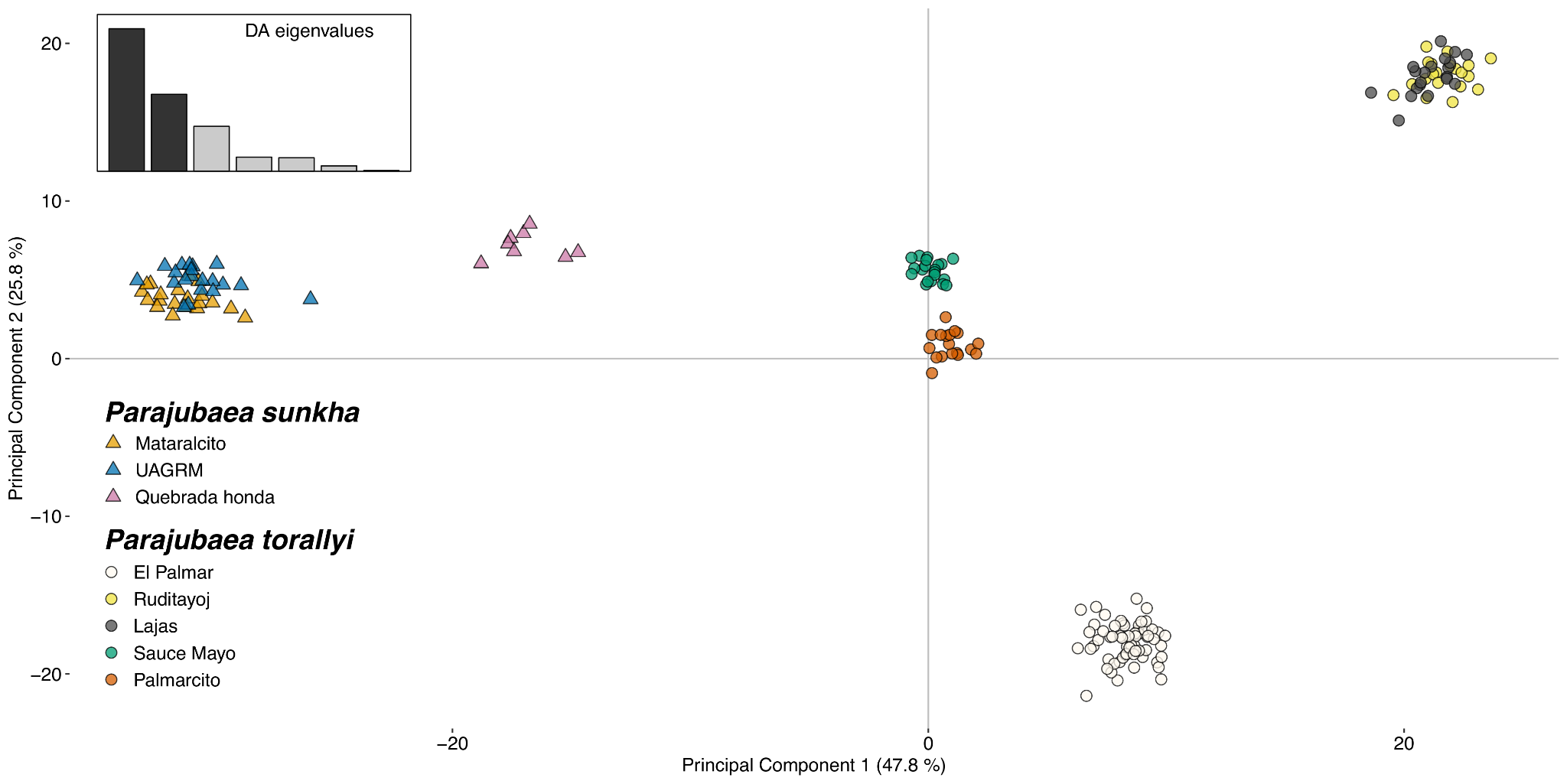 |
| 60 | 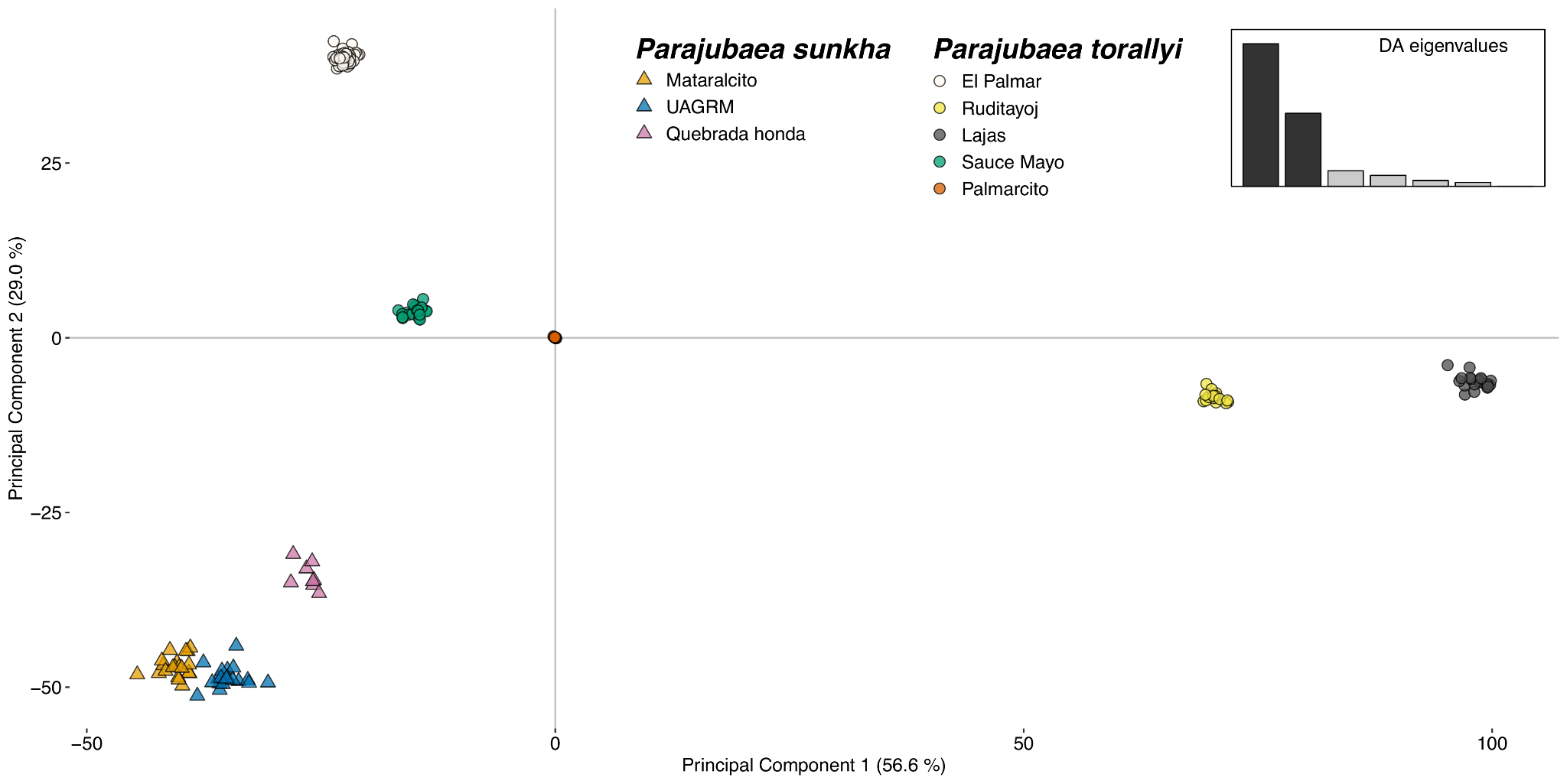 | 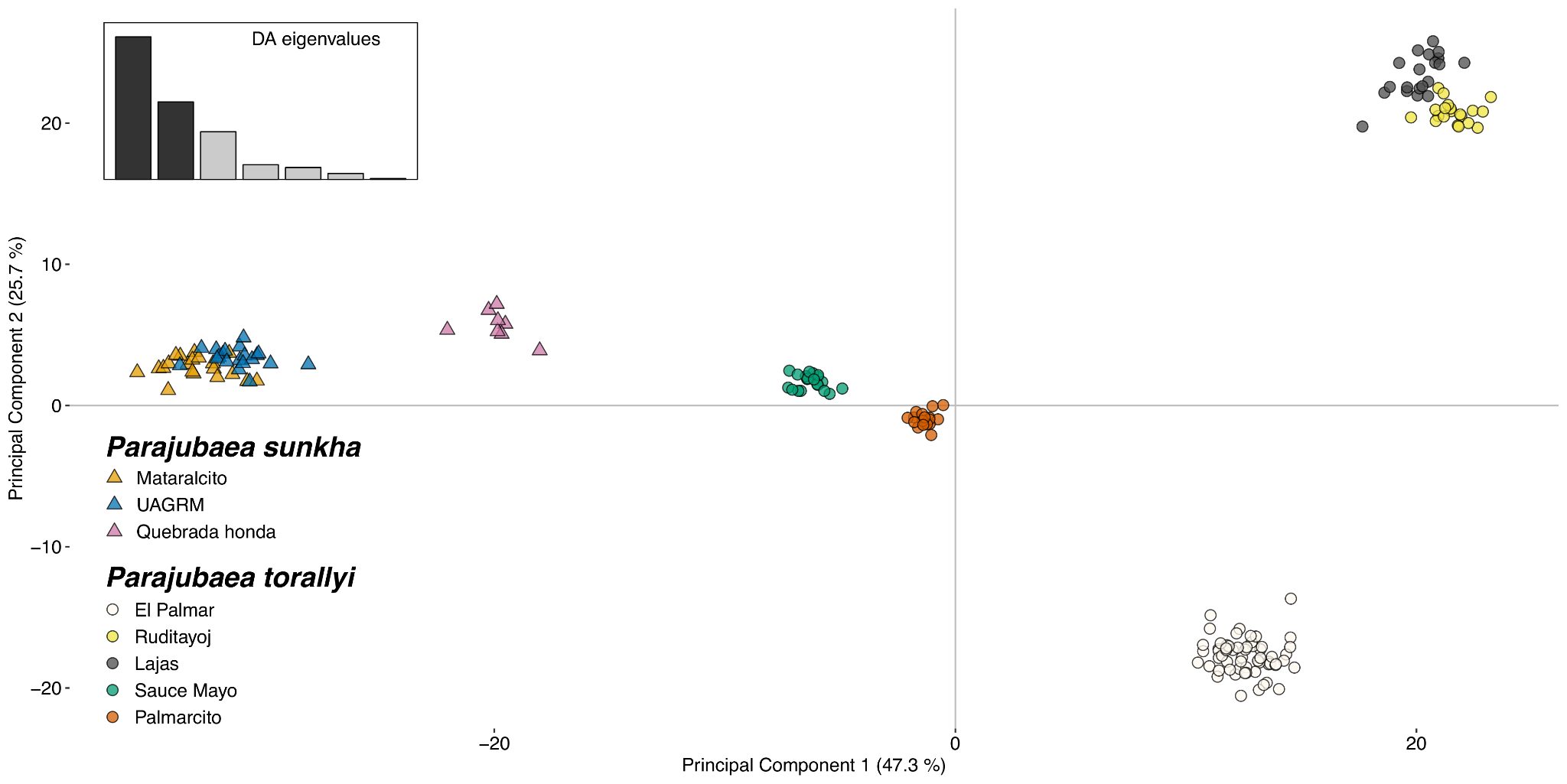 |
| 80 | 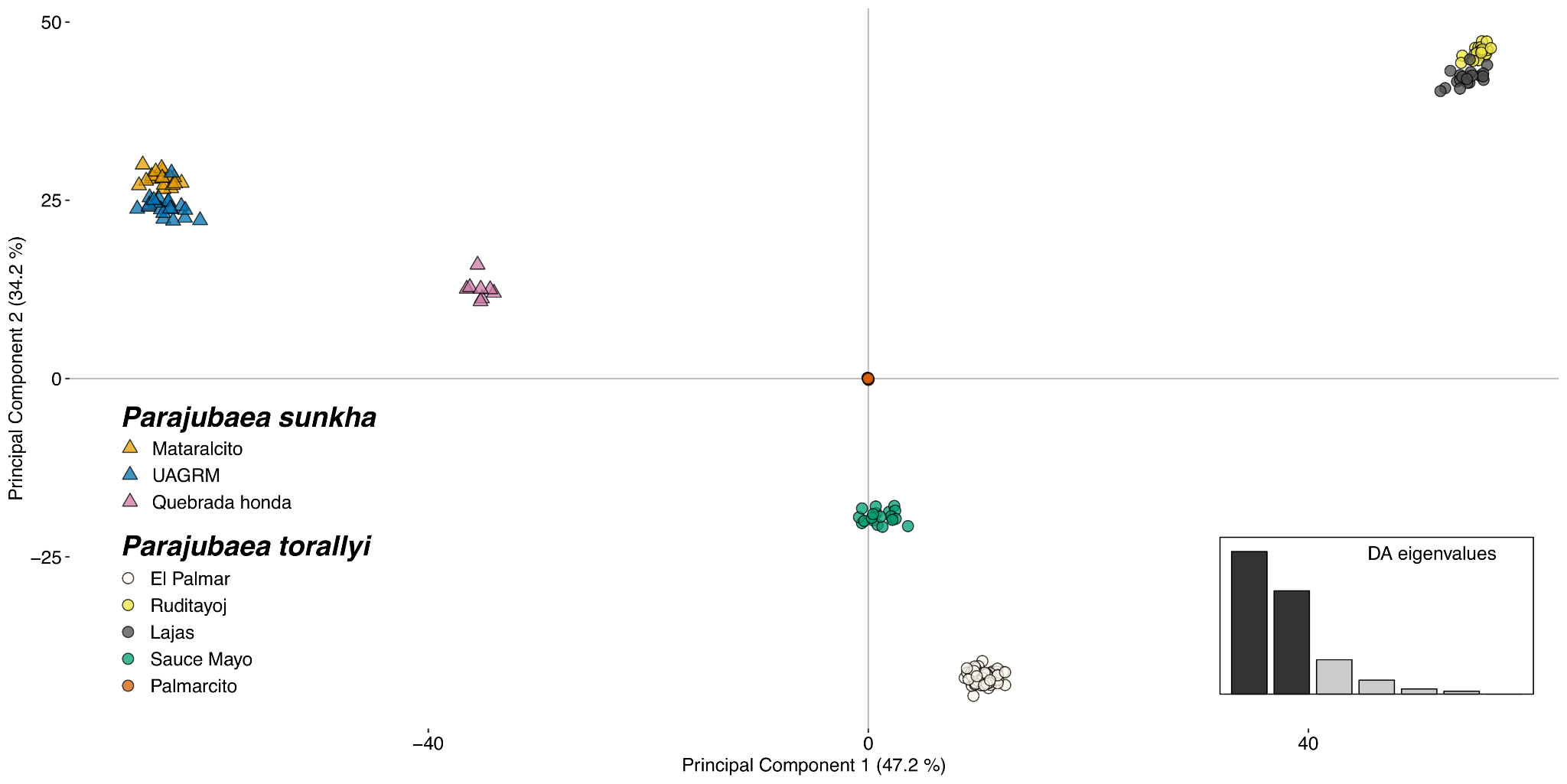 | 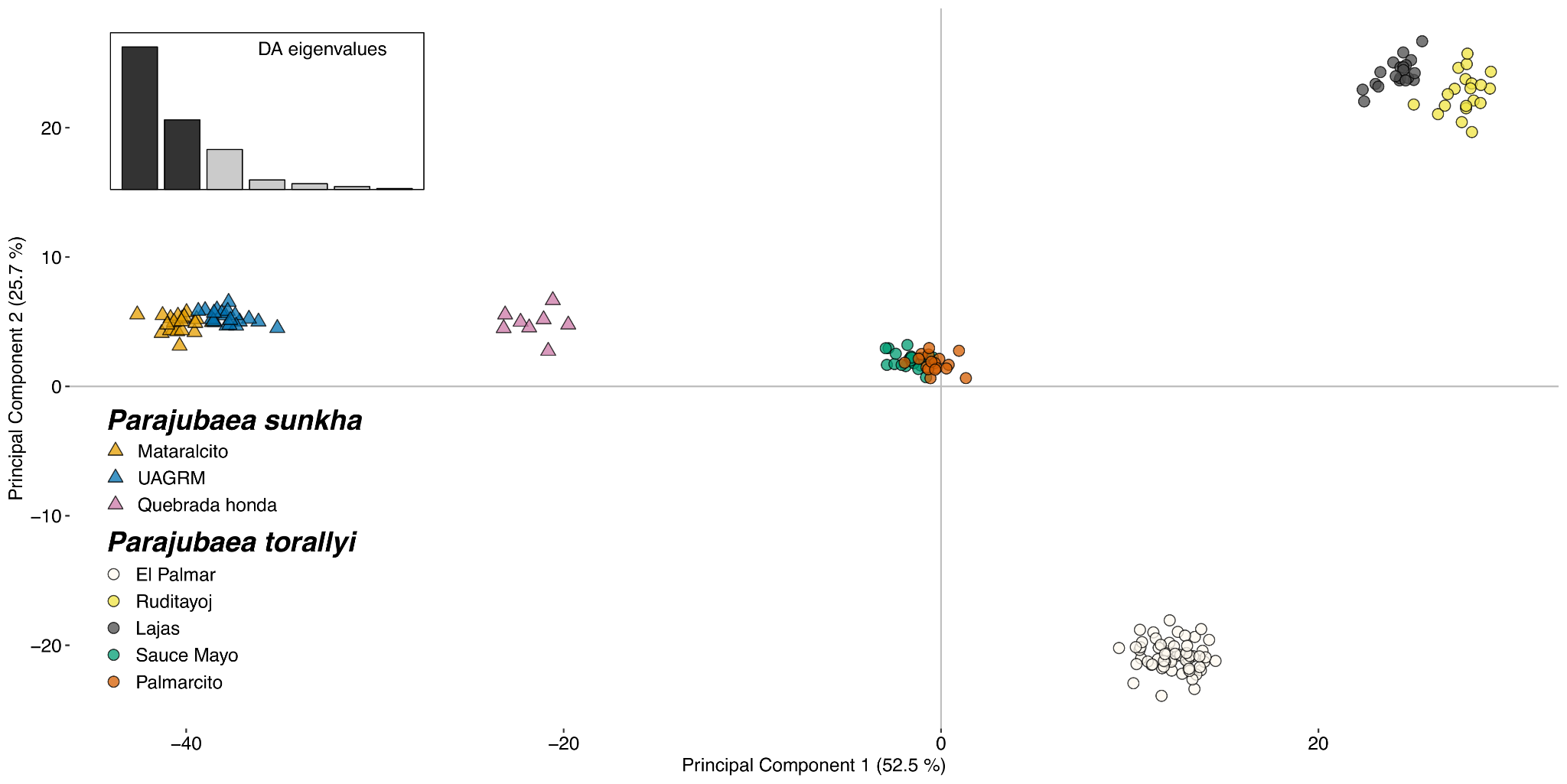 |

**References**

Borchsenius, F., Borgtoft Pedersen, H., & Balslev, H. (1998). Manual to the palms of Ecuador. *AAU Reports, 37*, 1-211.

Ersts, P. J. (2011). Geographic distance matrix generator v1.2.3. American Museum of Natural History, Center for Biodiversity and Conservation. h[ttp://biodiversityinformatics.amnh.org/open_source/gdmg](about:blank)

Lou, R. N., & Therkildsen, N. O. (2022). Batch effects in population genomic studies with low-coverage whole genome sequencing data: Causes, detection and mitigation. *Molecular Ecology Resources*, 22, 1678–1692. <https://doi.org/10.1111/1755-0998.13559>

Meerow, A. W., Noblick, L., Salas-Leiva, D. E., Sanchez, V., Francisco-Ortega, J., Jestrow, B., & Nakamura, K. (2015). Phylogeny and historical biogeography of the cocosoid palms (Arecaceae, Arecoideae, Cocoseae) inferred from sequences of six WRKY gene family loci. *Cladistics, 31*(5), 509-534. <https://doi.org/10.1111/cla.12100>

Moraes R., M. (2020). Flora de palmeras de Bolivia. Segunda edición. Herbario Nacional de Bolivia, Instituto de Ecología, Universidad Mayor de San Andrés, Plural editores, La Paz. 458 p.

Moraes R., M., & Henderson, A. (1990). The genus *Parajubaea* (Palmae). *Brittonia, 42*(2), 92-99. <https://doi.org/10.2307/2807619>
